# A fast and simple LC-MS-based characterization of the flavonoid biosynthesis pathway for few seed(ling)s

**DOI:** 10.1186/s12870-016-0880-7

**Published:** 2016-09-01

**Authors:** Benjamin Jaegle, Miran Kalle Uroic, Xu Holtkotte, Christina Lucas, Andreas Ole Termath, Hans-Günther Schmalz, Marcel Bucher, Ute Hoecker, Martin Hülskamp, Andrea Schrader

**Affiliations:** 1 Botanical Institute and Cluster of Excellence on Plant Sciences (CEPLAS), University of Cologne, Cologne Biocenter, Zülpicher Str. 47b, 50674 Cologne, Germany; 2 Department of Chemistry, University of Cologne, Greinstr. 4, 50939 Cologne, Germany

**Keywords:** Anthocyanidin, Proanthocyanidin, Flavonoids, Seed, Seedling, Deuterated internal standard, LC-MS, Hydrolysis, *Arabidopsis thaliana*

## Abstract

**Background:**

(Pro)anthocyanidins are synthesized by the flavonoid biosynthesis pathway with multi-layered regulatory control. Methods for the analysis of the flavonoid composition in plants are well established for different purposes. However, they typically compromise either on speed or on depth of analysis.

**Results:**

In this work we combined and optimized different protocols to enable the analysis of the flavonoid biosynthesis pathway with as little as possible biological material. We chose core substances of this metabolic pathway that serve as a fingerprint to recognize alterations in the main branches of the pathway. We used a simplified sample preparation, two deuterated internal standards, a short and efficient LC separation, highly sensitive detection with tandem MS in multiple reaction monitoring (MRM) mode and hydrolytic release of the core substances to reduce complexity. The method was optimized for *Arabidopsis thaliana* seeds and seedlings. We demonstrate that one Col-0 seed/seedling is sufficient to obtain a fingerprint of the core substances of the flavonoid biosynthesis pathway. For comparative analysis of different genotypes, we suggest the use of 10 seed(lings). The analysis of *Arabidopsis thaliana* mutants affecting steps in the pathway revealed foreseen and unexpected alterations of the pathway. For example, HY5 was found to differentially regulate kaempferol in seeds vs. seedlings. Furthermore, our results suggest that COP1 is a master regulator of flavonoid biosynthesis in seedlings but not of flavonoid deposition in seeds.

**Conclusions:**

When sample numbers are high and the plant material is limited, this method effectively facilitates metabolic fingerprinting with one seed(ling), revealing shifts and differences in the pathway. Moreover the combination of extracted non-hydrolysed, extracted hydrolysed and non-extracted hydrolysed samples proved useful to deduce the class of derivative from which the individual flavonoids have been released.

**Electronic supplementary material:**

The online version of this article (doi:10.1186/s12870-016-0880-7) contains supplementary material, which is available to authorized users.

## Background

Flavonoids play a role in a wide variety of biological phenomena. In plants, their bright colors attract pollinators while their antioxidant properties offer protection from harmful UV-radiation. For humans, proanthocyanidins from red wine have been discussed to explain the “French paradox” – the co-occurrence of low coronary heart disease deaths and a diet rich in saturated fat [1–3]. In particular the medical implications led to extensive studies on their uptake, metabolism and excretion in animals and humans [4, 5]. Flavonoids are phenylpropanoid-derived secondary metabolites that may accumulate in various plant tissues. Their production is often regulated by environmental factors including light, temperature, pathogen attack and nutrient deprivation. Flavonoids represent a complex group of compounds. The major subgroups comprise chalcones, flavones, flavonols, flavandiols, anthocyanidins and proanthocyanidins [6].

As most structural genes of the pathway are monogenic in *Arabidopsis thaliana (A. thaliana)* [3], this model species is well suited to analyse the flavonoid core biosynthesis. The underlying genetic loci of structural and regulatory genes were mainly derived from mutant screenings for reduced seed coat pigmentation and were initially named *TRANSPARENT TESTA* (*TT*) [7, 8].

**Fig. 1:**
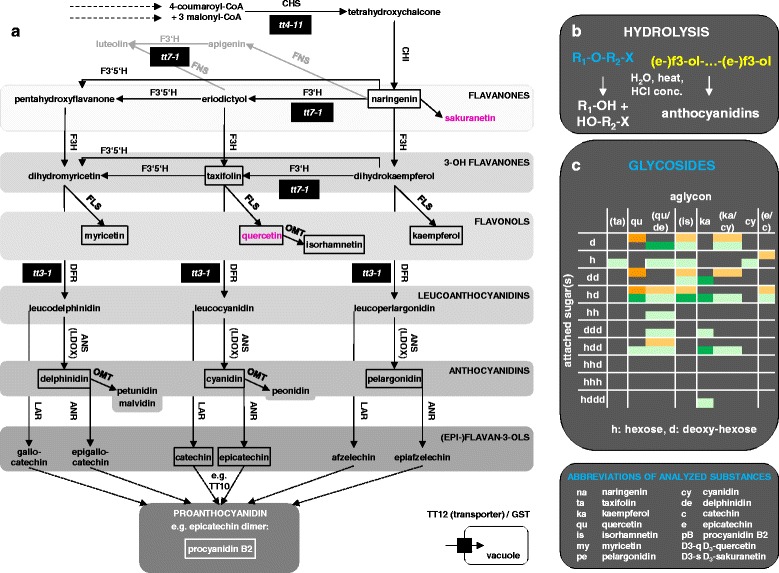
Flavonoid biosynthesis pathway and the concept of hydrolysis to reduce complexity of extracts. **a** Flavonoid core pathway of plants. Unfilled boxes: external standards. Filled boxes: analysed mutants for enzymes. Pink: deuterated internal standards. Examples for downstream enzymes and substances for the branching point naringenin and eriodictyol are shown in grey. **b** Examples for hydrolysed ether bonds in flavonoid derivatives and cleaved interflavan bonds in proanthocyanidins. For glycosylated flavonoids: R_1_: aglycon (flavonoid); R_2_: sugar; X: possible additional modification. (e-)f3-ol: (epi-)flavan-3-ol. **c** Glycosylated flavonoids from methanolic extracts of seeds (orange) and seedlings (green). Dark color: identified glycosides based on LC-ESI-MS-QTOF(AutoMSMS) and LC-ESI-MS-QTOF(pseudoMS^3^). Light color: predicted glycosides without further fragmentation of the aglycon in the pseudoMS^3^ experiment. The order and position of the attached sugar moieties was not specified. In brackets: the aglycon is predicted and not identified. In these cases there was no differentiation for qu/de, ka/cy and e/c with same m/z values, respectively. See Additional file 2: Tables S3-S5 for details. Enzymes: CHS: CHALCONE SYNTHASE, CHI: CHALCONE ISOMERASE, FNS: FLAVONE SYNTHASE, F3H: FLAVANONE 3-HYDROXYLASE, F3’H: FLAVONOID 3' HYDROXYLASE, F3’5’H: FLAVONOID 3'5' HYDROXYLASE, FLS: FLAVONOL SYNTHASE, OMT: O-METHYLTRANSFERASE, DFR: DIHYDROFLAVONOL 4-REDUCTASE, ANS: ANTHOCYANIDIN SYNTHASE., LDOX: LEUCOANTHOCYANIDIN DIOXYGENASE, LAR: LEUCOANTHOCYANIDIN REDUCTASE, ANR: ANTHOCYANIDIN REDUCTASE, TT10: TRANSPARENT TESTA 10. Example for proteins relevant for subcellular deposition: TT12 (TRANSPARENT TESTA 12), GST (GLUTATHIONE S-TRANSFERASE). *tt3, 4*, *7*: *transparent testa 3, 4, 7*. The pathway was extracted from PlantCyc and extended with previous reviews [9, 84, 85]

CHALCONE SYNTHASE (CHS/TT4) catalyses the first committed step of the pathway (Fig. 1a): the synthesis of chalcone. Chalcone is isomerized to naringenin by CHALCONE ISOMERASE (CHI/TT5). Naringenin and eriodyctiol are branching points to other flavonoid classes. FLAVANONE 3-HYDROXYLASE (F3H/TT6) converts naringenin to the first compound of the next level in the plant’s flavonoid biosynthesis. This level comprises the 3-OH flavanones dihydrokaempferol, taxifolin and dihydromyricetin which are interconverted by FLAVONOID 3'-MONOOXYGENASE (F3’H, TT7) and FLAVONOID 3’,5’ HYDROXYLASE (F3’5’H). All three substances serve as educts for FLAVONOL SYNTHASE (FLS) and DIHYDROFLAVONOL 4-REDUCTASE (DFR/TT3) resulting in three further branches of the pathway. The flavonols kaempferol, quercetin and myricetin, are the products of FLS. The leucoanthocyanidins leucopelargonidin, leucocyanidin and leucodelphinidin are synthetized by DFR and further converted to the anthocyanidins pelargonidin, cyanidin and delphinidin by LEUCOANTHOCYANIDIN DIOXYGENASE (LDOX/TT11/17/18) and to the epi-flavan-3-ols epiafzelechin, epicatechin and epigallocatechin by ANTHOCYANIDIN REDUCTASE (ANR). LEUCOANTHOCYANIDIN REDUCTASE (LAR) catalyses the synthesis of the flavan-3-ols afzelechin, catechin and gallocatechin from leucoanthocyanidins. Modification by methylation is catalysed by O-METHYLTRANSFERASEs (OMTs) e.g. quercetin to isorhamnetin, cyanidin to peonidin and delphinidin to petunidin and malvidin. Multimerization and further modification of epi- and flavan-3-ols results in proanthocyanidins and condensed tannins [9]. Flavonols and (pro)anthocyanidins are the major metabolic sink of the flavonoid biosynthesis pathway [10]. After synthesis, flavonoids - mainly flavonols and anthocyanidins - are subjected to multiple successive modifications through glycosyl-, methyl- and acyltransferases to give rise to a plethora of derivatives [11]. These modifications are necessary for a stable storage of anthocyanidins *in planta* [12].

In particular the analysis of *A. thaliana* mutants has revealed a multi-layered regulation for the key enzymes of the flavonoid biosynthesis pathway [13–15]. Embedded in light signalling, CONSTITUTIVE PHOTOMORPHOGENIC 1 (COP1)/SUPPRESSOR OF PHYTOCHROME A-105 (SPA) complexes mediate posttranslational degradation of light-regulated transcription factors like ELONGATED HYPOCOTYL (HY5) and PRODUCTION OF ANTHOCYANIN PIGMENT 2 (PAP2) which accumulate in *cop1* mutants and transcriptionally activate multiple enzymes of the pathway [16–21]. The COP1 protein is inactivated by light and is therefore mainly active in darkness where it suppresses photomorphogenesis [22, 23]. It is also relevant for the regulation of the circadian clock and photoperiodic flowering [24].

Most knowledge of the regulation of anthocyanidin biosynthesis as part of photomorphogenesis by COP1 and HY5 has been derived from studies on protein stability, genetic analysis of mutants, studies on changes in gene expression in mutants and photometric anthocyanidin measurements.

The expression profile of dark-grown *cop1* mutants is similar to light grown wild-type seedlings explaining the constitutive photomorphogenetic phenotype of these mutants. In *cop1* mutant seedlings, e.g. increased levels of *CHS*, *CHI*, *FLS1* and *F3H* were reported [21, 25]. *CHS* expression serves as one of the markers for COP1-dependent photomorphogenesis. Although COP1 and HY5 act in an antagonistic manner, many *HY5*-regulated genes overlap with the group of *COP1*-regulated genes [21]. HY5 activates the expression of early and late anthocyanidin biosynthesis genes (*CHS*, *CHI*, *F3H*, *F3’H*, *DFR* and *LDOX*) by directly binding to the promoters of these genes in seedlings [16].

*DFR* expression can also be activated by PAP1 and PAP2 [26]. PAP2 can join V-myb myeloblastosis viral oncogene homolog (MYB)/basic-helix-loop-helix (bHLH)/WD40 (MBW) complexes. In the TRANSPARENT TESTA GLABRA 1 (TTG1)-MBW complexes the WD40 protein TTG1 acts together with combinations of different MYB and bHLH proteins to transcriptionally regulate downstream genes [27]. The central role of TTG1 led to a classification of early and late parts of the flavonoid biosynthesis pathway [28] such that late steps are TTG1-MBW-dependent [29–31].

Not only the amount of flavonoids is subjected to regulation but also tissue-specific composition. In *A. thaliana* seeds, mainly epicatechin, proanthocyanidins and quercetin-based glycosides are detected whereas in leaves kaempferol-based glycosides and anthocyanidins dominate [31–34]. Widely differing compositions were reported between species [35].

A central aspect in all studies is the chosen methodology for the analysis of the flavonoid composition. Methods compromise in many respects: the extraction method determines efficiency of substance recovery and modification. The analysis typically compromises on speed, sensitivity and depth of detail for the substances. Due to chemodiversity, the extraction efficiency of substances with differing polarities depends on the solvent [36]. For polar and semipolar substances MeOH/water is used and apolar substances are extracted with chloroform [37]. Using MeOH/water, glycosylated flavonoids are mainly extracted from seeds in the soluble fraction and condensed tannins occur in the non-extracted fractions. Typically, both are subsequently hydrolysed and subjected to photometrical measurement for quantitative comparisons [33]. Ether cleavage is catalysed under acidic conditions combined with heat [38]. The efficiency of hydrolysis is influenced by acidity, temperature and time of hydrolysis. Multiple substances like ferric agent, TFA, butanol, methanol and HCl have been used for hydrolysis of plant extracts [33, 39, 40]. Hydrolytic conditions do not only release aglycons but also cleave the interflavan bonds of proanthocyanidins eventually leading to the release of anthocyanidins from proanthocyanidins [39, 41].

Analysis of natural products has been highly facilitated by improvements of LC-MS detection techniques. Multiple reaction monitoring (MRM), a mode in tandem mass spectrometry, provides high selectivity and sensitivity to lower thresholds of detection. Time-of-flight detectors allow the identification of single metabolites due to precise ion traces [42]. The identity of non-hydrolysed glycosylated flavonoids can be determined through neutral loss analysis employing e.g. pseudoMS^3^ following chromatographic separation [43–46]. For a more precise determination, fragmentation patterns and isolated substances may be additionally analysed by NMR [42]. Meanwhile the combination of metabolomics and transcriptomics has been successfully used for decoding gene functions and to analyse the diversity of the pathway [11, 47–49].

Here we describe a method that aims to facilitate high-throughput studies analysing the flavonoid biosynthesis pathway in a reasonable time frame with sufficient precision and sensitivity to obtain a fingerprint of core components. Towards this end we: 1) provide a robust simple extraction and analysis protocol, 2) established external and deuterated internal standards enabling the unambiguous identification of selected core compounds, 3) reduced the number of biological material (i.e. seeds), and 4) revealed shifts and differences of the pathway in a set of mutants as a proof of principle.

## Results

### Optimizing for high throughput

For optimizing this method for high throughput analysis we considered four aspects: 1. minimizing the time for LC-MS runs and data management, 2. covering a maximum of selected substances at quantifiable levels, 3. minimizing experimental error and 4. reducing the amount of plant material.

The first point is achieved by using the highly selective and sensitive MRM mode in tandem mass spectrometry, which produces data files of small size in combination with a short LC gradient. For quality control purposes two MRMs were selected per reference substance (Additional file 1: Table S1): the quantifier (underlined in Additional file 1: Table S2) and the qualifier (to assure that the correct compound is detected). For separation we used a short column with small particle size based on core-shell technology (KINETEX 2.6 μm C18 100 Å (4.6 mm x 50 mm) C18 column from phenomenex) leading two a high peak resolution at short runtime. The shortest time allowing separation of MRM-peaks for all reference substances was selected. In this study catechin/epicatechin separation was limiting. This setup required only 13 min for LC-MS per sample which corresponds to 80 samples per day including all controls.

Guided by aspects two to four, the remaining parameters were optimized using *A. thaliana* seeds.

### Concept of hydrolysis

In *A. thaliana*, many of the flavonoid molecules are found in various glycosylated forms or are deposited as condensed tannins [11]. As expected, without hydrolysis, we found a complex mixture of compounds resulting in various overlapping peaks using the LC-MS-QTOF set up. Consistent with previous studies we identified predominantly quercetin-based glycosides in seeds and mainly kaempferol-based glycosides in seedlings (Fig. 1c, Additional file 2: Tables S3-S6) [33, 34, 50].

To reduce the complexity of metabolites in seed extracts, we included a hydrolysis step releasing a fingerprint of extracted (e.g. glycosylated flavonoids) and non-extracted (e.g. condensed tannins) substances (Additional file 2: Figure S1). The ether linkage through which modifications are attached to many flavonoids (in most cases containing a glycoside) or the interflavan bond through which multimers are formed (proanthocyanidins) are cleaved under hydrolytic conditions and releases aglycons from glycosylated flavonoids as well as anthocyanidins from proanthocyanidins (Fig. 1b) [11, 33, 39].

### External and internal standards

A set of 11 aglycons and procyanidin B2 was selected as external standards representing different levels of the core pathway (Fig. 1a). These standards cover several levels of the flavonoid biosynthesis pathway in *A. thaliana* from naringenin to kaempferol or taxifolin, either to quercetin and isorhamnetin or to cyanidin and further through epicatechin to a proanthocyanidin like procyanidin B2 [51]*.* In addition we used the flavonol myricetin, the anthocyanidins delphinidin and pelargonidin, and catechin, the epimer of epicatechin (Additional file 1: Table S1, Additional file 3: Figure S2).

In order to analyse extracted and non-extracted hydrolysates, two internal standards were required withstanding the (two-step) extraction procedure. Because we aimed to develop a method adaptable to a wide range of species, we synthesized two substances that are normally not found in plant extracts: D_3_-quercetin and D_3_-sakuranetin (Additional file 3: Methods S1). MRMs were selected (Additional file 1: Table S2) and both standards were tested for linearity (Additional file 3: Figure S3). Concentrations used in this study are in the linear range of the respective internal standard. For D_3_-quercetin, we observed a non-significant decrease to 76 % (+/- 23 %) of its response over time in the presence of acid (1 %FA) relative to non-acidified MeOH (92 % +/- 15 %) when left for 24h at 5°C in the sample taker (Additional file 3: Table S7). Relative to the initial response, the response of D_3_-sakuranetin was neither changed by acid nor over time with a response of 100 % (+/- 23 %) and 95 % (+/- 15 %) with and without 1 %FA.

The external standards were analysed in a range of 1 to 1000 nM and normalized with D_3_-sakuranetin (Additional file 3: Tables S7 and S8). All components were detectable in a linear range with a mean relative standard deviation (RSD) of 14.5 %. Only values for myricetin at a concentration of 1nM were excluded. Comparing the standards in MeOH or MeOH + 1 %FA at 0 and 24h at 5°C (Additional file 3: Table S7) revealed that in acidified MeOH most substances were stable. Therefore, MeOH + 1 %FA was used for the quality control.

### Extraction and hydrolysis time

The selected extraction protocol was modified from an extraction protocol previously used for seeds [33]. We simplified the protocol to allow robust high throughput LC-MS application by extracting with aqueous, acidified MeOH (1 %FA) and by hydrolysis with hydrochloric acid in MeOH instead of BuOH/HCl/ferric agent (e.g. [33, 39]). We added 1 % of formic acid (FA) to stabilize extracted anthocyanidins as they are known to be pH-sensitive [12]. From a wide spectrum of solvents and combinations thereof used for flavonoid extraction [33, 39, 52–54], we decided to use a concentration of 50 % MeOH selected from the optimal window of 30-50 % MeOH. Except for naringenin and myricetin, this solvent allowed the detection of all other substances derived from our set of compounds at quantifiable levels when using ten *A. thaliana* seeds (Additional file 4: Figures S4, S5 and Table S9).

Extracted non-hydrolysed samples contained minor amounts of free core substances (e.g. epicatechin and procyanidin B2), while extracted, hydrolysed samples contained mainly the released aglycons. In non-extracted hydrolysates, released anthocyanidins represent the content of the non-extracted condensed tannins from which they are released [39]. We subjected all three types of extracts to LC-MS analysis and accurate-mass analysis revealed a reduced complexity of hydrolysed samples (Additional file 2: Figure S1).

**Fig. 2:**
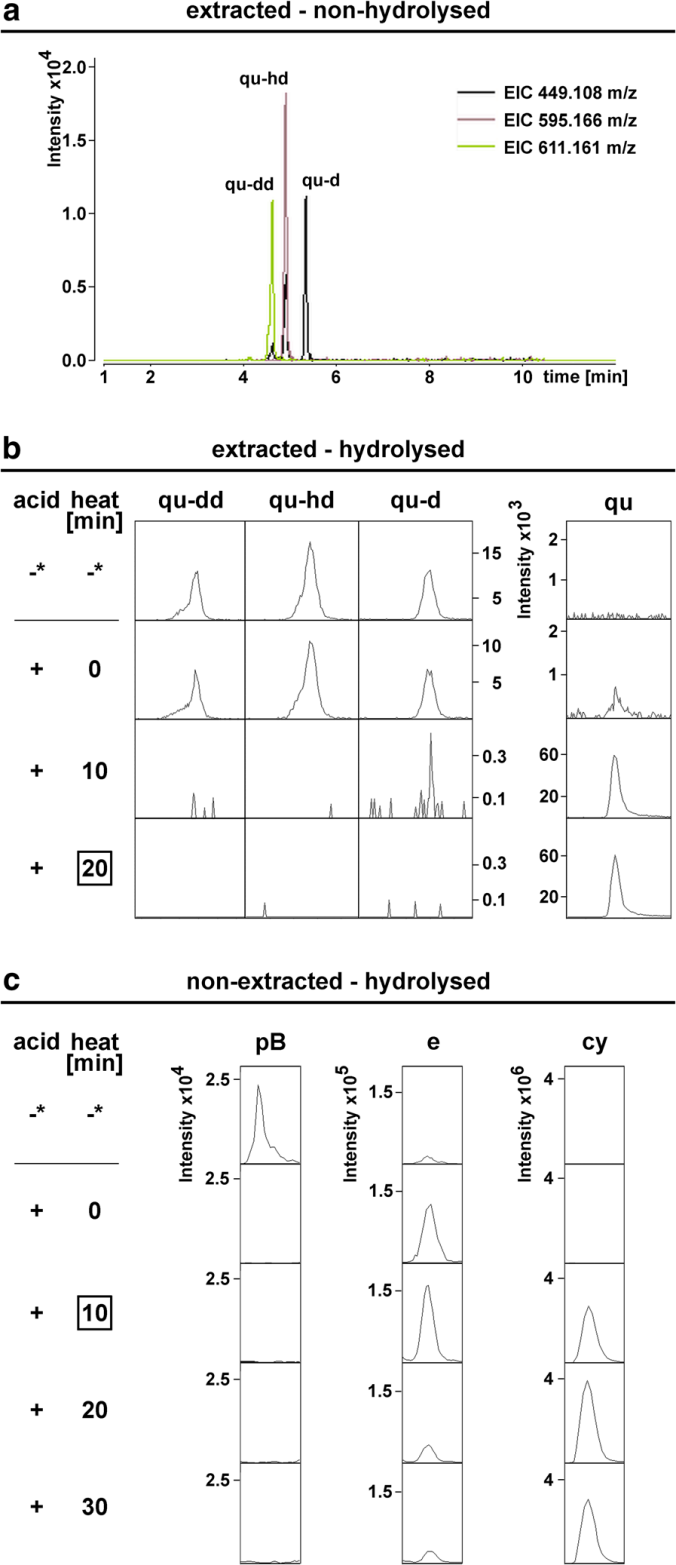
Selection of hydrolysis time for seeds. LC-ESI-MS-QTOF(AutoMSMS) analysis of non-hydrolysed Col-0 seed samples (*n* = 10). **a** Merged EICs for quercetin-bases glycosylated flavonoids with the strongest response. **b** Extracted non-hydrolysed samples before (-) and after addition of acid (+) and heat treatments. **c** non-extracted samples after addition of acid (+) and heat treatments. *: extracted non-hydrolysed samples are shown for comparison. acid: HCl in MeOH. Boxed: selected hydrolysis time derived from this experiment. See Additional file 4: Figure S6 and Table S10

In previous studies, a hydrolysis time of 60 min was used [33, 52]. In the next step, we aimed to reduce this hydrolysis time to minimize the degradation of core substances while completely hydrolysing the most abundant glycosylated flavonoids. Three quercetin based glycosides dominate extracted non-hydrolysed samples in LC-ESI-MS-QTOF(pseudoMS^3^) analysis (Fig. 2a). After twenty minutes of hydrolysis, none of these were detectable anymore (Fig. 2b). Longer hydrolysis led to reduced epicatechin levels (Additional file 4: Figure S6 and Table S10). Therefore, we used a hydrolysis time of 20 min for the extractable fraction. For the non-extractable fraction, a shorter application of hydrolytic conditions of 10 min proved to be optimal (Fig. 2c, Additional file 4: Figure S6).

### Effect of hydrolysis on reference substances

In hydrolysed samples, the method described in this study aims to compare whole branches of the pathway between mutants or ecotypes to reveal major shifts. This facilitates the identification of parts of the pathway that can be analysed in more detail. Therefore, the method uses core substances of the flavonoid pathway analysed in hydrolysed samples as representatives for groups of substances, namely all substances from which they can be released by hydrolysis.

We tested for each of the selected substances the corresponding external standards separately through hydrolysis for possible degradation or conversions. *tt4-11* seeds were spiked with the respective substance prior to extraction (Additional file 5: Figure S7). For comparison, the respective standards were diluted with the same factor as introduced through extraction and treatment using MeOH +/-FA (according to the respective stability, see Additional file 3: Table S7).

All substances - except procyanidin B2 - withstand both hydrolysis protocols. The procyanidin B2 standard (90 % purity) releases epicatechin and cyanidin at quantifiable levels. In hydrolysed samples, no procyanidin B2 is detectable. The release of cyanidin from procyanidin under hydrolytic conditions has been widely used before to estimate procyanidin levels [39]. Despite a conversion from procyanidin B2, epicatechin might also be released from impurities in the standard.

**Fig. 3:**
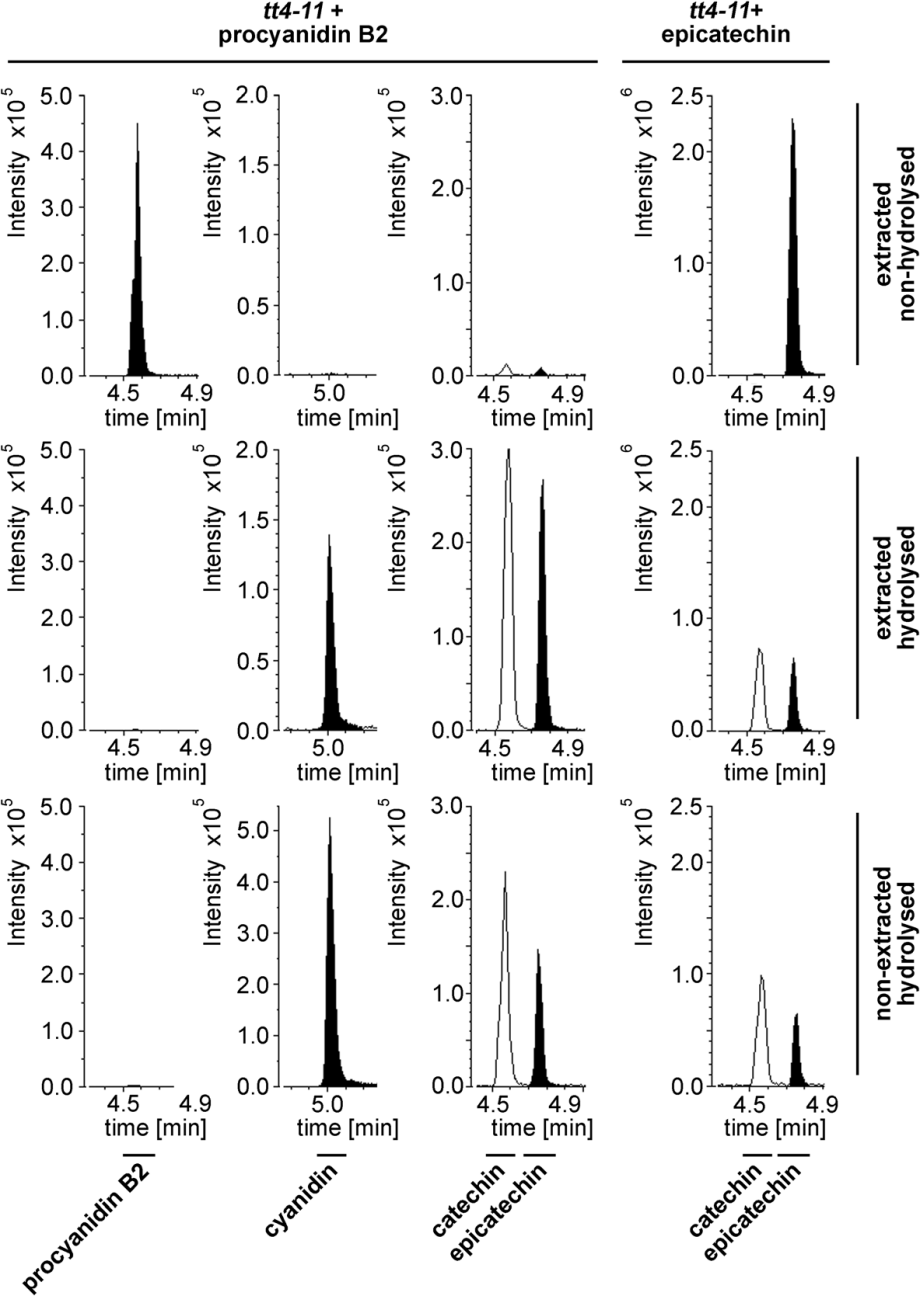
Epimerization of epicatechin to catechin. Analysis of the conversion of procyanidin B2 and epicatechin after different treatments by LC-ESI-MS-QTRAP(MRM). The above indicated substances were spiked on ten *tt4-11* seeds prior to extraction (extracted samples) or prior to hydrolysis (non-extracted samples). Shown are MRMs for the substances indicated below the chromatograms

Furthermore, we found that epicatechin can be converted into catechin (Fig. 3, *tt4-11* + epicatechin). Catechin is not detectable in non-hydrolysed Col-0 seed samples but found in hydrolysed samples indicating that epiconversion occurs during hydrolysis. Therefore, no conclusions on the presence of catechin can be drawn from hydrolysed samples. Surprisingly, myricetin, pelargonidin and delphinidin released quantifiable amounts of catechin but not epicatechin under hydrolytic conditions for extracted samples. One possible explanation for this observation could be impurities in the standard.

Pelargonidin was detectable from all extracted hydrolysed samples including the sample only containing the internal standard (Additional file 5: Figures S7, S8a-c). No detectable release of pelargonidin was observed from non-extracted hydrolysed samples. This indicates that low amounts of pelargonidin are released from D_3_-quercetin under hydrolytic conditions in the extracted samples. Therefore, minor levels of pelargonidin are not considered when interpreting results from extracted and subsequently hydrolysed samples.

In addition to these results, we cannot exclude that compounds generated downstream of CHS/TT4 which are not represented in our set of standards release one of our selected core substances under hydrolytic conditions from seeds with an intact CHS/TT4 gene.

### Minimizing the number of seeds

Previous studies and specific extraction protocols require up to 200 seeds or even more [33, 52]. However, seed material is often limiting and genetic analysis may require the analysis of a particular genotype in many replicates. We therefore aimed to adopt the method to a minimum number of *A. thaliana* Col-0 seeds.

**Fig. 4:**
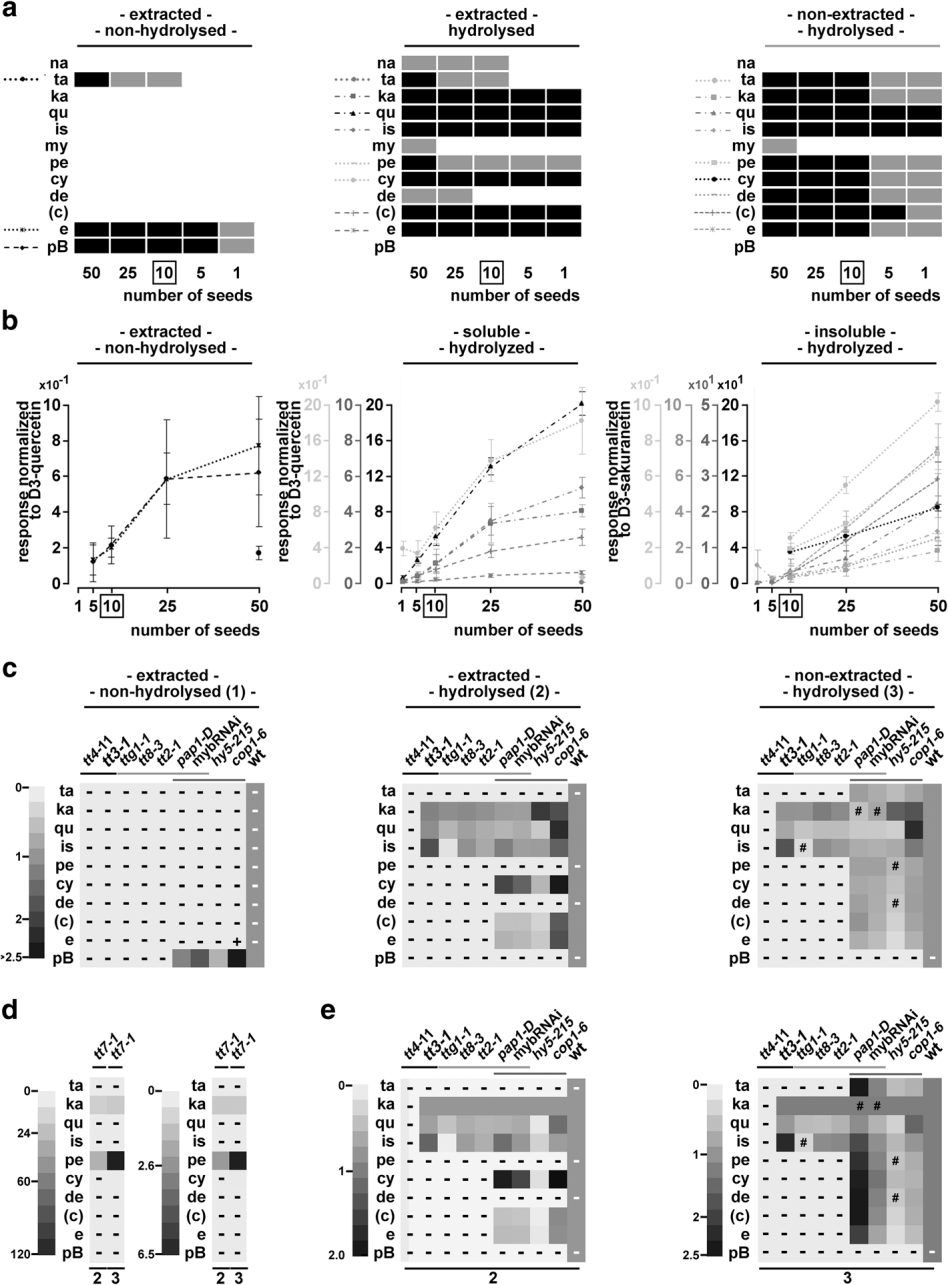
Reduction of seed material and proof of principle with seeds. Reduction of seed material down to one seed (**a**,**b**) and application to a set of selected mutants (**c**-**e**) with extracted non-hydrolysed, extracted hydrolysed and non-extracted hydrolysed samples analysed by LC-ESI-MS-QTRAP(MRM). **a** Flavonoids with detectable and quantifiable responses in 50, 25, 10, 5 and 1 *A. thaliana* Col-0 seed(s). Grey: LOD but not LOQ is passed, black: LOQ is passed for the majority of replicates. See also Additional file 6: Figure S9 and Additional file 5: Figure S7 and S8 (effect of hydrolysis on analysed flavonoids). **b** Responses normalized with the respective deuterated internal standard. Several scales are used for each subfigure. Grey values of scales correspond to the grey values of the substances. Boxed: optimal seed number according to this experiment. Error bars = STDEV. (Additional file 6: Table S11: statistics) **c**-**e**) Mutant analysis: Heatmaps showing log_2_ of fold differences relative to the respective wild types. **d** Same as in (**c**) and (**e**) for *tt7-1* with appropriate scales. **e** Same data as in (**c**) but normalized with kaempferol. -: LOD not passed, #: LOD but not LOQ passed, +: LOQ passed for the mutant but not for the wild type. Lines in grey shades group the three sets of mutants: enzymes, TTG1-MBW complex components, light signalling mutants. Additional file 7: Tables S12 and S13: statistics. Additional file 7: Figure S10: responses normalized with respective deuterated internal standard. **c** Detected catechin which is most likely derived from epimerization of epicatechin (see Fig. 3)

With one seed, we were able to detect in at least three of five samples kaempferol, quercetin, isorhamnetin, cyanidin and epicatechin/catechin at quantifiable levels (>LOQ) which represent the most abundant released flavonoids from seeds (Fig. 4a, Additional file 6: Table S11). In the experiment shown in Fig. 4a, few substances were close to their respective threshold of quantification for specific seed numbers. In a replicate experiment, for example, taxifolin was quantifiable in five seeds in contrast to kaempferol (Additional file 6: Figure S9). Although detectable at levels passing the LOQ in extracted hydrolysed samples from one seed, the RSD ranged between 20 to 50 % for the above named six substances (Additional file 6: Table S11). Over all, most substances were detectable at quantifiable levels in the linear range when analysing the extracted fraction from five to 25 seeds (Fig. 4b, Additional file 6: Table S11). To achieve this with the non-extracted fraction, ten to 50 seeds could be used. Consequently, we used ten seeds for all subsequent experiments.

Pelargonidin was detectable but not quantifiable in the whole range from one to 25 seeds in extracted hydrolysed samples which is explained by its release from D_3_-quercetin.

Unexpectedly, we detected delphinidin in hydrolysed samples. This is surprising as no F3’5’H gene is present in *A. thaliana* and the detection of delphinidin has not been reported for *A. thaliana*. One possible explanation is the conversion from flavonoids not covered by our reference set. Alternatively, it is possible that delphinidin is released from derivatives in extracted and non-extracted fractions. In this context, it might be relevant that delphinidin is fairly unstable over time, decreases with increased hydrolysis time (Additional file 3: Table S7, Additional file 4: Figure S6 and Table S10) and that our reduced hydrolysis time enables the delphinidin detection.

### Proof of principle: revealing shifts in the pathway

As a proof of principle for revealed metabolic shifts and differences in the pathway, we applied the method to a set of mutants affected in different steps of the flavonoid pathway.

Two types of mutants with different effects on the pathway were analysed: First, defective or absent enzymes completely block whole branches or the entire pathway. Second, defective transcriptional regulators affect one or multiple enzymes of the pathway. We selected mutants defective in the enzymes CHS, F3’H and DFR (*tt4-11*, *tt7-1* and *tt3-1*), mutants in transcriptional regulators of the pathway including TTG1, TT8 and TT2 (that block the same branch of the pathway as mutants of *DFR/TT3*) and light signalling mutants of *COP1*, *HY5*, a *PAP1* overexpressor line and a RNAi line repressing *PAP1* through *4* (*PAP1, PAP2, MYB113 (PAP3), MYB114 (PAP4)* [28, 55].

Shifts and blockages within the pathway were observed in all three selected *A. thaliana* mutants affecting enzymes (*tt4-11*, *tt7-1* and *tt3-1*) (Fig. 4c,d, Additional file 7: Figure S10), which is in agreement with previous studies [33, 56]. In *tt7-1* the observed shift to the kaempferol/pelargonidin branch was reported before and is clearly visualized (Fig. 4d) [33]. To our surprise we also detected cyanidin at quantifiable levels in *tt7-1* extracts after hydrolysis. A close analysis of chromatograms, however, revealed that hydrolysis releases small amounts of cyanidin from the pelargonidin reference substance (Additional file 5: Figure S8d,e). Thus, quantitative analysis of hydrolysed extracts with high pelargonidin level requires the adjustment of thresholds for cyanidin quantification.

The MBW complex components TTG1, TT8 and TT2 are known to be essential regulators of *DFR* [27, 57]. In agreement with this, no products of late enzymes were detected in the three respective mutants as described before (e.g. [33]). The block at DFR in *tt3-1* mutants led to a strong increase of isorhamnetin and slightly elevated kaempferol levels while quercetin levels were similar relative to wild type. In contrast to *tt3-1*, the three MBW mutants *ttg1-1*, *tt8-3* and *tt2-1* did not exhibit elevated kaempferol levels and all had reduced quercetin levels. Most strikingly, isorhamnetin levels were severely reduced in *ttg1-1* (Fig. 4c, Additional file 7: Figure S10, Tables S12 and S13) but not in *tt8-3* and *tt2-1*. This was not observed in *tt8-3* and *tt2-1* and therefore points to an independent role of *TTG1* in regulating isorhamnetin.

In addition to TT2, four R2R3 MYB factors, PAP1 through 4, are known to regulate flavonoid biosynthesis together with TTG1. Overexpression of PAP1 in the activation tagging line *pap1-D* led to an accumulation of extracted hydrolysable cyanidin derivatives at the expense of quercetin, epicatechin and their respective derivatives (Fig. 4c,e). Non-extracted but hydrolysable derivatives of kaempferol, quercetin, cyanidin and epicatechin were significantly reduced in *pap1-D* seeds. None of the substances from our set was increased in the non-extracted hydrolysed samples. This suggests that less flavonoids were deposited in non-extracted substances as compared to the wild type. Possibly, PAP1 primarily regulates cyanidin modifying enzymes in seeds and thereby, when overexpressed, creates a sink situation affecting the seed’s flavonoid composition.

The flavonoid composition of seeds from the *myb*RNAi line was similar to wild type. Few substances were significantly reduced as compared to wild type like quercetin and epicatechin. The latter could be explained by the transcriptional regulation of *ANR* by *PAP*(s). This view is supported by the previous finding indicating that PAP4 overexpression in *A. thaliana* mesophyll protoplasts activates *ANR* [55]. Non-normalized and normalized results point to a slight downregulation of the pathway as no substance stood out when set relative to kaempferol. The results for *pap1-D* and the *myb*RNAi line suggest the (partially) redundant regulation of the flavonoid pathway.

In seedlings, HY5 is known to activate the expression of early and late biosynthesis genes by directly binding to the promoters of these genes [16]. *hy5-215* seeds revealed various changes of the flavonoid composition In hydrolysed samples, kaempferol was significantly increased and most other flavonoids are reduced (Fig. 4c,e). This suggests a role of HY5 in the regulation of *TT7* or downstream genes.

COP1 is predicted to affect the level of most substances in the pathway because it regulates the stability of relevant transcription factors like HY5 and PAP2 [19, 58]. As expected, the spectrum of extracted hydrolysable flavonoids accumulating in *cop1-6* partially overlapped with that in *pap1-D*. In *cop1-6* seeds, levels of cyanidin derivatives were high but not at the expense of other substances downstream of kaempferol as seen by normalization with kaempferol. In addition, we found several unexpected aspects of COP1 regulatory events influencing the flavonoid composition and deposition in seeds.

First, we revealed a role of *COP1* in suppressing the accumulation of extracted quercetin- and isorhamnetin-based substances. One possibility is the mis-regulation of core enzymes in *cop1-6*. Alternatively, COP1 could suppress at least one quercetin and isorhamnetin modifying enzyme. Second, while high levels of cyanidin are present in the extracted hydrolysed samples, epicatechin levels remain unchanged as compared to wild type. A suppression of enzymes downstream of cyanidin and epicatechin is therefore unlikely. This points to a positive regulation of enzymes downstream of cyanidin which do not effect levels of epicatechin and downstream substances like cyanidin modifying (e.g. glycosylating) enzymes. Third, in contrast to an expected enhanced deposition of non-extracted condensed tannins or the extracted non-hydrolysed procyanidin B2 in *cop1-6* seeds, we detected a significant decrease of substances released from the non-extracted fraction (taxifolin, pelargonidin, cyanidin and epicatechin).

### Adaptation of the method to seedlings

Many studies analysing flavonoids have been conducted using seedlings. Protocols have been developed for specific needs ranging from photometric analysis to different types of metabolomics and other specific applications [37, 59, 60]. Here, we aimed to obtain fingerprints for shift detection from seedlings at high throughput. We used the extracted hydrolysis setup to adapt our method. Hydrolysed extracts from seedlings were subjected to LC-ESI-MS-QTRAP(MRM) to select the optimal number of seedlings for screening purposes. Furthermore, we applied the experimental setup to selected mutants based on results from seeds.

**Fig. 5:**
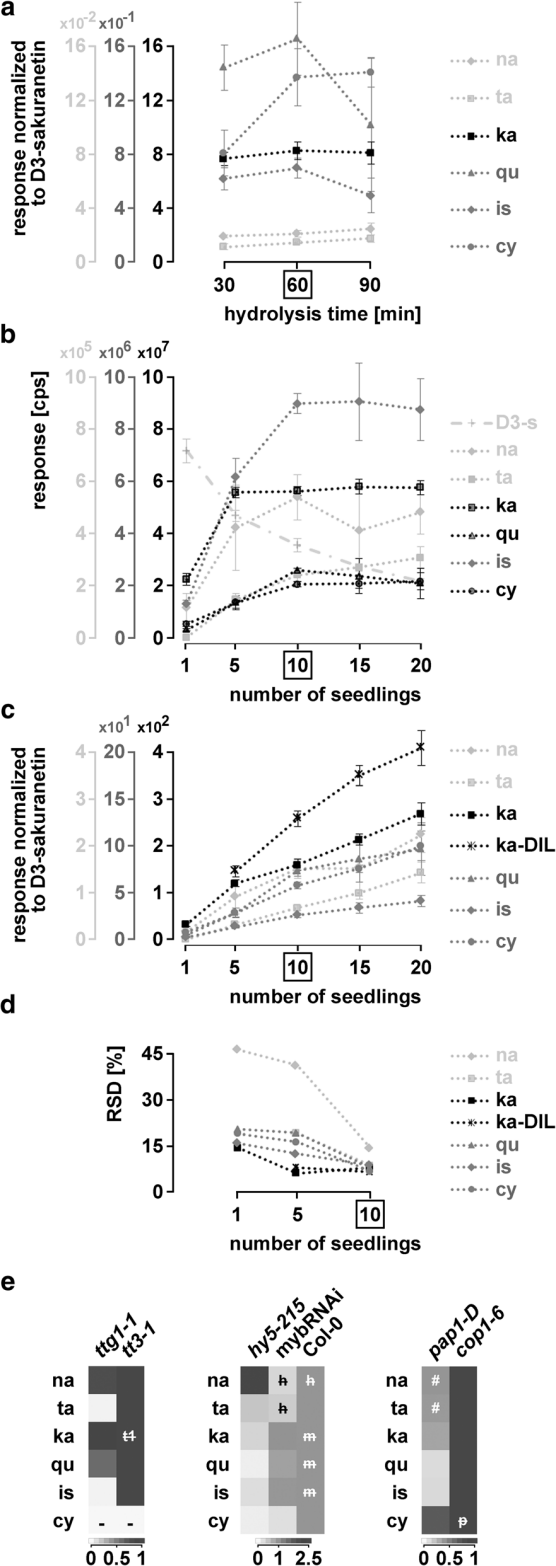
Adaptation of the method to seedlings. Analysis of seedlings by LC-ESI-MS-QTRAP(MRM). **a** D_3_-sakuranetin-normalized responses of flavonoids tested for different times of hydrolysis. **b**-**d** Selection of the optimal number of seedlings for the set of analysed flavonoids. **b** Responses of the different flavonoids tested, including D_3_-sakuranetin, for 1, 5, 10, 15 and 20 seedlings. cps: counts per second. **c** D_3_-sakuranetin-normalized responses from (**b**) and diluted samples for kaempferol. Please note that only quantifiable amounts are shown (Additional file 8: Table S14) **d**) RSD in percent for 1, 5 and 10 seedlings from (**c**). **e** Mutants. Heatmaps showing log_2_ transformed D_3_-sakuranetin-normalized responses (*n* = 3) relative to *tt3-1*, Col-0 and *cop1-6*. Statistics and mean normalized responses: Additional file 8: Table S14. Boxed: optimal number of seedlings according to these experiments. Error bars = STDEV. # LOD but not LOQ passed. -: LOD not passed. h, m, p and t1: no significant difference to the respective mutant. h: *hy5-215*, m: *myb*RNAi, p: *pap1-D*, t1: *ttg1-1*. ka-DIL: kaempferol-diluted

Initially, a hydrolysis time of 60 min was employed as used in other studies [33, 52, 53]. This hydrolysis time proofed to be optimal, as none of the detected, quantifiable substances significantly increased when using 30 or 90 min (Fig. 5a, Additional file 8: Table S14). In addition, the major glycosylated flavonoids were absent after 60 min of hydrolysis (Additional file 8: Figure S11).

Detected responses for several substances approached a maximum at ten seedlings which turned out to be sufficient to detect most substances (Fig. 5b). Kaempferol responses were high and reached the maximum at five seedlings already. This required the analysis of diluted samples from five seedlings onwards for kaempferol detection (Fig. 5c). All substances found when using ten seedlings (taxifolin, naringenin, kaempferol, quercetin, isorhamnetin and cyanidin) were quantifiable in five and one seedling except for taxifolin which did not reach the limit of quantification in one seedling. In general, RSDs were reduced by the internal standard (Fig. 5b,c). Because ten seedlings are required to reach an RSD below 20 % for all substances at quantifiable levels (Fig. 5d), we therefore recommend the use of ten seedlings.

Unexpected changes in the pathway which were revealed by fingerprinting of mutant seeds prompted us to analyse *ttg1-1* seedlings and seedlings of light signalling mutants.

When comparing *ttg1-1* to *tt3-1* mutants, we again found evidence for a role of *TTG1* in regulating *TT7* since taxifolin levels were reduced in *ttg1-1* when compared to *tt3-1*. Relative to *tt3-1*, isorhamnetin levels were drastically reduced and quercetin levels were slightly reduced in *ttg1-1* mutants. This indicates that *TTG1* regulates the accumulation of isorhamnetin in both, seeds and seedlings.

Differences in the flavonoid composition between seeds and seedlings were also found in *myb*RNAi lines. In hydrolysed extracts from *myb*RNAi seedlings, naringenin, taxifolin and cyanidin were significantly reduced while the flavonols were not affected. This suggests that *PAP* genes regulate steps upstream of naringenin synthesis (e.g. *CHS* and *CHI*). An additional regulation of *DFR* and/or *ANS* is suggested by the finding that cyanidin levels were reduced, whereas quercetin levels remained unchanged. This is consistent with the previous finding that *DFR* expression can be activated by PAP1 and PAP2 [26]. An alternative explanation is the regulation of enzymes modifying flavonoids along the naringenin-taxifolin-cyanidin-branch.

In *hy5-215*, we found reduced levels of all substances downstream of naringenin. As kaempferol levels were increased in seeds, it is conceivable that *HY5* acts at different steps in the flavonoid pathway in seeds and seedlings.

The extracts of *pap1-D* and *cop1-6* had to be diluted 20-fold to reach the linear range of the set of flavonoids. All D_3_-sakuranetin-normalized responses for *cop1-6* in the dilutions were beyond the undiluted, normalized wild-type response. For *pap1-D*, similar as in seeds, cyanidin responses showed the strongest increase compared to the other detected substances and even reached similar levels as in *cop1-6* mutants.

The levels of all analysed substances upstream of cyanidin were lower in *pap1-D* compared to those in *cop1-6*. We therefore conclude that *COP1* is a master regulator of the flavonoid biosynthesis pathway in seedlings but not of flavonoid deposition in seeds.

## Discussion

The qualitative and quantitative analysis of flavonoids in plants is well established. Photometric methods enable a quick estimation of anthocyanidin contents [33, 60] or, in combination with LC and UV detectors, an assessment of their composition (e.g. [33, 61]). A more detailed analysis is done by LC-MS using e.g. MRM, TOF detectors, MSMS or pseudoMS^3^ [42–46]. Depending on the specific questions either the extracted fractions or the non-extracted hydrolysed fraction are used. Typically, methods compromise in many respects to adapt for the specific goal. The method established here, is optimized for situations in which the whole flavonoid pathway needs to be quantitatively monitored in many samples and with limited biological material. Three aspects were relevant for the optimization. First, we determined the shortest hydrolysis time by considering two criteria: the complete removal of glycosylated modifications and the minimal conversions of flavonoids into others. The latter is important to avoid false signals leading to a misinterpretation of alterations in the pathway. Second, the method is suitable to detect most key flavonoids in only one seed or seedling. Using this fingerprint, it is possible to compare alterations in the flavonoid pathway in many biological samples. Third, we combine three types of samples, the extracted non-hydrolysed, the extracted hydrolysed and the non-extracted hydrolysed samples. This combination proofed to be very useful to decide whether individual flavonoids have been released from extracted or non-extracted derivatives. In our study it was particularly helpful to recognize a role of COP1 in the regulation of enzymes modifying quercetin, isorhamnetin and cyanidin.

Our analysis of the flavonoid pathway in mutants revealed several interesting new findings. It is well accepted that *DFR* is regulated by TTG1, the bHLH protein TT8 and the R2R3MYB protein TT2 possibly in a TTG1-MBW complex [27, 57]. One would therefore expect that *ttg1-1* mutants show a similar flavonoid composition and levels as *tt3-1*, *tt8-3* and *tt2-1* mutants. This is clearly not the case. On the one hand, *ttg1-1* mutants do not exhibit the expected increase in isorhamnetin levels but rather a reduction. On the other hand, *tt8-3* and *tt2-1* mutants differ from *ttg1-1* as they show wild-type levels of isorhamnetin. Moreover, *ttg1-1*, *tt8-3* and *tt2-1* exhibit decreased quercetin levels which is not seen in *tt3-1*. These data suggest two regulatory features of TTG1. First, the decreased quercetin levels suggest that TTG1, TT8 and TT2 are involved in earlier steps of the pathway or quercetin modification. One likely explanation is its regulation of *TT7* as reported before [28]. The different compositions in *tt8-3* and *tt2-1* mutants indicate that TTG1 also exerts this function independently of TT8 and TT2. Possible targets are *OMT* or isorhamnetin modifying enzymes.

The role of *TTG1* in regulating isorhamnetin is also found in seedlings suggesting that *TTG1* regulates *OMT* or the isorhamnetin modifying enzyme in both tissues in the same manner. By contrast we found a tissue-specific regulation of the flavonoid pathway in light signalling mutants, in particular by *COP1*.

While *COP1* has little impact on the flavonoid pathway in seeds, it is the master regulator in seedlings. This is indicated by the findings that for all detected substances in seedlings, *cop1-6* mutants released the highest levels.

Differential regulation of the flavonoid pathway in seeds and seedlings were also observed in mutants of transcription factors downstream of COP1. Similar to *cop1-6* mutants, *pap1-D* seedlings exhibited highly increased cyanidin levels relative to the other flavonoids. In *myb*RNAi lines naringenin, taxifolin and cyanidin levels are reduced in seedlings but not in seeds. Interestingly, *HY5* is equally important in seeds and seedlings but acts at different levels of the pathway. In seeds, kaempferol accumulates in the *hy5-215* mutant but is reduced in seedlings, which suggests that *HY5* regulates steps upstream of *TT7* in seedlings, whereas in seeds, TT7 or downstream enzymes are controlled. Together, our data support a dependence on the developmental stage for COP1 function: while the flavonoid composition of seeds is only moderately dependent on the COP1-dependent light signalling pathway, COP1 is a master regulator of the pathway in seedlings.

## Conclusions

In this work, we developed a method for the analysis of the flavonoid biosynthesis pathway optimized for purposes for which quantitative comparison with limited plant material is important. In proof of principle experiments the method revealed a differential regulation of kaempferol by HY5 in seedlings versus seeds and provided evidence for the role of COP1 as a master regulator of the pathway in seedlings but not for flavonoid deposition in seeds. This method will be helpful in genetic studies and in particular for the analysis of natural variation in different ecotypes or genetic mutants.

## Methods

### Plant material and growth condition

All mutants used in this study have been described before [7, 28, 57, 62–73]. The following mutants can be obtained from the Nottingham Arabidopsis Stock Centre (NASC): *tt2-1* (N83), *tt3-1* (N84), *tt4-11* (N2105573), *tt7-1* (N88), *tt8-3* (N891), *pap1-D* (N3884), *cop1-6* (N69041). Mutants and primers for genotyping are listed in Additional file 9: Tables S15 and S16. For designing dCAPS primers, dCAPSfinder 2.0 was used [74].

All *A. thaliana* ripened seeds originated from plants grown at 21°C in 16/8 h light (90-120 μmol*m^-2^s^-1^, LUMILUX Cool white L58W/840, Osram, http://www.osram.de). For seedling analysis, seeds were sown on solid MS plates (1 % sucrose), kept in darkness at 4°C for 2.5 days prior to transfer to white light (4d, 21°C, 40 μmol*m^-2^s^-1^, FLUORA L58W/77, Osram, http://www.osram.de).

### Chemicals, external standards

MeOH (CHROMASOLV, Fluka, http://www.sigmaaldrich.com), formic acid (FA) and HCl conc. (both NORMAPUR, AnalaR, www.vwr.com) were used at LC-MS grade. The external standards (Sigma-Aldrich (https://www.sigmaaldrich.com), Roth (https://www.carlroth.com)) are listed in Additional file 1: Table S1. For quality control of LC-MS analysis all 12 external standards were mixed (100 nM) with 400 nM D_3_-quercetin and 5 nM D_3_-sakuranetin. (For experiments with external standards: see Additional file 9: Methods S2.)

### Internal standards synthesis

To synthesize D_3_-Quercetin, we followed a modified three-step procedure using a BF_3_^.^THF solution instead of BF_3_ gas [75, 76]. The enrichment was analysed by ESI-MS using a Thermo Exactive Orbitrap MS (Thermo Fisher, http://www.thermoscientific.com). D_3_-sakuranetin was synthesised by methylation of naringenin as shown before [77] using deuterated methyl iodide under basic conditions. All methods for internal standard synthesis are documented in Additional file 3: Methods S1.

### Flavonoid extraction

Protocols are described as derived after optimizing the MeOH concentration for seeds, seed(ling) number and hydrolysis time for seed(ling)s. In all experiments, quintuplicates are analysed if not stated otherwise.

Ten seeds were homogenized in 100 μl MeOH:H_2_O:FA (50:49:1), spiked with 10 μl D_3_-quercetin (100 μM) using a TISSUELYSER (Qiagen, https://www.qiagen.com) with three glass beads (BIOSPEC, #11079125/N032.1, Roth, www.carlroth.com) for 3 min at 30 Hz. Upon centrifugation (14000 rpm, 5 min, 4 °C), the pellet was re-extracted in 200 μl of extraction buffer (4°C over-night) followed by centrifugation. 60 μl of combined supernatants were mixed with 440 μl MeOH (extracted non-hydrolysed sample). 120 μl of the supernatants and 380 μl MeOH:HClconc. (95:5) were hydrolysed (99 °C, 20 min) and diluted 1:1 with D_3_-sakuranetin(200 nM):MeOH (1:20) (extracted hydrolysed sample). D_3_-sakuranetin served in this sample to monitor fluctuation of the machine. For non-extracted proanthocyanidins, the pellet was hydrolysed (99°C, 10 min) with 210μl of D_3_-sakuranetin(200nM):MeOH:HClconc (1:19:1). Supernatants were diluted 1:1 with MeOH following two rounds of centrifugation (non-extracted hydrolysed sample).

10 N_2_liq-frozen seedlings with 210μl of D_3_-sakuranetin(210nM):MeOH (1:20) were homogenized as above but with 22Hz, incubated in darkness (4°C overnight) and centrifuged (14000rpm, 12min). 100 μl of the supernatant were hydrolysed (99 °C, 60 min), centrifuged and 60 μl supernatant were diluted 1:1 with MeOH. For 10 Col-0 and mutant seedlings, these extracts were further diluted 1:6 with MeOH to analyse kaempferol in the linear range (Fig. 5 c,d).

### LC-MS

A KINETEX 2.6 μm C18 100 Å (4.6 mm x 50 mm) C18 column (Phenomenex, http://www.phenomenex.com) was used for all samples. For quantification via LC-MS/MS in MRM mode (=LC-ESI-MS-QTRAP(MRM)) an Agilent 1260 HPLC (http://www.agilent.com/home) coupled to a QTRAP 5500 mass spectrometer (ABSCIEX, http://sciex.com) were used; for identification (AutoMSMS and pseudoMS^3^ experiments), a DIONEX 3000 RSL UPLC (Thermo Fisher, http://www.dionex.com) system was coupled to a MAXIS 4G (Bruker Daltonic, www.bruker.com) (=LC-ESI-MS-QTOF(AutoMSMS or pseudoMS^3^)). For details on LC-MS see Additional file 9: Methods S2.

### Detection of glycosylated flavonoids and identification of their aglycons

Extracted non-hydrolysed samples were injected twice and subjected once to LC-ESI-MS-QTOF(AutoMSMS) and once to LC-ESI-MS-QTOF(pseudoMS^3^). The Auto MSMS run revealed glycosylated flavonoids, the pseudoMS^3^ run identified the specific flavonoid via MS/MS fragmentation pattern as compared to the respective external standards. Both contribute through neutral loss analysis to identify the type of glycosylation (here: hexose or deoxy-hexose, for further details and reference *m/z* values see Additional file 2: Tables S3-S6).

### General data analysis, statistics, and specific visualization

Using non-smoothed data, the limit of detection (LOD) was defined at a signal-to-noise ratio (S/N) of 3 and the limit of quantification (LOQ) at a S/N of 10. LOD or LOQ was assigned for a mean when more than half of the replicates passed the respective threshold. All raw LC-ESI-MS-QTRAP data were analysed using Analyst 1.6 (ABSCIEX, http://sciex.com). Integrated peak areas from the inbuilt Analyst Quantitation Wizard were manually corrected. LC-ESI-MS-QTOF raw data were mass corrected with sodium formate clusters using HPC with Compass AutomationEngine 4.1. Subsequent analysis was performed with Compass DataAnalysis Version 4.1 SP5.

Statistic analysis was done using R 3.0.2 (Shapiro test, Welch test, Wilcoxon rank sum test) [78–81]. Heatmaps were generated for log_10_ transformed values with heatmap.2 in R 3.0.1 using gplots and RColorBrewer [82, 83]. All other heatmaps were generated with the BAR HeatMapper Plus Tool (http://bar.utoronto.ca/ntools/cgi-bin/ntools_heatmapper_plus.cgi) and changes to greyscale.

## Additional files

Additional file 1: Table S1. Optimizing for high throughput. List of external standards; and Table S2. Transition parameters. (PDF 381 kb) Additional file 2: Figure S1. Concept of hydrolysis. Reduced complexity of extracts after hydrolysis; Table S3. Calculated m/z values for detection of glycosylated flavonoids; Table S4. Glycosylated flavonoids in extracted non-hydrolysed samples from *A. thaliana* seeds; Table S5. Glycosylated flavonoids in extracted non-hydrolysed samples from *A. thaliana* seedlings; and Table S6. Calculated m/z values for selected substances. (PDF 1704 kb) Additional file 3: Figure S2. External and Internal standards. EICs of external standards; Figure S3. Linearity and EICs of deuterated, internal standards; Table S7. Stability of standards over time and in dependence of acid, Table S8. Precision and linearity of the selected external standards; and Methods S1. Internal standard synthesis. (PDF 1682 kb) Additional file 4: Figure S4. Extraction and hydrolysis time. Selection of the methanol concentration; Figure S5. Non-adjusted responses from Figure S4; Figure S6. Seed extracts treated with different hydrolysis times; Table S9. Data for Figures S4, S5; and Table S10. Data for Fig. 2 and Figure S6. (PDF 1210 kb) Additional file 5: Figure S7. Effect of hydrolysis on reference substances. Effect of hydrolysis on analysed flavonoids; and Figure S8. Release of pelargonidin from the internal standard and cyanidin from pelargonidin. (PDF 493 kb) Additional file 6: Figure S9. Minimizing the number of seeds. Reduction of seed material down to one seed – repetition; and Table S11. Data for Fig. 4a,b. (PDF 356 kb) Additional file 7: Figure S10. Proof of principle: revealing shifts in the pathway. Application (seeds of mutants) – normalized responses; Table S12. Data for Fig. 4c-e.; and Table S13. Data for Fig. 4c,d – significance. (PDF 731 kb) Additional file 8: Figure S11. Adaptation of the method to seedlings. Hydrolysis of main glycosylated flavonoids from seedling extracts; and Table S14. Data for Fig. 6. (PDF 445 kb) Additional file 9: Table S15. Mutants and primers. Mutants and insertion lines used in this study; Table S16. Primer; and Methods S2. (PDF 431 kb)

## Abbreviations

(e-)f3-ol: (epi-)flavan-3-ol
ANR: Anthocyanidin reductase
ANS: Anthocyanidin synthase
BF3: Boron trifluoride
bHLH: Basic-helix-loop-helix
BuOH: Butanol
c: Catechin
CHI: Chalcone synthase
CHS: Chalcone syntase
COP1: Constitutive photomorphogenic 1
cps: Counts per second
cy: Cyanidin
d: Deoxy-hexose
D3-q: D3-quercetin
D3-s: D3-sakuranetin
de: Delphinidin
DFR: Dihydroflavonol 4-reductase
e: Epicatechin
EIC: Extracted ion chromatogram
ESI: Electrospray ionization
F3’5’H: Flavonoid 3’,5’ hydroxylase
F3’H: Flavonoid 3'-monooxygenase
F3H: Flavanone 3-hydroxylase
FA: Formic acid
FLS: Flavonol synthase
GST: Glutathione S-transferase
h: Hexose
HCl: Hydrochloric acid
HPC: High precision calibration
HY5: Elongated hypocotyl 5
is: Isorhamnetin
ka: Kaempferol
ka-DIL: Kaempferol-diluted
LAR: Leucoanthocyanidin reductase
LC: Liquid chromatography
LDOX: Leucoanthocyanidin dioxygenase
LOD: Limit of detection
LOQ: Limit of quantification
MBW: MYB/bHLH/WD40
MeOH: Methanol
MRM: Multiple reaction monitoring
MS: Mass spectrometry
my: Myricetin
MYB: V-myb myeloblastosis viral oncogene homolog
na: Naringenin
NMR: Nuclear magnetic resonance spectroscopy
OMT: O-methyltransferase
PAP: Production of anthocyanin pigment
pB: Procyanidin B2
pe: Pelargonidin
qu: Quercetin
RNAi: RNA interference
RSD: Relative standard deviation
S/N: Signal-to-noise ratio
SPA1: Suppressor of phytochrome A-105
ta: Taxifolin
TFA: Trifluoroacetic acid
THF: Tetrahydrofuran
TOF: Time-of-flight
TT: Transparent testa
TTG1: Transparent testa glabra 1
WD40 domain: Consists of WD40 repeats
WD40 repeat: Usually consists of about 40 amino acids often ending with W-D (tryptophan-aspartic acid).
