# Supplementary material for: A fast and simple LC-MS-based characterization of the flavonoid biosynthesis pathway for few seed(ling)s
Source: BMC Plant Biol. 2016 Sep 1;16(1):190. doi: 10.1186/s12870-016-0880-7 (PMC5007998; doi:10.1186/s12870-016-0880-7)
Supplement: Additional file 1: Table S1. — Optimizing for high throughput. List of external standards; and Table S2. Transition parameters. (PDF 381 kb) [file 12870_2016_880_MOESM1_ESM.pdf]

Additional_file_1

Table S1. List of external standards.

External standard	Purchased as	Manufacturer	Part-No	Purity [%]
naringenin	naringenin	SAFC (Sigma-Aldrich)	W530098-SAMPLE	98
taxifolin	taxifolin	Sigma	78666-25MG-F	85
kaempferol	kaempferol	Sigma	K0133-10MG	90
quercetin	quercetin	Sigma	Q4951-10G	98
isorhamnetin	isorhamnetin	Roth	7589.1	99
myricetin	myricetin	Sigma	70050-25MG	96
pelargonidin	pelargonidin chloride	Aldrich	P1659-5MG	100
cyanidin	cyanidin chloride	Sigma	79457-1MG-F	95
delphinidin	delphinidin chloride	Sigma	43725-1MG-F	95
catechin	(+)-catechin hydrate	Sigma	C1251-5G	98
epicatechin	(-)-epicatechin	Sigma	E1753-1G	90
procyanidin B2	procyanidin B2	Fluka (Sigma-Aldrich)	42157-1MG-F	90

Table S2. Transition parameters.

Q1 [Da]	Q2 [Da]	dwell time [ms]	name	declustering potential [V]	entrance potential [V]	collision energy [V]	exit potential [V]
273.1	153	10	naringenin 1	250	10	33	14
273.1	147	10	naringenin 2	250	10	29	14
305.3	153	10	taxifoline 1	96	10	25	14
305.3	259.3	10	taxifoline 2	91	10	17	18
287	153	10	kaempferol 1	120	10	45	12
287	121	10	kaempferol 2	120	10	43	8
303	153	10	quercetin 1	250	10	43	6
303	137	10	Quercetin 2	250	10	43	12
317	153	10	isorhamnetin 1	181	10	45	18
317	229	10	isorhamnetin 2	191	10	43	18
319	153	10	myricetin 1	120	10	45	10
319	217	10	myricetin 2	120	10	47	14
271	121	10	pelargonidin 1	120	10	57	20
271	141	10	pelargonidin 2	120	10	49	10
286.95	136.95	10	cyanidine 1	120	10	45	10
286.95	213	10	cyanidine 2	120	10	45	14
303	229	10	delphinidin 1	120	10	43	14
303	173	10	delphinidin 2	120	10	47	14
291.1	138.9	10	(epi)catechin 1 (used for catechin)	120	10	21	12
291.1	123	10	(epi)catechin 2 (used for epicatechin)	120	10	39	8
579	127.1	10	procyanidin B2 1	191	10	35	14
579	409.1	10	procyanidin B2 2	181	10	27	22
306.1	232.1	10	D3 quercetin 1	111	10	43	12
306.1	154	10	D3 quercetin 2	91	10	45	14
290.1	169.9	10	D3 sakuranetin 1	56	10	33	14
290.1	146.9	10	D3 sakuranetin 2	51	10	29	12

Underlined: quantifier, not underlined: qualifier. For catechin/epicatechin the opposite than the one selected is the qualifier.
