# Supplementary material for: A fast and simple LC-MS-based characterization of the flavonoid biosynthesis pathway for few seed(ling)s
Source: BMC Plant Biol. 2016 Sep 1;16(1):190. doi: 10.1186/s12870-016-0880-7 (PMC5007998; doi:10.1186/s12870-016-0880-7)
Supplement: Additional file 2: Figure S1. — Concept of hydrolysis. Reduced complexity of extracts after hydrolysis; Table S3. Calculated m/z values for detection of glycosylated flavonoids; Table S4. Glycosylated flavonoids in extracted non-hydrolysed samples from A. thaliana seeds; Table S5. Glycosylated flavonoids in extracted non-hydrolysed samples from A. thaliana seedlings; and Table S6. Calculated m/z values for selected substances. (PDF 1704 kb) [file 12870_2016_880_MOESM2_ESM.pdf]

Additional_file_2

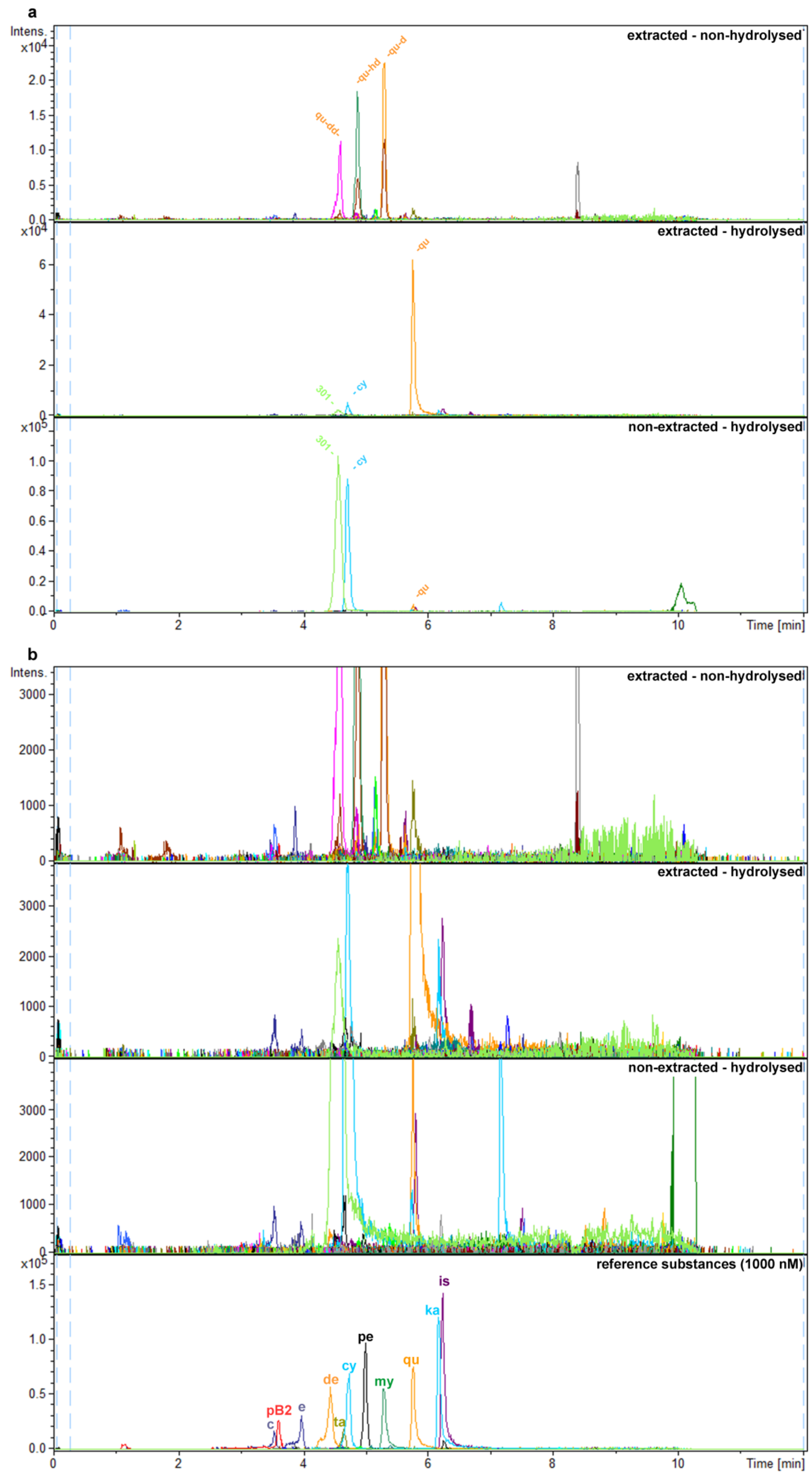

Figure S1: Reduced complexity of extracts after hydrolysis.
a,b Hydrolysis reduces complexity of samples. Exemplary results from LC-ESI-MS-QTOF(AutoMSMS) of an extracted non-hydrolysed sample from ten *A. thaliana* seeds prior to hydrolysis, after hydrolysis and a non-extracted hydrolysed sample. All samples originate from the same ten seeds. Shown are all EICs for all *m/z* values documented in Table S3,S6 covering all glycosylated flavonoids analyzed in Fig. 1c and all substances shown in Fig. 1a. In b a zoom into the lower responses is shown. Note the dominant response of quercetin-based glycosides in extracted non-hydrolysed samples and of quercetin in extracted hydrolysed samples as well as of cyanidin in non-extracted hydrolysed samples. The comparably high green peak in the latter sample corresponds to the m/z value of peonidin which is a methylated derivate of cyanidin. Quercetin-based glycosides were identified as described for Fig. 1c, Table S4,5. Cyanidin and quercetin were identified using the selected external standards.

ta: taxifolin, ka: kaempferol, qu: quercetin, is: isorhamnetin, my: myricetin, pe: pelargonidin, cy: cyanidin, de: delphinidin, c: catechin, e: epicatechin, pB: procyanidinB2.

Table S3. Calculated *m/z* values for detection of glycosylated flavonoids.

core component: sugar(s) added:	naringenin	taxifolin	kaempferol	quercetin	isorhamnetin	myricetin	pelargonidin	cyanidin	delphinidin	catechin/epicatechin	procyanidin
p	405.7997	437.1079	419.0973	435.0922	449.1079	451.0871	403.1024	419.0973	435.0922	423.1286	711.1920
d	419.8153	451.1235	433.1129	449.1078	463.1235	465.1027	417.1180	433.1129	449.1078	437.1442	725.2076
h	435.8102	467.1184	449.1078	465.1027	479.1184	481.0976	433.1129	449.1078	465.1027	453.1391	741.2025
pp	537.8420	569.1502	551.1396	567.1345	581.1502	583.1294	535.1447	551.1396	567.1345	555.1709	843.2343
dp	551.8576	583.1658	565.1552	581.1501	595.1658	597.1450	549.1603	565.1552	581.1501	569.1865	857.2499
dd	565.8732	597.1814	579.1708	595.1657	609.1814	611.1606	563.1759	579.1708	595.1657	583.2021	871.2655
hp	567.8525	599.1607	581.1501	597.1450	611.1607	613.1399	565.1552	581.1501	597.1450	585.1814	873.2448
hd	581.8681	613.1763	595.1657	611.1606	625.1763	627.1555	579.1708	595.1657	611.1606	599.1970	887.2604
hh	597.8630	629.1712	611.1606	627.1555	641.1712	643.1504	595.1657	611.1606	627.1555	615.1919	903.2553
ppp	669.8843	701.1925	683.1819	699.1768	713.1925	715.1717	667.1870	683.1819	699.1768	687.2132	975.2766
dpp	683.8999	715.2081	697.1975	713.1924	727.2081	729.1873	681.2026	697.1975	713.1924	701.2288	989.2922
ddp	697.9155	729.2237	711.2131	727.2080	741.2237	743.2029	695.2182	711.2131	727.2080	715.2444	1003.3078
hpp	699.8948	731.2030	713.1924	729.1873	743.2030	745.1822	697.1975	713.1924	729.1873	717.2237	1005.2871
ddd	711.9311	743.2393	725.2287	741.2236	755.2393	757.2185	709.2338	725.2287	741.2236	729.2600	1017.3234
hdp	713.9104	745.2186	727.2080	743.2029	757.2186	759.1978	711.2131	727.2080	743.2029	731.2393	1019.3027
hdd	727.9260	759.2342	741.2236	757.2185	771.2342	773.2134	725.2287	741.2236	757.2185	745.2549	1033.3183
hhp	729.9053	761.2135	743.2029	759.1978	773.2135	775.1927	727.2080	743.2029	759.1978	747.2342	1035.2976
hhd	743.9209	775.2291	757.2185	773.2134	787.2291	789.2083	741.2236	757.2185	773.2134	761.2498	1049.3132
hhh	759.9158	791.2240	773.2134	789.2083	803.2240	805.2032	757.2185	773.2134	789.2083	777.2447	1065.3081
pppp	801.9266	833.2348	815.2242	831.2191	845.2348	847.2140	799.2293	815.2242	831.2191	819.2555	1107.3189
dppp	815.9422	847.2504	829.2398	845.2347	859.2504	861.2296	813.2449	829.2398	845.2347	833.2711	1121.3345
ddpp	829.9578	861.2660	843.2554	859.2503	873.2660	875.2452	827.2605	843.2554	859.2503	847.2867	1135.3501
hppp	831.9371	863.2453	845.2347	861.2296	875.2453	877.2245	829.2398	845.2347	861.2296	849.2660	1137.3294
dddp	843.9734	875.2816	857.2710	873.2659	887.2816	889.2608	841.2761	857.2710	873.2659	861.3023	1149.3657
hdpp	845.9527	877.2609	859.2503	875.2452	889.2609	891.2401	843.2554	859.2503	875.2452	863.2816	1151.3450
dddd	857.9890	889.2972	871.2866	887.2815	901.2972	903.2764	855.2917	871.2866	887.2815	875.3179	1163.3813
hddp	859.9683	891.2765	873.2659	889.2608	903.2765	905.2557	857.2710	873.2659	889.2608	877.2972	1165.3606
hhpp	861.9476	893.2558	875.2452	891.2401	905.2558	907.2350	859.2503	875.2452	891.2401	879.2765	1167.3399
hddd	873.9839	905.2921	887.2815	903.2764	917.2921	919.2713	871.2866	887.2815	903.2764	891.3128	1179.3762
hhdp	875.9632	907.2714	889.2608	905.2557	919.2714	921.2506	873.2659	889.2608	905.2557	893.2921	1181.3555
hhdd	889.9788	921.2870	903.2764	919.2713	933.2870	935.2662	887.2815	903.2764	919.2713	907.3077	1195.3711
hhhp	891.9581	923.2663	905.2557	921.2506	935.2663	937.2455	889.2608	905.2557	921.2506	909.2870	1197.3504
hhhd	905.9737	937.2819	919.2713	935.2662	949.2819	951.2611	903.2764	919.2713	935.2662	923.3026	1211.3660
hhhh	921.9686	953.2768	935.2662	951.2611	965.2768	967.2560	919.2713	935.2662	951.2611	939.2975	1227.3609
**m/z for aglycon**	273.7574	305.0656	287.0550	303.0499	317.0656	319.0448	271.0601	287.0550	303.0499	291.0863	579.14968

m/z values [M+H]+ calculated for the set of selected substances glycosylated with different combinations of pentoses, deoxyhexoses and hexoses. p=pentose, d=deoxyhexose, h= hexose

Table S4. Glycosylated flavonoids in extracted non-hydrolysed samples from *A. thaliana* seeds

	AutoMSMS		ISCID		Predicted/identified glycoside - level of prediction/identification -						Possible larger glycoside - level of prediction/identification -				
***Rt* [min]**	**ESI-MS [*m/z*] max**	**Fragment [*m/z*]**	**Fragment [*m/z*]**	**Aglycon [*m/z*]**	**Aglycon**	**Sugar**	**Max *m/z***	**Highest accuracy [*m/z*]**	**(Predicted) aglycon**	**Sugar modifi-cation**	**Max *m/z* (lower accuracy)**	**(Predict-ted) aglycon**	**Sugar modifi-cation**	**Max *m/z***	**Overlapping EIC peaks (same *m/z*)**
3.4	453.1391	453.1391		291.0879	predicted	calculated	co-o	453.1391	(c/e)	h					**+**
		291.0879	291.0879												
3.63	599.197	599.197		291.0879	predicted	calculated	co-o	599.197	(c/e)	hd					**+**
		453.1391													
		437.1442	437.1442												
		291.0879	291.0879												
4.17	757.2185	757.2185		303.0499	predicted	calculated	co-o, sigma <50	757.2185	(qu/de)	hdd					**+**
		611.1606	611.1606												
		449.1078	449.1078												
			303.0499												
4.19	611.1606	611.1606	611.1606	303.0499	predicted	calculated	co-o	611.1606	(qu/de)	hd					**+**
			465.1025												
			449.1078												
			303.0499												
4.67	773.2134	773.2134		303.0499	predicted	calculated	sfs	611.1606	(qu/de)	hd	773.2134	(qu/de)	hhd	co-o	+
		611.1606	611.1606												
		449.1022	449.1022												
		303.0499	303.0499												
4.71	611.1606	611.1606 (54.4 eV: 303.0502 (100))	611.1606 (54.4 eV: 303.0501 (100))	303.0499	identified	MSMS	MSMS	611.1606	qu	hd					**+**
			465.1025												
		449.1078	449.1022 (48.1 eV: 303.0499 (100))												
		303.0499	303.0499 (42.6 eV 303.0497 (75), 229.0497												

Table S4. Glycosylated flavonoids in extracted non-hydrolysed samples from *A. thaliana* seeds

			(100),<br>201.0552<br>(50),<br>153.0193<br>(75))												
4.99	625.1763	625.1763	625.1763	317.0656	predicted	calculated	co-o, sigma<br><50	625.1763	(is)	hd					-
		463.1235	463.1235												
		317.0656	317.0656												
4.99	433.1126	433.1126	433.1126	287.0550	predicted	calculated	co-o	433.1126	(ka/cy)	d					-
			287.0550												
5.00	741.2236	741.2236	741.2236	303.0499	identified	MSMS	MSMS	595. 1657	qu	dd	741.2236	qu	ddd	co-o	-
		595.1657<br>(53.8 eV:<br>303.0501<br>(100))	595.1657<br>(53.8 eV:<br>303.0499<br>(100))												
		449.1022	449.1022<br>(48.1 eV:<br>303.0499<br>(100))												
		303.0499	303.0499<br>(42.6 eV:<br>303.0512<br>(83), 229.05<br>(100),<br>201.0551<br>(45),<br>153.0191<br>(74))												
5.27	579.1711	579.1711	579.1711	287.0550	predicted	calculated	sfs	579.1711	(ka/cy)	dd					-
		433.1126	433.1126												
		287.0550	287.0550												
5.31	609.1814	609.1814	609.1814	317.0656	predicted	calculated	co-o, sigma<br><10	609.1814	(is)	dd					-
		463.1235	463.1235												
		317.0656	317.0656												
5.42	595.1657	595.1657		303.0499	identified	calculated	MSMS	449.1022	qu	d	595. 1657	qu	dd	co-o,<br>sigma<br><50	-
		449.1022	449.1022												

Table S4. Glycosylated flavonoids in extracted non-hydrolysed samples from *A. thaliana* seeds

		(48.1 eV: 303.0502 (100))									
		303.0499	303.0499 (42.6 eV: 303.0512 (78), 229.05 (100), 201.0551 (69), 153.0191 (36))								
5.76	433.1126	433.1126		287.0550	predicted	calculated	co-o	433.1126	(ka/cy)	d	-
		287.0550	287.0550								
5.77	463.1235	463.1235	463.1235	317.0656	predicted	calculated	co-o, sigma <10	463.1235	(is)	d	-
		317.0656	317.0656								

5 seeds were analysed. *m/z* values: [M+H]+. Fragments were detected by analysing the given calculated EICs. For further fragmentations, the detected exact masses and collision energy are given in brackets. Brackets behind subfragments indicate percentages of fragment intensities. Aglycons were either predicted by the detected m/z or identified by MSMS. Predicted aglycons are written in brackets. These could as well be other substances of the same m/z. Measurements were done with LC-ESI-MS-QTOF(AutoMSMS) or LC-ESI-MS-QTOF(pseudoMS^3) (here labelled with ISCID) method.

Underlined: the fragment with the strongest response in the AutoMSMS experiment; **identified:** either MSMS or smart formula highest accuracy; **predicted:** all other mentioned methods.

Attached sugars were defined as follows. **calculated:** co-occurring masses at the same retention time differ by the size of a neutral loss (hexose or deoxy-hexose), **MSMS:** an MSMS has been recorded and a neutral loss of the size of a hexose or deoxy-hexose was observed.

The maximal given m/z was detected as follows. **co-o:** co-occurrence: peaks from calculated EICs of the listed size co-occur at the same retention time and peak shapes are similar; **sfe:** In addition, for the fragment of maximal size, smartformula function of the Compass DataAnalysis software gives an error of less than 1ppm; **sigma:** smart formula function of Compass DataAnalysis software gives additionally a sigma value as indicated; **MSMS:** an MSMS of the fragment of maximal mass was recorded and releases a mass corresponding to that of the aglycon.

ISCID: in source collision-induced dissociation, h: hexose, d: deoxy-hexose ta: taxifolin, cy: cyanidin, ka: kaempferol, qu: quercetin, is: isorhamnetin, e/c: epicatechin or catechin, de: delphinidin.

Table S5. Glycosylated flavonoids in soluble, non-hydrolysed samples from *A. thaliana* seedlings

	AutoMSMS		ISCID		Predicted/identified glycoside - level of prediction/identification -						Possible larger glycoside - level of prediction/identification -				
Rt [min]	ESI-MS [*m/z*] max	Fragment [*m/z*]	Fragment [*m/z*]	Aglycon [*m/z*]	Aglycon	Sugar	Max *m/z*	Highest accuracy [*m/z*]	(Predicted) aglycon	Sugar modifi-cation	Max *m/z* (lower accuracy)	(Predict-ted) aglycon	Sugar modifi-cation	Max *m/z*	Overlapping EIC peaks (same *m/z*)
3.55	467.1184	467.1184		305.0656	predicted	calculated	co-o	467.1184	(ta)	h	-				-
		305.0656	305.0656												
3.64	599.197	599.197		291.0863	predicted	calculated	co-o	599.197	(c/e)	hd					-
		453.1391													
		437.1442	437.1442												
		291.0863	291.0863												
3.93	627.1556	627.1556	627.1556	303.0499	predicted	calculated	sfe	627.1556	(qu/de)	hh	-				-
			465.1027												
			303.0499												
3.98	757.2185	757.2185	757.2185	303.0499	predicted	calculated	co-o	757.2185	(qu/de)	hdd	-				+
		611.1606	611.1606												
		595.1657													
		449.1078	449.1078												
			303.0499												
4.14	741.2236	741.2236	741.2236	287.055	predicted	MSMS	sfe	741.2236	(ka/cy)	hdd					+
		595.1657	595.1657 (53.81 eV: 287.0549(100))												
		433.1129	433.1129 (47.49 eV: 287.0545												

Table S5. Glycosylated flavonoids in soluble, non-hydrolysed samples from *A. thaliana* seedlings

			(100))												
		287.055	287.0550												
4.18	757.2185	757.2185	757.2185	303.0499	predicted	calculated	sfe	757.2185	(qu/de)	hdd	-				+
			611.1606												
			465.1025												
		449.1079	449.1078												
		303.0499	303.0499												
4.35	887.2815	887.2815.	887.2815	287.055	identified	MSMS	MSMS	741.2236	ka	hdd	887.2815	ka	hddd	sfe	-
		741.2236 (59.65 eV: 287.0546 (100))	741.2236												
		595.1657 (53.81 eV: 287.0546 (100))	595.1657 (53.81 eV: 287.0546 (100))												
		579.1708	579.1708												
			449.1078												
		433.1126	433.1126 (47.5eV: 287.0548(100))												
		287.055	287.0550 (42.01 eV: 287.0538 (100), 213.0545 (40), 153.0187												

Table S5. Glycosylated flavonoids in soluble, non-hydrolysed samples from *A. thaliana* seedlings

			(44))												
4.49	595.1688	595.1688	595.1688	287.055	predicted	MSMS	sfe	595.1688	(ka/cy)	hd					+
		449.1022	449.1022 (48.09 eV: 287.0547(100), 303.0499(27))												
			287.0576												
4.57	611.1606	611.1606 (54.45 eV: 303.0504 (100))	611.1606	303.0499	identified	MSMS	MSMS	611.1606	qu	hd	-				+
		449.1022	449.1022 (48.09 eV: 303.0498(100))												
			303.0499 (42.61: 303.0505 (79), 229.049 (100), 153.0178 (37))												
4.71	611.1606	757.2185	611.1606 (54.45 eV: 303.0501 (100))	303.0499	identified	MSMS	MSMS	611.1606	qu	hd	757.2185	qu	hdd	co-o	+
		611.1606 (54.45 eV: 303.0501 (100))	449.1022 (48.1 eV: 303.0499 (100))												

Table S5. Glycosylated flavonoids in soluble, non-hydrolysed samples from *A. thaliana* seedlings

		449.1023	303.0499 (42.62: 303.0516 (76), 229.0501 (100), 153.0185 (55))												
		303.0499													
4.97	595.1655	595.1655 (53.82 eV: 287.0550 (100))	595.1655 (53.82 eV: 287.0549 (100))	287.055	identified	MSMS	MSMS	595.1655	ka	hd					-
		433.1128	433.1128 (47.5 eV: 287.0548 (100))												
		287.055	287.0552 (42.02 eV: 287.0552 (100), 213.0551 (41), 153.0188 (46))												
4.99	625.1763	625.1763	625.1763	317.0656	predicted	calculated	sfs	625.1763	(is)	hd					-
		463.1235	479.1184												
			463.1235												
			317.0656												
5.01	449.1022	741.2236		303.0499	predicted	MSMS		449.1022	(qu/de)	d	741.2236	(qu/de)	ddd	sfe	+
		449.1022	449.1022 (48.1 eV: 303.0499												

Table S5. Glycosylated flavonoids in soluble, non-hydrolysed samples from *A. thaliana* seedlings

			(100))												
		303.0499	303.0499												
5.11	611.1606	611.1606	611.1606	303.0499	predicted	calculated	co-o	611.1606	(qu/de)	hd	-				+
		465.1025	465.1025												
			449.1022												
		303.0499	303.0499												
5.17	465.1025	465.1025	465.1025	303.0499	predicted	calculated	co-o	465.1025	(qu/de)	h	-				+
		303.0499	303.0499												
5.28	579.1711	725.2287	725.2287	287.055	identified	MSMS	MSMS	579.1711	ka	dd	725.2287	ka	ddd	sfe	-
		579.1711 (53.18 eV: 287.055 (100))	579.1711 (53.17 eV: 287.0554 (100))												
		433.1129 (47.49 eV: 287.0553 (100))	433.1129 (47.5eV: 287.0548 (100))												
		287.0554	287.0554 (42.02 eV: 287.0554 (100), 213.0549 (30), 153.0188 (41))												
5.31	609.1814	609.1814	609.1814	317.0656	predicted	calculated	co-o	609.1814	(is)	dd					-
		463.1235	463.1235												
			317.0656												

Table S5. Glycosylated flavonoids in soluble, non-hydrolysed samples from *A. thaliana* seedlings

5.46	449.1088	449.1088	449.1088	287.055	identified	calculated	co-o	449.1088	cy	h					-
		287.055	287.055 (42.01 eV: 287.0557 (100), 213.0543 (26), 153.0188 (0))												
5.47	625.1763	625.1763	625.1763	317.0656	predicted	calculated	co-o	479.1184	(is)	h	625.1763	(is)	hd	co-o	-
		479.1184	317.0656												
		317.0656													
5.76	579.1708	579.1711		287.055	predicted	calculated	co-o	433.1129	(ka/cy)	d	579.1708	(ka/cy)	dd	sfe	-
		433.1129	433.1129												
		287.0550	287.055												
6.11	433.1129	433.1129	287.055	287.055	predicted	calculated	co-o, sigma <40	433.1129	(ka/cy)	d					-
		287.055													
6.16	463.1235	463.1235	317.066	317.0656	predicted	calculated	co-o	463.1235	(is)	d					-
3.55	467.1184	467.1184		305.0656	predicted	calculated	co-o	467.1184	(ta)	h	-				-
		305.0656	305.0656												
3.64	599.197	599.197		291.0863	predicted	calculated	co-o	599.197	(c/e)	hd					-
		453.1391													
		437.1442	437.1442												
		291.0863	291.0863												

Table S5. Glycosylated flavonoids in soluble, non-hydrolysed samples from *A. thaliana* seedlings

3.93	627.1556	627.1556	627.1556	303.0499	predicted	calculated	sfe	627.1556	(qu/de)	hh	-	-
			465.1027									
			303.0499									
3.98	757.2185	757.2185	757.2185	303.0499	predicted	calculated	co-o	757.2185	(qu/de)	hdd	-	+
		611.1606	611.1606									
		595.1657										
		449.1078	449.1078									
			303.0499									

10 and 20 seedlings were analysed. *m/z* values: [M+H]+. Fragments were detected by analysing the given calculated EICs. For further fragmentations, the detected exact masses and collision energy are given in brackets. Brackets behind subfragments indicate percentages of fragment intensities. Aglycons were either predicted by the detected m/z or identified by MSMS. Predicted aglycons are written in brackets. These could as well be other substances of the same m/z. Measurements were done with LC-ESI-MS-QTOF(AutoMSMS) or LC-ESI-MS-QTOF(pseudoMS^3) (here labelled with ISCID) method.

Underlined: the fragment with the strongest response in the AutoMSMS experiment; **identified:** either MSMS or smart formula highest accuracy; **predicted:** all other mentioned methods.

Attached sugars were defined as follows. **calculated:** co-occurring masses at the same retention time differ by the size of a neutral loss (hexose or deoxy-hexose), **MSMS:** an MSMS has been recorded and a neutral loss of the size of a hexose or deoxy-hexose was observed.

The maximal given m/z was detected as follows. **co-o:** co-occurrence: peaks from calculated EICs of the listed size co-occur at the same retention time and peak shapes are similar; **sfe:** In addition, for the fragment of maximal size, smartformula function of the Compass DataAnalysis software gives an error of less than 1ppm; **sigma:** smart formula function of Compass DataAnalysis software gives additionally a sigma value as indicated; **MSMS:** an MSMS of the fragment of maximal mass was recorded and releases a mass corresponding to that of the aglycon.

ISCID: in source collision-induced dissociation, h: hexose, d: deoxy-hexose ta: taxifolin, cy: cyanidin, ka: kaempferol, qu: quercetin, is: isorhamnetin, e/c: epicatechin or catechin, de: delphinidin.

Table S6. Calculated *m/z* values for selected substances.

m/z $[M+H]^+$	name of substance	comment
271.0601	pelargonidin	external standard
273.7574	naringenin	external standard
287.0550	kaempferol	external standard
287.0550	cyanidin	external standard
291.0863	catechin/epicatechin	external standard
303.0499	quercetin	external standard
303.0499	delphinidin	external standard
305.0656	taxifolin	external standard
317.0656	isorhamnetin	external standard
319.0448	myricetin	external standard
579.1497	procyanidin (B1. B2)	external standard
867.2131	procyanidin trimer (C1. C2 and others)	proanthocyanidin
547.1599	propelargonidin dimer	proanthocyanidin
819.2283	propelargonidin trimer	proanthocyanidin
611.1395	prodelphinidin dimer	proanthocyanidin
915.1978	prodelphinidin trimer	proanthocyanidin
271.0601	apigenin	other (from pathway. see Figure 1)
273.7574	chalcone (tetrahydroxy-chalcone)	other (from pathway. see Figure 1)
275.0914	afzelechin	other (from pathway. see Figure 1)
275.0914	epiafzelechin	other (from pathway. see Figure 1)
287.0550	luteolin	other (from pathway. see Figure 1)
289.0707	dihydrokaempferol	other (from pathway. see Figure 1)
289.0707	eriodictyol	other (from pathway. see Figure 1)
291.0863	leucopelargonidin	other (from pathway. see Figure 1)
301.0707	peonidin	other (from pathway. see Figure 1)
305.0656	pentahydroxyflavanone (dihydrotricetin)	other (from pathway. see Figure 1)
307.0812	leucocyanidin	other (from pathway. see Figure 1)
307.0812	epigallocatechin	other (from pathway. see Figure 1)
307.0812	gallocatechin	other (from pathway. see Figure 1)
317.0656	petunidin	other (from pathway. see Figure 1)
321.0605	dihydromyricetin	other (from pathway. see Figure 1)
323.0761	leucodelphinidin	other (from pathway. see Figure 1)
331.0811	malvidin	other (from pathway. see Figure 1)
290.1102	D3-sakuranetin	internal standard
306.0664	D3-quercetin	internal standard

For all used m/z ($[M+H]^+$) for glycosylated core substances, see Table S3.
m/z values according to METLIN (Scripps Center for Metabolomics and Mass Spectrometry, https://metlin.scripps.edu/metabo_advanced.php) or calculated using MolE - Molecular Mass Calculator v2.02 (http://mods.rna.albany.edu/masspec/MolE) by Jef Rozenski (1999).
