# Supplementary material for: A fast and simple LC-MS-based characterization of the flavonoid biosynthesis pathway for few seed(ling)s
Source: BMC Plant Biol. 2016 Sep 1;16(1):190. doi: 10.1186/s12870-016-0880-7 (PMC5007998; doi:10.1186/s12870-016-0880-7)
Supplement: Additional file 3: Figure S2. — External and Internal standards. EICs of external standards; Figure S3. Linearity and EICs of deuterated, internal standards; Table S7. Stability of standards over time and in dependence of acid, Table S8. Precision and linearity of the selected external standards; and Methods S1. Internal standard synthesis. (PDF 1682 kb) [file 12870_2016_880_MOESM3_ESM.pdf]

Additional_file_3

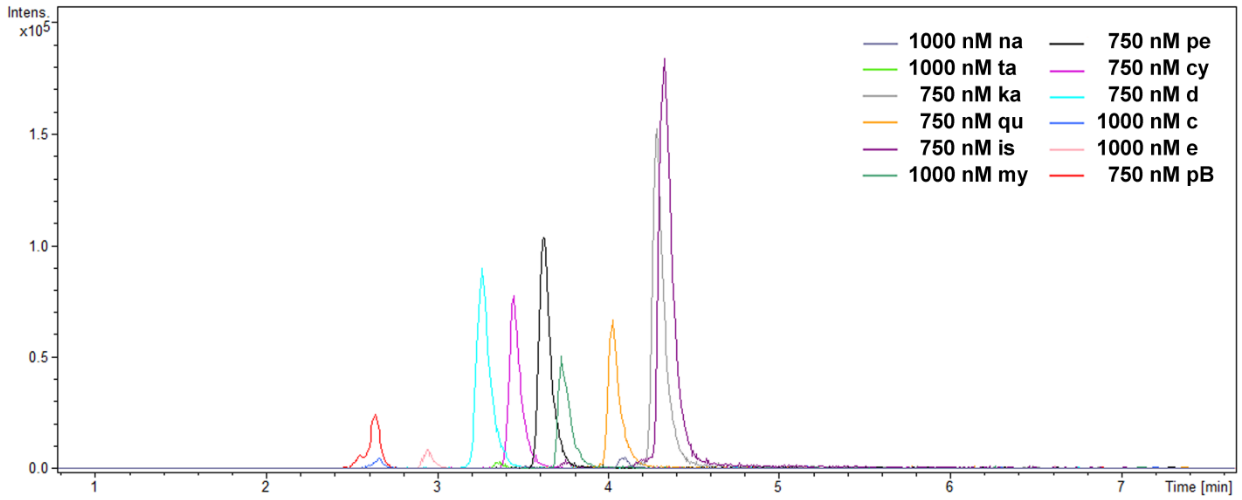

Figure S2: EICs of external standards.
Merged EICs of the 12 selected external standards from LC-ESI-MS-QTOF(pseudoMS³) analysis. Each standard was analyzed as a separate sample. Note that different concentrations were chosen for presentation due to the strong differing responses of these substances. MRMs for the LC-ESI-MS-QTRAP(MRM) setup are documented in Table S2. Examples of catechin and epicatechin separation can be found in Fig. 3.

ta: taxifolin, ka: kaempferol, qu: quercetin, is: isorhamnetin, my: myricetin, pe: pelargonidin, cy: cyanidin, de: delphinidin, c: catechin, e: epicatechin, pB: procyanidinB2.

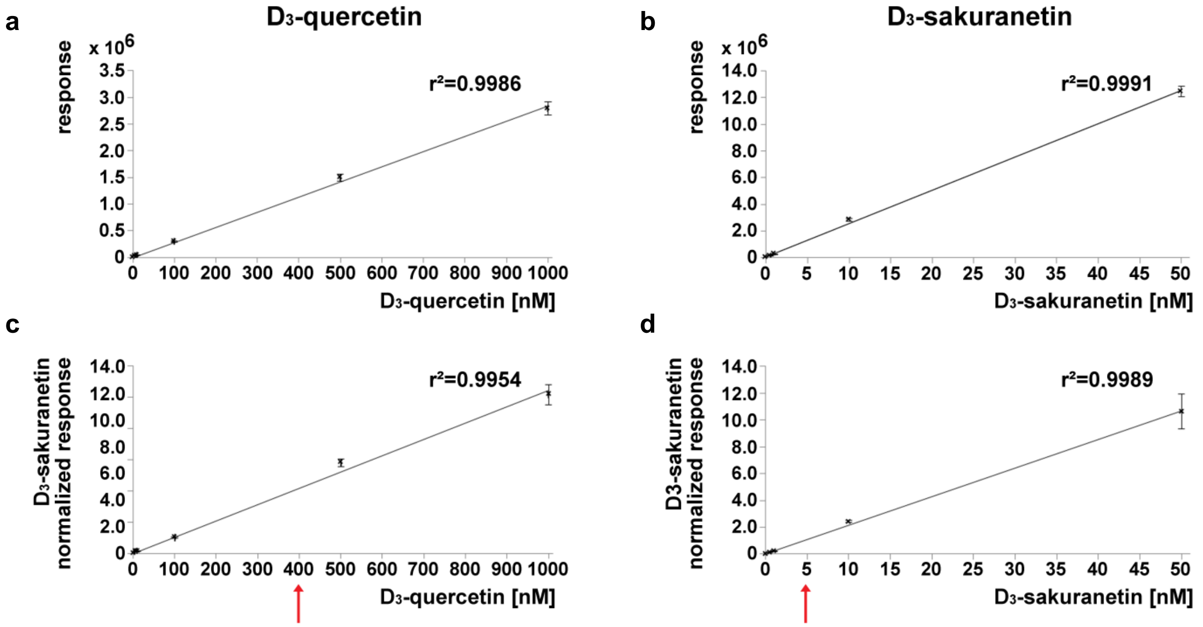

e

D3-quercetin [nM]	RSD (A) [%]	RSD (C) [%]
5	3.20	5.89
10	15.66	15.94
100	3.57	5.54
500	4.37	4.27
1000	4.35	6.29

5 nM D3-sakuranetin (all samples)
RSD [%] response
6.42

f

D3-sakuranetin [nM]	RSD (B) [%]	RSD (D) [%]
0.5	3.13	10.08
1.0	5.47	10.30
10.0	3.36	3.66
50.0	3.04	12.12

5 nM D3-quercetin (all samples)
RSD [%] response
10.59

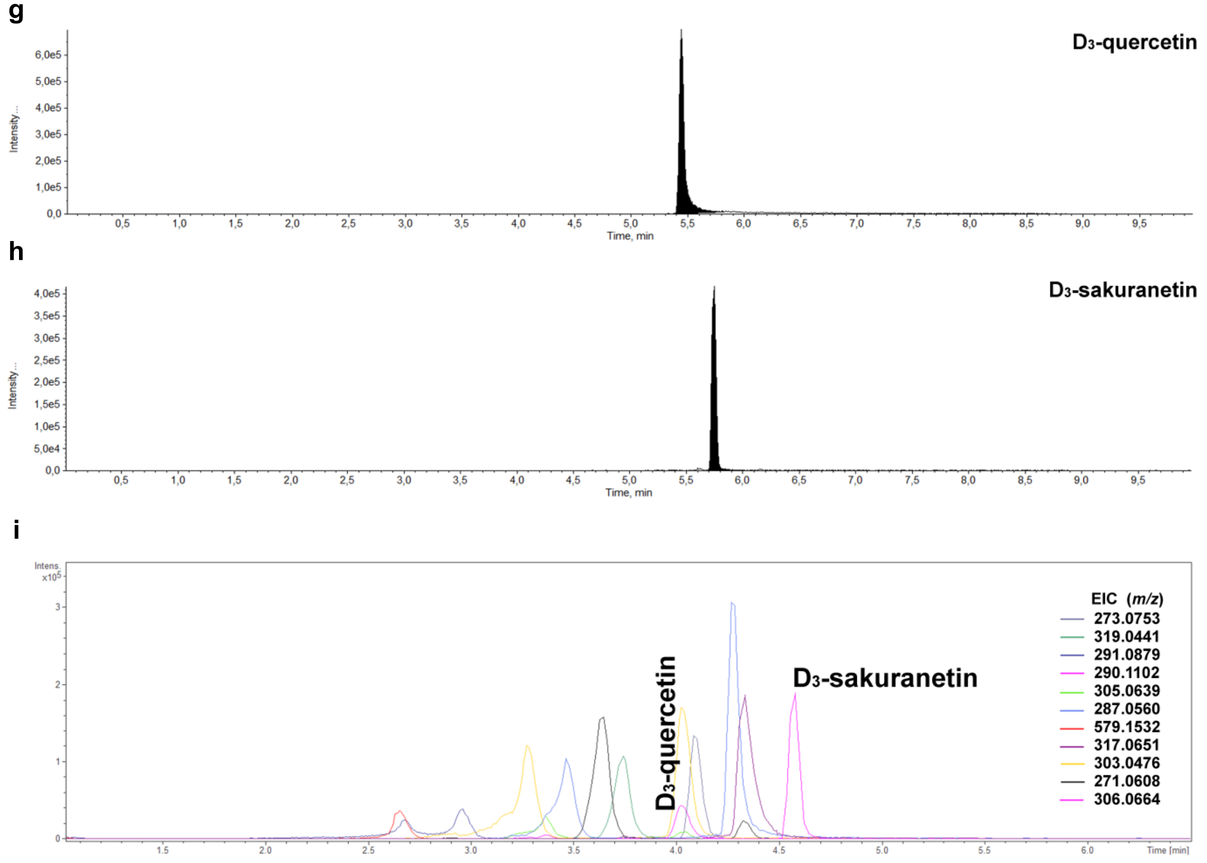

Figure S3: Linearity and EICs of deuterated, internal standards. Linearity of both internal standards (n=3) using MRMs with the LC-ESI-MS-QTRAP(MRM) setup with r^2-values above 0.99 for both internal standards when analyzing the responses (**a,b**) or normalized responses (normalization with the respective other internal standard) (**c,d**). The analyzed concentration in triplicates can be seen in **e** and **f**, respectively as well as the concentration of the normalizing internal standard through all samples as used in this experiment. A red arrow indicates these concentrations which are in the linear range. Close to these are the calculated concentration within the seed and seedling samples analyzed in this study (e,f) Precision at the different concentrations and over all samples for the respective other internal standards used for normalization. Selected MRMs for D_3-quercetin (**g**) and D_3-sakuranetin (**h**) recorded with the LC-ESI-MS-QTRAP(MRM) setup. (**i**) Merged EICs of all external and internal standards with the LC-ESI-MS-QTOF(pseudoMS^3) setup. The two internal standards are marked in magenta.

Table S7. Stability of standards over time and in dependence of acid.

substance	response relative to initial response after 24h at 5°C on sample taker								Welch-test - vs. + FA *P*-value
	- FA				+ FA				
	Mean [%]	STDEV	RSD[%]	n	Mean [%]	STDEV	RSD[%]	n	
**D3-quercetin**	92	15	16	36*	76	23	30	36*	0.0524
**D3-sakuranetin**	95	15	16	36*	100	23	23	36*	0.5155
**naringenin**	133	1	1	3	124	13	11	3	0.3856
**taxifolin**	99	5	5	3	103	4	3	3	0.2792
**kaempferol**	99	2	2	3	98	6	6	3	0.7043
**quercetin**	98	2	2	3	69	5	7	3	0.0034
**isorhamnetin**	100	3	3	3	101	5	5	3	0.8529
**myricetin**	79**	2	3	3	37**	2	6	3	0.00002
**pelargonidin**	20	1	4	3	32	2	7	3	0.0051
**cyanidin**	1	0	7	3	22	1	5	3	0.0008
**delphinidin**	15	1	4	3	22	2	9	3	0.0160
**catechin**	105	3	3	3	103	3	3	3	0.4737
**epicatechin**	128	5	4	3	124	1	1	3	0.3519
**procyanidin B2**	96	7	7	3	121	17	14	3	0.1151

Note, the same sample was measured at 0h and 24h and left on the sample taker of the LC-ESI-MS-QTRAP(MRM) setup. Means are the means of the ratios of D3-sakuranetin-normalized responses of three samples at 24h to 0h. For internal standards the responses without normalization were used. FA: formic acid, N: number of replicates, * 12x3 replicates (internal standard was added to all external standard samples), ** note for myricetin at 0h only one sample was saved due to a technical error and used as a reference for all three samples at 24h. The decrease of myricetin in presence of FA was regularly seen in quality controls over time.

Table S8. Precision and linearity of the selected external standards.

	D3-sakuranetin-normalized responses (n=10)																		
	precision																		linearity
	1 nM			10 nM			50 nM			100 nM			500 nM			1000 nM			1-1000 nM
**external standard**	**mean**	**STDEV**	**RSD [%]**	**mean**	**STDEV**	**RSD [%]**	**mean**	**STDEV**	**RSD [%]**	**mean**	**STDEV**	**RSD [%]**	**mean**	**STDEV**	**RSD [%]**	**mean**	**STDEV**	**RSD [%]**	**r^2**
**naringenin**	2.40E-02	4.46E-03	18.56	2.92E-01	4.55E-02	15.54	1.58E+00	3.19E-01	20.13	2.94E+00	3.10E-01	10.55	1.33E+01	8.55E-01	6.43	2.25E+01	3.02E+00	13.46	0.9925
**taxifolin**	8.12E-03	1.04E-03	12.83	5.08E-02	4.53E-03	8.91	2.95E-01	4.31E-02	14.59	5.84E-01	6.45E-02	11.05	2.90E+00	3.10E-01	10.68	5.73E+00	9.35E-01	16.32	1.0000
**kaempferol**	2.56E-02	2.64E-03	10.32	2.42E-01	2.28E-02	9.41	1.27E+00	1.74E-01	13.74	2.59E+00	2.74E-01	10.59	1.36E+01	1.00E+00	7.39	2.40E+01	3.31E+00	13.84	0.9960
**quercetin**	5.27E-03	1.52E-03	28.79	5.25E-02	1.28E-02	24.43	3.09E-01	7.40E-02	23.98	6.19E-01	1.11E-01	17.98	3.39E+00	2.39E-01	7.04	7.02E+00	8.56E-01	12.19	0.9997
**isorhamnetin**	1.46E-02	2.44E-03	16.65	1.96E-01	2.08E-02	10.59	9.71E-01	1.27E-01	13.13	2.03E+00	1.58E-01	7.77	1.09E+01	6.44E-01	5.90	2.02E+01	2.18E+00	10.83	0.9984
**myricetin**	0	0	0	1.32E-02	2.31E-03	17.42	1.30E-01	2.02E-02	15.52	3.96E-01	4.63E-02	11.69	4.74E+00	4.84E-01	10.21	1.27E+01	1.93E+00	15.20	0.9858
**pelargonidin**	7.63E-03	8.77E-04	11.49	1.84E-01	3.38E-02	18.37	9.17E-01	1.73E-01	18.85	1.89E+00	2.67E-01	14.14	7.40E+00	9.49E-01	12.82	1.25E+01	2.03E+00	16.18	0.9920
**cyanidin**	7.04E-03	8.64E-04	12.27	1.04E-01	1.92E-02	18.55	6.12E-01	1.23E-01	20.09	1.13E+00	2.18E-01	19.31	5.87E+00	6.74E-01	11.47	1.07E+01	1.54E+00	14.30	0.9980
**delphinidin**	6.58E-03	1.84E-03	27.97	7.75E-02	9.77E-03	12.60	4.76E-01	1.07E-01	22.52	1.14E+00	1.64E-01	14.41	6.72E+00	1.05E+00	15.59	1.44E+01	1.63E+00	11.34	0.9990
**catechin**	8.67E-03	2.15E-03	24.75	1.70E-01	4.58E-02	27.00	1.10E+00	2.27E-01	20.65	2.19E+00	2.60E-01	11.89	1.03E+01	9.28E-01	9.01	1.96E+01	2.48E+00	12.65	0.9993
**epicatechin**	1.01E-02	2.33E-03	23.14	1.17E-01	1.86E-02	15.84	6.97E-01	1.04E-01	14.93	1.44E+00	2.08E-01	14.51	6.75E+00	4.91E-01	7.28	1.27E+01	1.55E+00	12.15	0.9991
**procyanidin B2**	5.63E-02	7.41E-03	13.17	1.90E-01	2.13E-02	11.21	1.13E+00	1.81E-01	16.01	2.28E+00	2.50E-01	10.94	1.08E+01	7.30E-01	6.79	2.00E+01	2.91E+00	14.55	0.9987

For precision, each value is derived from ten replicates of a mix of all external standards at the indicated concentrations and 5 nM D3-sakuranetin in MeOH+1% FA. A stock was diluted which comprised the external standards at a concentration of 5µM each. For each substance the selected MRM was used (See Table S2). Linearity is based on the precision experiment. Minimal and maximal RSD and r^2 values are underlined. For this, the not detected myricetin at 1nM was excluded.

Methods S1

Synthesis of internal standards - general experimental

Unless otherwise stated all ^{1}H and ^{13}C NMR spectra were recorded at room temperature in $CDCl_3$ on *Bruker* instruments (Avance DPX 300, Avance II 300, Avance DRX 500 or Avance II 600, Bruker Daltonic, www.bruker.com). Chemical shifts (δ) are reported in parts per million (ppm) from tetramethylsilane using the residual solvent resonance ($CDCl_3$: 7.26 ppm for ^{1}H NMR, 77.0 ppm for ^{13}C NMR) or TMS as the internal standard. Multiplicities are abbreviated as follows: s = singlet, d = doublet, t = triplet, q = quartet, m = multiplet, br = broad). IR spectra were recorded on a *Perkin-Elmer* Paragon 1000 FT-IR spectrometer (http://www.perkinelmer.com) in the ATR mode at room temperature. Relative intensities of the signals are given as very strong (ss), strong (s), medium (m), weak (w) and broad (br). Mass spectra were recorded at the Analytical Service Unit, University of Cologne on *Finnigan* instruments (MAT Inocs 50 galaxy system (for EI LR-MS) and a MAT 900 (for HR-MS), http://www.thermoscientific.com). ESI-MS experiments were performed on a modified *Thermo Fisher LTQ Orbitrap XL* (http://www.thermoscientific.com) instrument. The spray voltage (3.4 kV), capillary voltage (3.0 V), tube lens voltage (3.0 V) and capillary temperature of 275 °C were applied as ESI conditions. To generate a stable electrospray sheath gas and sweep gas were used (Nitrogen 5.0, *Linde* (http://www.linde-gas.de), ≥ 99.999 % N_2). Analytical TLC was carried out using pre-coated silica gel plates (*Merck* (http://www.merck.de) TLC plates silica gel $60F_{254}$). Flash column chromatography was performed using silica gel (particle size 40-63 mM, *Acros Organics (http://www.acros.com)*). Pressure was applied with N_2 at 1 bar (Nitrogen 5.0, *Linde* (http://www.linde-gas.de), ≥ 99.999 % N_2) All sensitive reactions were carried out in flame dried glassware under an argon atmosphere unless otherwise noted. Chemicals were purchased from following suppliers and used without further purification, unless otherwise noted. Deutero Germany (http://www.deutero.de): D_2O, acetone d_6; Sigma Aldrich (https://www.sigmaaldrich.com): CD_3I (99.5%), $BF_3 \cdot THF$, quercetin (>95%), naringenin (98%); Acros Organics (http://www.acros.com): DMF (extra dry, over molar sieves), Acetylchloride (98%), MeOH (HPLC-grade). Apollo Scientific

8http://www.apolloscientific.co.uk): Phosphorous Pentoxide (98%). Cyclohexane and Ethyl acetate (both tech grade) were distilled prior to use.

Synthesis of D_3-Quercetin

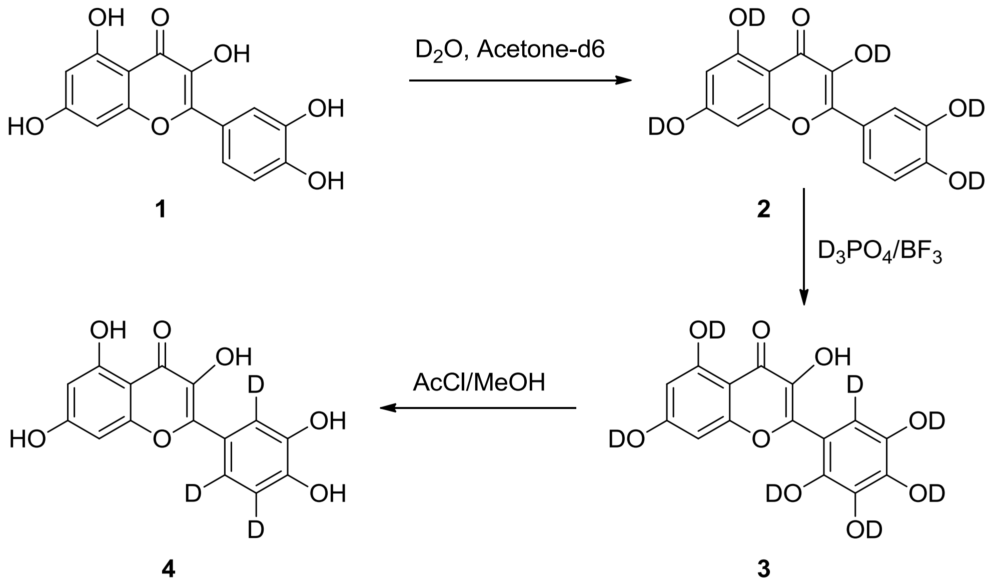

200 mg (0.66 mmol, 1.0 equiv.) of quercetin were dissolved in 2 ml D_2O and 2 ml acetone-D_6 and heated to 50 °C in a pressure Schlenk tube. After 30 min all volatiles were removed carefully under line vacuum to yield a yellow solid **2**. In a separate Schlenk tube 3.0 g (22 mmol) P_2O_5 were carefully treated with 3 ml D_2O at 0 °C. After complete addition the mixture was warmed to room temperature and stirred for further 20 min. 12.2 ml (110 mmol, 5 equiv.) $BF_3 \cdot THF$ was added and this mixture was carefully transferred into the pressure Schlenk tube. The mixture was heated in the closed tube under argon atmosphere for 2d at 55 °C. After cooling to room temperature the mixture was poured into ice water and was filtrated. The precipitate was dissolved in 40 ml of 1% AcCl in MeOH and refluxed for 60 min. After removing all volatiles under reduced pressure about 180 mg (90%) of D_3-Quercetin were isolated.

The above described procedure was repeated 2 times and the final product was recrystallized from methanol to yield about 80 mg of the D_3-Quercetin **4** with a D_3-content of 81% according to ESI-MS analytics (see below).

Synthesis of D3-Sakuranetin

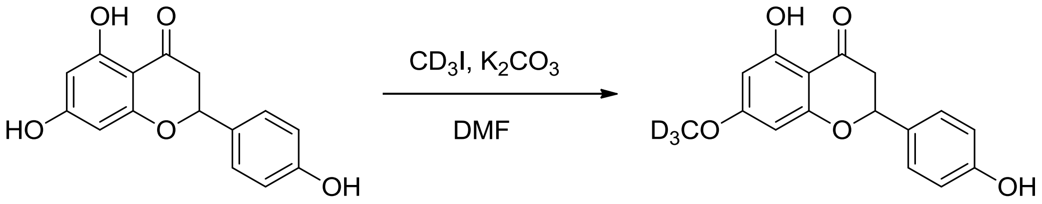

For the synthesis of D_3-Sakuranetin, 200 mg (0.73 mmol, 1.0 equiv.) naringenin and 100 mg (0.73 mmol, 1.0 equiv.) K_2CO_3 were solved in 2.4 ml anhydrous DMF. While stirring, 68 µl (1.1 mmol, 1.5 equiv.) CD_3I were added gradually. The solution was stirred for 15h at room temperature, was hydrolyzed with 1N HCl and extracted two times with EtOAc. The combined organic extracts were washed with an aqueous saturated NaCl-solution and dried over $MgSO_4$. After evaporation of the solvent under reduced pressure the residue was purified via flash column chromatography eluting with cyclohexane/EtOAc 2:1 yielding 149 mg (0.52 mmol, 71%) of the mono-methylated product as colorless solid which is in agreement with the previous methylation of naringenin (Oyama and Kondo, 2004).

$C_{16}H_{11}D_3O_5$, M = 289.30 g/mol

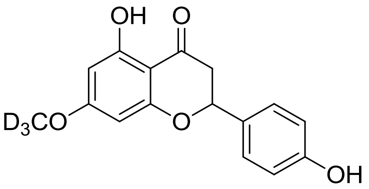

R_f (SiO$_2$, cHex/EtOAc 2:1) = 0.39

FT-IR (ATR) $\tilde{\nu}$ [cm^{-1}] = 3971 (w), 3927 (w), 3860 (w), 3820 (w), 3796 (w), 3726 (w), 3693 (w), 3667 (w), 3476 (w), 3279 (w), 2066 (w), 1634 (ss), 1570 (s), 1517 (s), 1447 (m), 1373 (m), 1340 (m), 1307 (m), 1270 (m), 1193 (s), 1179 (s), 1100 (s), 1070 (m), 906 (w), 880 (w), 832 (m), 736 (m).

^{1}H NMR (300 MHz, $CDCl_3$) : δ (ppm) = 12.02 (s, 1H, OH); 7.33 (d, J = 8.5 Hz, 2H); 6.89 (d, J = 8.5 Hz, 2H); 6.07 (d, J = 2.3 Hz, 1H); 6.04 (d, J = 2.3 Hz, 1H); 5.35 (dd, ^3J = 13.1 Hz, J = 3.0 Hz, 1H); 3.09 (dd, J = 13.1 Hz, J = 17.2 Hz); 2.79 (dd, J = 17.2 Hz, J = 3.0 Hz, 1H).

One OH-group could not be detected.

^{13}C NMR (125 MHz, $CDCl_3$) : δ (ppm) = 196.1, 164.1, 162.9, 156.2, 130.5, 128.0, 115.7, 103.1, 95.1, 94.3, 79.0, 43.2.

**HR-MS**	calcd. mass [u]	measured mass [u]	error [ppm]
**(ESI)**	m/z [M+H] = 290.1102303	m/z [M+H] = 290.11034	+0.38
	m/z [M+Na] = 312.0921750	m/z [M+Na] = 312.09245	+0.87

ESI-MS analytics of final products

ESI-MS analytics of final products were executed with 30 µM per substance using a Thermo Exactive Orbitrap MS (http://www.thermoscientific.com) operated in negative ion mode with a spray voltage of 4.2 kV; sheath gas and auxiliary nitrogen pressures at 10 and 0 respective arbitrary units and capillary temperature at 275°C at maximum mass resolution. Spectra were averaged across the peak and deuteration grades were calculated using the relative abundance.

ESI-MS analytics for D_3-quercetin using a Thermo Exactive Orbitrap MS

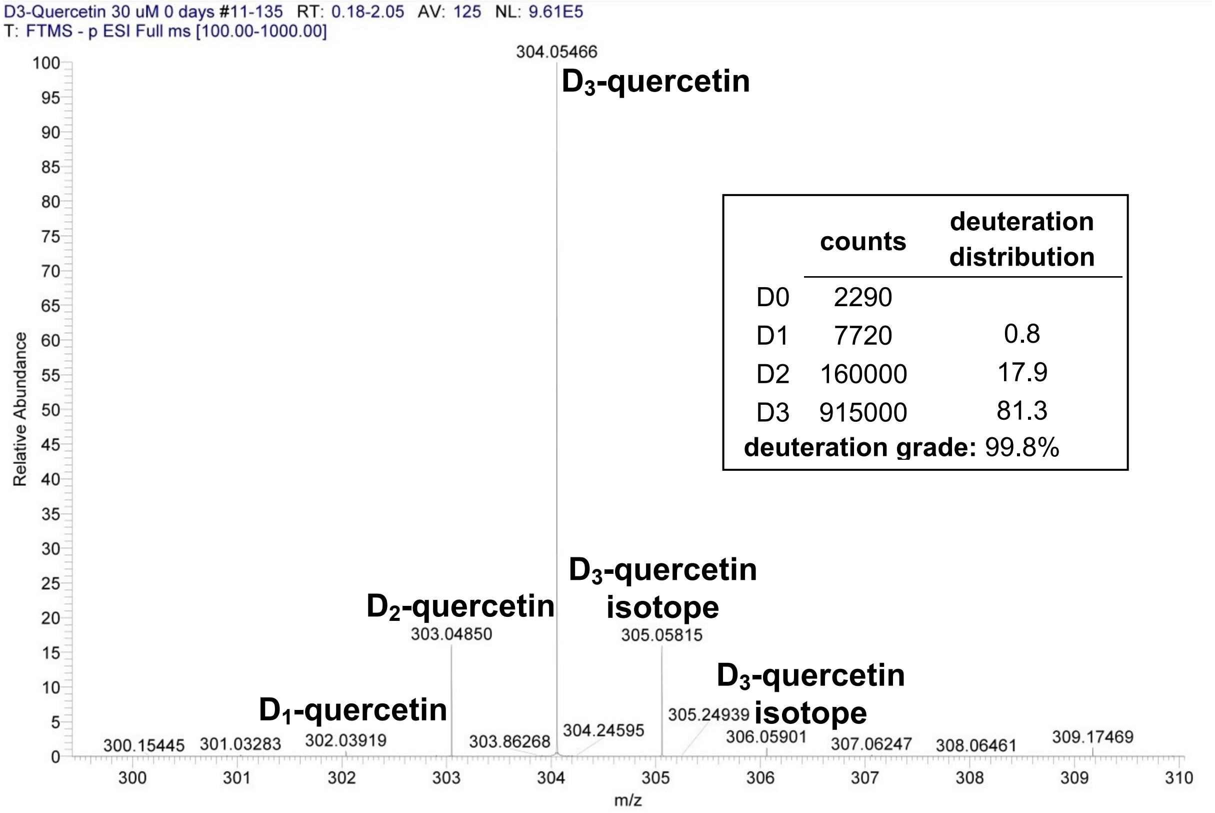

	counts	deuteration distribution
D0	2290	
D1	7720	0.8
D2	160000	17.9
D3	915000	81.3
**deuteration grade:** 99.8%		

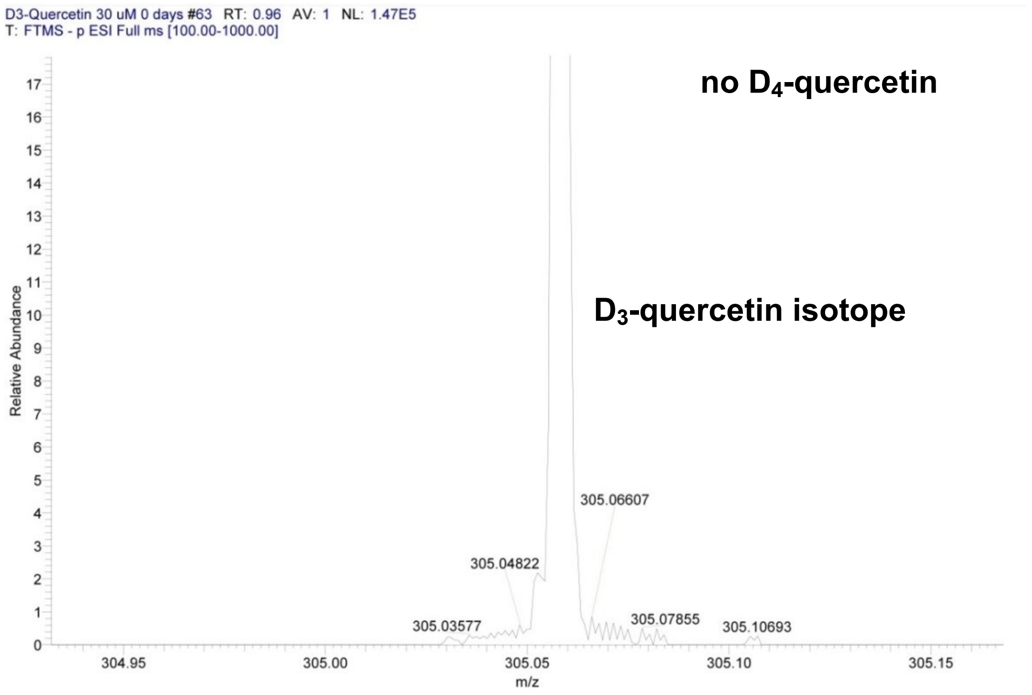

ESI-MS analytics for D_3-sakuranetin using a Thermo Exactive Orbitrap MS

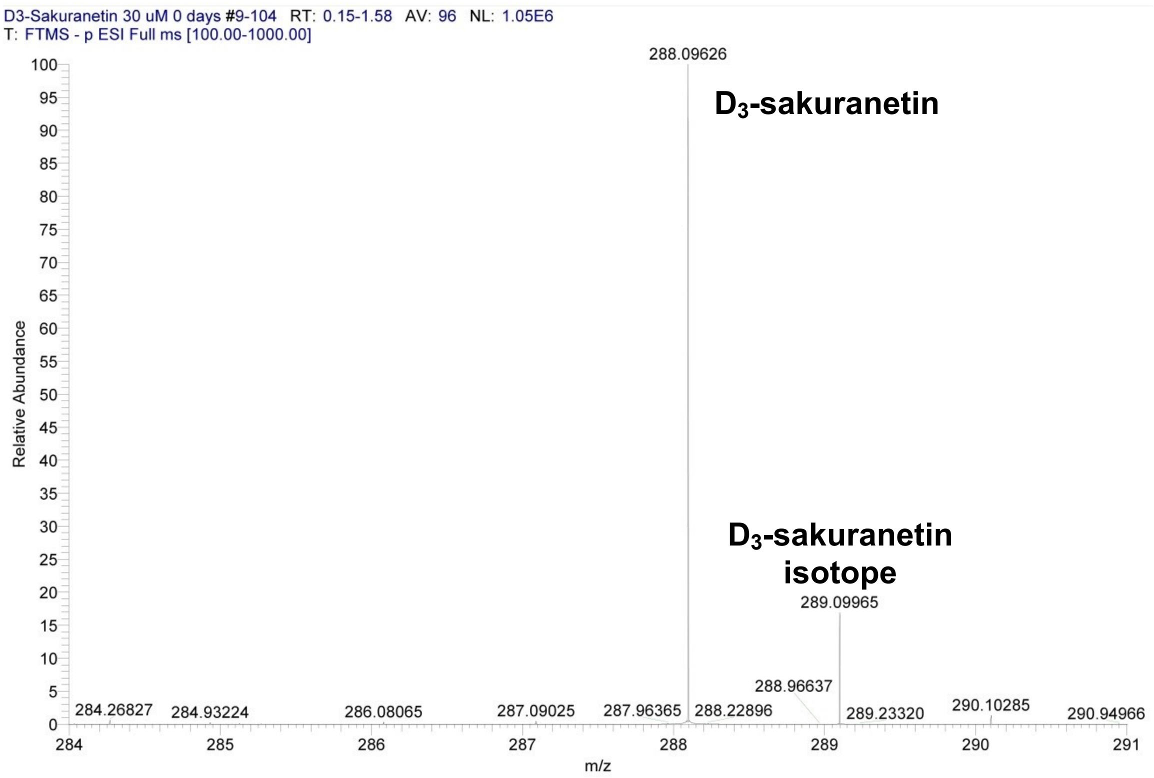

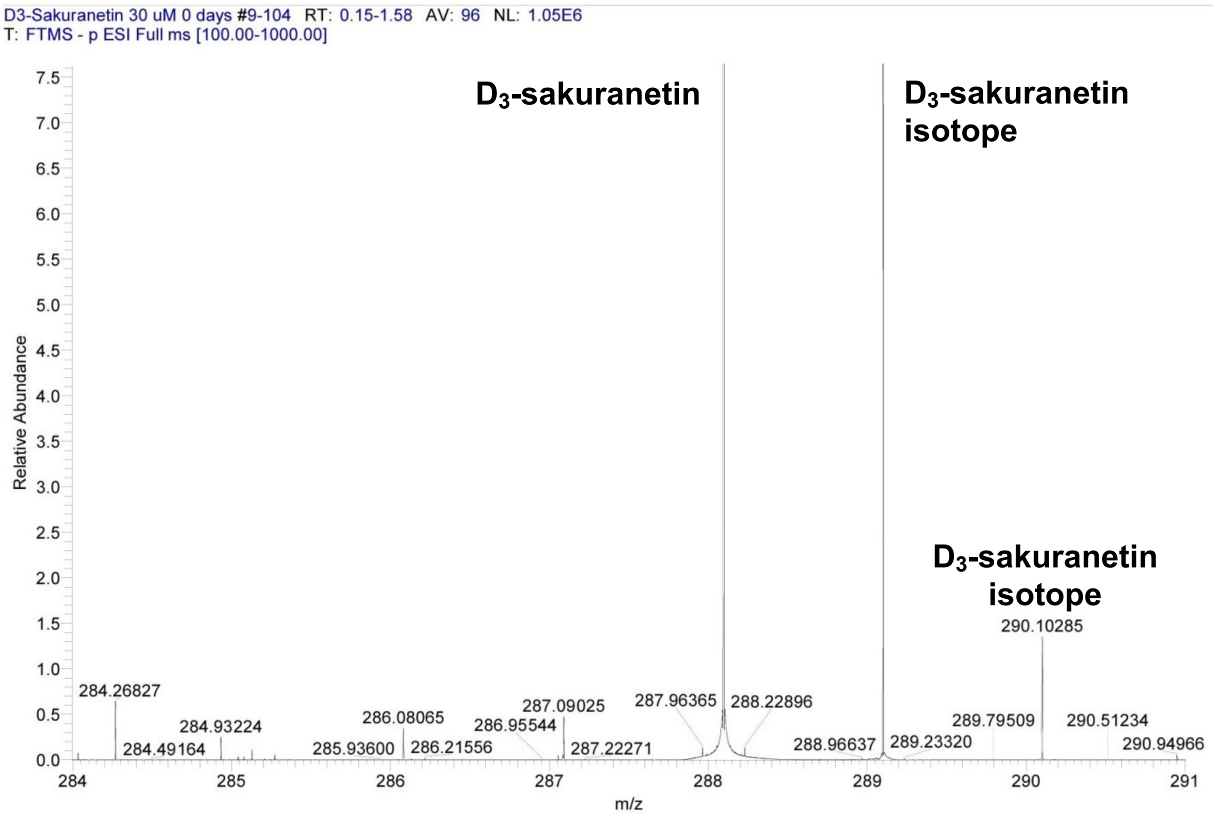

D3-Sakuranetin 30 uM 0 days #9-104 RT: 0.15-1.58 AV: 96 NL: 1.05E6
T: FTMS - p ESI Full ms [100.00-1000.00]
D3-sakuranetin
D3-sakuranetin isotope
D3-sakuranetin isotope
290.10285
Relative Abundance
m/z
284.26827
284.49164
284.93224
285.93600
286.08065
286.21556
286.95544
287.09025
287.22271
287.96365
288.22896
288.96637
289.23320
289.79509
290.51234
290.94966
