# Supplementary material for: A fast and simple LC-MS-based characterization of the flavonoid biosynthesis pathway for few seed(ling)s
Source: BMC Plant Biol. 2016 Sep 1;16(1):190. doi: 10.1186/s12870-016-0880-7 (PMC5007998; doi:10.1186/s12870-016-0880-7)
Supplement: Additional file 4: Figure S4. — Extraction and hydrolysis time. Selection of the methanol concentration; Figure S5. Non-adjusted responses from Figure S4; Figure S6. Seed extracts treated with different hydrolysis times; Table S9. Data for Figures S4, S5; and Table S10. Data for Fig. 2 and Figure S6. (PDF 1210 kb) [file 12870_2016_880_MOESM4_ESM.pdf]

Additional_file_4

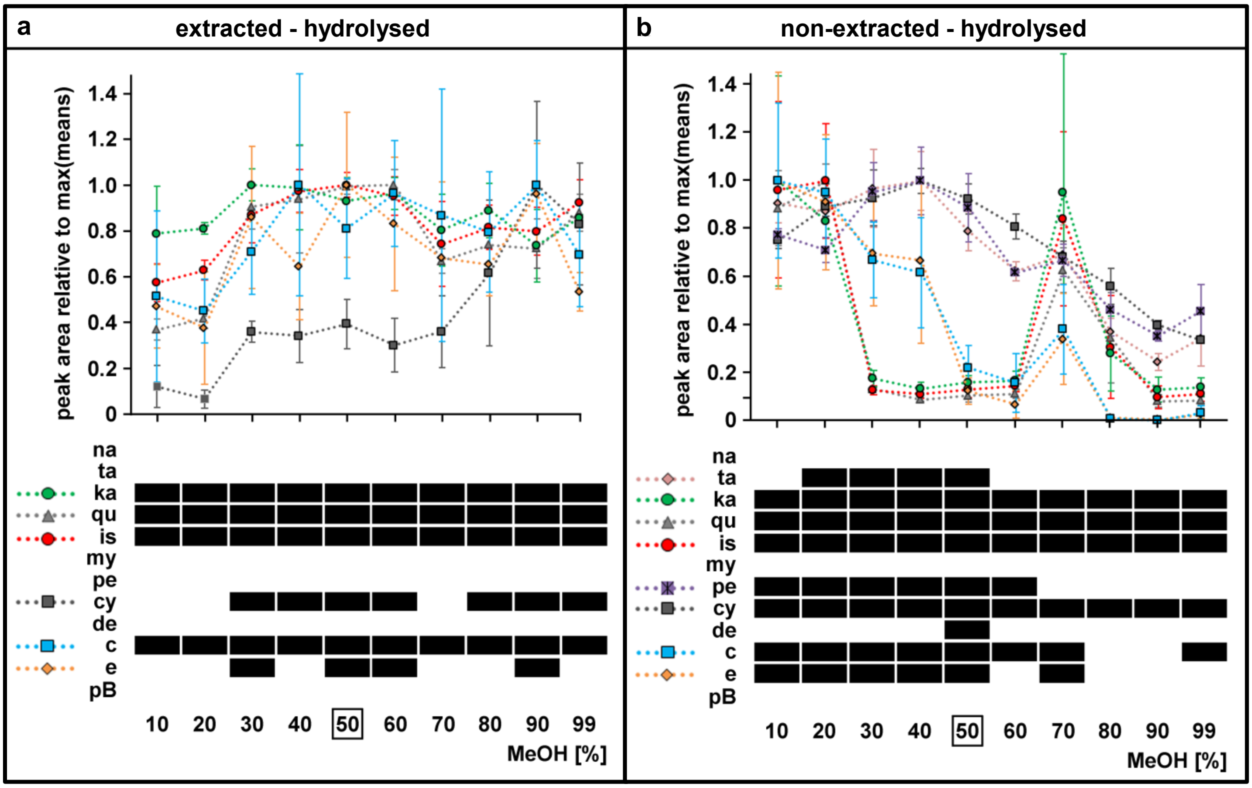

Figure S4: Selection of the methanol concentration.
LC-ESI-MS-QTRAP(MRM)-responses of flavonoids from the selected set for extracted hydrolysed (a) and non-extracted hydrolyzed (b) samples. For comparison, each response was set relative to the maximal mean per substance through all tested MeOH concentrations. Black boxes below the plots indicate when the limit of quantification (LOQ) was passed for the majority of respective replicates. Boxed: selected MeOH concentration. Note, that substances from the same class share a similar extraction behaviour. A different concentration can be chosen for specific purpose. Interrupted lines serve for visualization. n=3 Error bars = STDEV. (See Figure S5 for soluble samples and non-adjusted responses and Table S7 for the underlying data).

na: naringenin, ta: taxifolin, ka: kaempferol, qu: quercetin, is: isorhamnetin, my: myricetin, pe: pelargonidin, cy: cyanidin, de: delphinidin, c: catechin, e: epicatechin, pB: procyanidinB2.

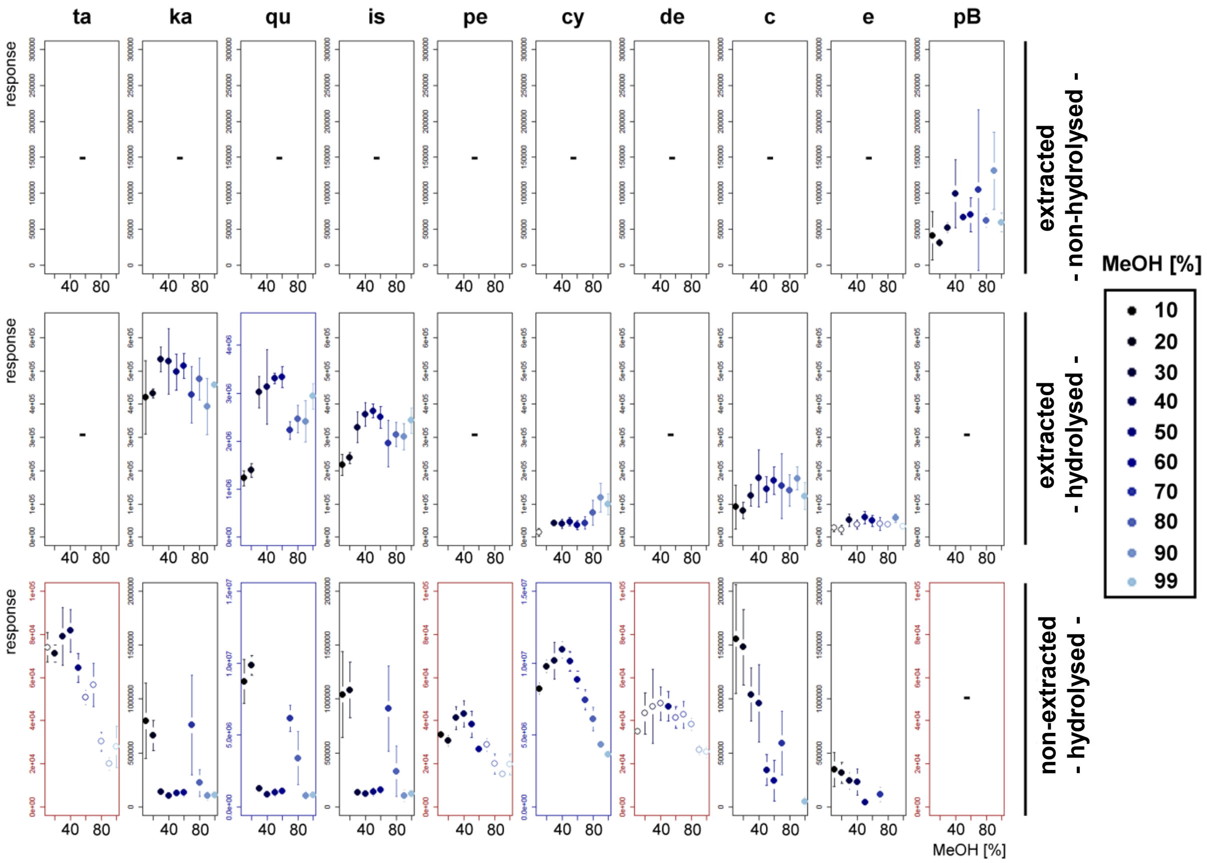

Figure S5: Non-adjusted responses from Fig. S4. Shown are non-adjusted responses for the ten detected substances corresponding to the data presented in Fig. S4 for extracted non-hydrolysed, extracted hydrolysed and non-extracted hydrolysed samples. The different MeOH concentrations in the solvent are indicated through a color code. Note, in each line, plots of the same scale are boxed in the same color. n=3. Error bars = STDEV. See Table S7 for the underlying data.

ta: taxifolin, ka: kaempferol, qu: quercetin, is: isorhamnetin, my: myricetin, pe: pelargonidin, cy: cyanidin, de: delphinidin, c: catechin, e: epicatechin, pB: procyanidinB2.

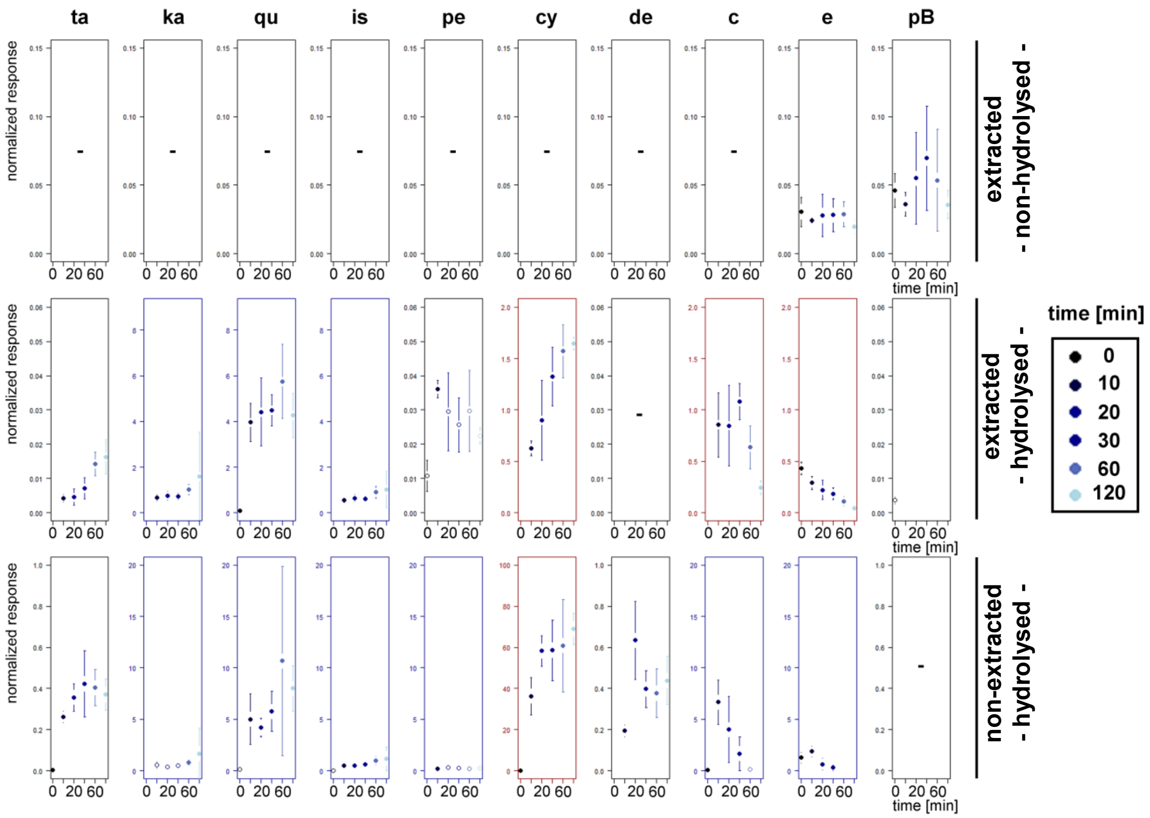

Figure S6: Seed extracts treated with different hydrolysis times.
Shown are responses normalized to the respective internal standard for the different indicated extracts and treatments. The different hydrolysis times are indicated as a color code. Extracted non-hydrolysed samples with the indicated time are not hydrolysed but correspond to the same initial seeds as the respective mean at this time for the hydrolysed samples. 10 seeds were used per sample in five replicates. Note, in each line, plots of the same scale are boxed in the same color. Filled dots: above LOQ. Empty dots: above LOD but below LOQ. Error bars = STDEV. (See Table S10 for the underlying data)

ta: taxifolin, ka: kaempferol, qu: quercetin, is: isorhamnetin, my: myricetin, pe: pelargonidin, cy: cyanidin, de: delphinidin, c: catechin, e: epicatechin, pB: procyanidinB2.

Table S9. Data for Fig. S4,S5

Figure	MeOH [%]	Sample type	Substance	LOD passed	LOQ passed	response	STDEV	RSD [%]
S5	10	extracted, non-hydrolysed	pB2	+	+	4.15E+04	3.37E+04	81.17
	20		pB2	+	+	3.16E+04	4.66E+03	14.73
	30		pB2	+	+	5.24E+04	6.67E+03	12.74
	40		pB2	+	+	9.95E+04	4.76E+04	47.84
	50		pB2	+	+	6.69E+04	3.07E+03	4.60
	60		pB2	+	+	7.00E+04	2.35E+04	33.62
	70		pB2	+	+	1.05E+05	1.11E+05	106.56
	80		pB2	+	+	6.21E+04	9.35E+03	15.06
	90		pB2	+	+	1.31E+05	5.39E+04	41.00
	99		pB2	+	+	5.95E+04	1.33E+04	22.30
S4a,S5	10	extracted, hydrolysed	ka	+	+	4.21E+05	1.11E+05	26.24
			qu	+	+	1.23E+06	1.56E+05	12.70
			is	+	+	2.18E+05	3.17E+04	14.55
			cy	+	-	1.44E+04	1.11E+04	77.01
			c	+	+	9.10E+04	6.63E+04	72.79
			e	+	-	2.79E+04	1.08E+04	38.77
S4a,S5	20	extracted, hydrolysed	ka	+	+	4.33E+05	1.37E+04	3.15
			qu	+	+	1.39E+06	1.46E+05	10.51
			is	+	+	2.38E+05	1.63E+04	6.83
			cy	-	-	n.d.	n.d.	n.d.
			c	+	+	7.98E+04	2.48E+04	31.09
			e	+	-	2.24E+04	1.46E+04	65.32
S4a,S5	30	extracted, hydrolysed	ka	+	+	5.35E+05	3.77E+04	7.05
			qu	+	+	3.02E+06	3.29E+05	10.90
			is	+	+	3.30E+05	4.66E+04	14.11
			cy	+	+	4.27E+04	5.40E+03	12.66
			c	+	+	1.26E+05	3.29E+04	26.17
			e	+	+	5.10E+04	1.84E+04	36.10
S4a,S5	40	extracted, hydrolysed	ka	+	+	5.29E+05	9.83E+04	18.59
			qu	+	+	3.13E+06	7.82E+05	24.98
			is	+	+	3.69E+05	3.51E+04	9.50
			cy	+	+	4.05E+04	1.37E+04	33.88
			c	+	+	1.77E+05	8.58E+04	48.40
			e	+	-	3.82E+04	1.39E+04	36.23

Table S9. Data for Fig. S4,S5

Figure	MeOH [%]	Sample type	Substance	LOD passed	LOQ passed	response	STDEV	RSD [%]
S4a,S5	50	extracted, hydrolysed	ka	+	+	4.97E+05	5.46E+04	11.00
			qu	+	+	3.31E+06	1.10E+05	3.31
			is	+	+	3.80E+05	2.07E+04	5.46
			cy	+	+	4.68E+04	1.28E+04	27.30
			c	+	+	1.44E+05	3.86E+04	26.89
			e	+	+	5.95E+04	1.88E+04	31.69
S4a,S5	60	extracted, hydrolysed	ka	+	+	5.15E+05	3.72E+04	7.23
			qu	+	+	3.34E+06	2.17E+05	6.51
			is	+	+	3.61E+05	3.19E+04	8.84
			cy	+	+	3.57E+04	1.40E+04	39.08
			c	+	+	1.71E+05	4.11E+04	24.06
			e	+	+	4.93E+04	1.73E+04	35.14
S4a,S5	70	extracted, hydrolysed	ka	+	+	4.29E+05	8.45E+04	19.71
			qu	+	+	2.22E+06	1.80E+05	8.08
			is	+	+	2.82E+05	6.98E+04	24.78
			cy	+	+	4.28E+04	1.86E+04	43.52
			c	+	+	1.54E+05	9.76E+04	63.48
			e	+	-	4.05E+04	1.96E+04	48.43
S4a,S5	80	extracted, hydrolysed	ka	+	+	4.75E+05	6.30E+04	13.27
			qu	+	+	2.46E+06	2.86E+05	11.60
			is	+	+	3.09E+05	3.68E+04	11.90
			cy	+	+	7.32E+04	3.78E+04	51.63
			c	+	+	1.41E+05	4.66E+04	33.11
			e	+	-	3.88E+04	8.21E+03	21.15
S4a,S5	90	extracted, hydrolysed	ka	+	+	3.93E+05	8.48E+04	21.57
			qu	+	+	2.41E+06	4.33E+05	17.96
			is	+	+	3.03E+05	3.92E+04	12.97
			cy	+	+	1.19E+05	4.33E+04	36.37
			c	+	+	1.77E+05	3.46E+04	19.55
			e	+	+	5.71E+04	1.31E+04	22.88
S4a,S5	99	extracted, hydrolysed	ka	+	+	4.58E+05	6.56E+03	1.43
			qu	+	+	2.93E+06	2.71E+05	9.25
			is	+	+	3.51E+05	3.79E+04	10.80
			cy	+	+	9.86E+04	3.17E+04	32.17
			c	+	+	1.23E+05	4.03E+04	32.73
			e	+	-	3.17E+04	4.93E+03	15.54

Table S9. Data for Fig. S4,S5

Figure	MeOH [%]	Sample type	Substance	LOD passed	LOQ passed	response	STDEV	RSD [%]
S4b,S5	10	non-extracted, hydrolysed	ta	+	-	7.39E+04	6.77E+03	9.15
			ka	+	+	7.97E+05	3.49E+05	43.78
			qu	+	+	8.72E+06	1.53E+06	17.55
			is	+	+	1.04E+06	3.96E+05	38.03
			pe	+	+	3.35E+04	1.10E+03	3.29
			cy	+	+	8.22E+06	3.79E+05	4.61
			de	+	-	3.51E+04	1.12E+03	3.18
			c	+	+	1.56E+06	5.03E+05	32.33
			e	+	+	3.48E+05	1.57E+05	45.16
S4b,S5	20	non-extracted, hydrolysed	ta	+	+	7.11E+04	3.90E+03	5.48
			ka	+	+	6.64E+05	1.39E+05	20.86
			qu	+	+	9.84E+06	6.91E+05	7.02
			is	+	+	1.08E+06	2.58E+05	23.85
			pe	+	+	3.07E+04	2.31E+03	7.51
			cy	+	+	9.76E+06	4.35E+05	4.46
			de	+	-	4.34E+04	9.49E+03	21.88
			c	+	+	1.48E+06	3.47E+05	23.42
			e	+	+	3.17E+05	9.82E+04	31.01
S4b,S5	30	non-extracted, hydrolysed	ta	+	+	7.90E+04	1.33E+04	16.83
			ka	+	+	1.40E+05	2.61E+04	18.60
			qu	+	+	1.30E+06	5.03E+04	3.86
			is	+	+	1.38E+05	2.04E+04	14.80
			pe	+	+	4.12E+04	5.20E+03	12.61
			cy	+	+	1.02E+07	1.28E+06	12.58
			de	+	-	4.66E+04	1.71E+04	36.74
			c	+	+	1.04E+06	2.46E+05	23.59
			e	+	+	2.42E+05	7.57E+04	31.22
S4b,S5	40	non-extracted, hydrolysed	ta	+	+	8.16E+04	9.98E+03	12.23
			ka	+	+	1.06E+05	2.07E+04	19.52
			qu	+	+	8.58E+05	7.14E+04	8.32
			is	+	+	1.20E+05	1.89E+04	15.73
			pe	+	+	4.32E+04	6.13E+03	14.19
			cy	+	+	1.09E+07	5.69E+05	5.20
			de	+	-	4.80E+04	7.74E+03	16.14
			c	+	+	9.60E+05	3.57E+05	37.21
			e	+	+	2.32E+05	1.20E+05	51.56

Table S9. Data for Fig. S4,S5

Figure	MeOH [%]	Sample type	Substance	LOD passed	LOQ passed	response	STDEV	RSD [%]
S4b,S5	50	non-extracted, hydrolysed	ta	+	+	6.45E+04	6.60E+03	10.24
			ka	+	+	1.27E+05	2.20E+04	17.34
			qu	+	+	1.03E+06	2.64E+05	25.69
			is	+	+	1.41E+05	3.61E+04	25.67
			pe	+	+	3.84E+04	6.15E+03	16.03
			cy	+	+	1.01E+07	6.88E+05	6.80
			de	+	+	4.66E+04	6.85E+03	14.71
			c	+	+	3.45E+05	1.43E+05	41.44
			e	+	+	4.32E+04	1.93E+04	44.79
S4b,S5	60	non-extracted, hydrolysed	ta	+	-	5.08E+04	3.33E+03	6.55
			ka	+	+	1.33E+05	3.21E+04	24.10
			qu	+	+	1.10E+06	1.81E+05	16.50
			is	+	+	1.58E+05	2.89E+04	18.34
			pe	+	+	2.67E+04	4.93E+02	1.85
			cy	+	+	8.85E+06	5.81E+05	6.56
			de	+	-	4.15E+04	5.06E+03	12.18
			c	+	+	2.45E+05	1.90E+05	77.49
			e	-	-	n.d.	n.d.	n.d.
S4b,S5	70	non-extracted, hydrolysed	ta	+	-	5.65E+04	1.00E+04	17.79
			ka	+	+	7.59E+05	4.60E+05	60.52
			qu	+	+	6.16E+06	9.17E+05	14.87
			is	+	+	9.11E+05	3.94E+05	43.25
			pe	+	-	2.89E+04	2.93E+03	10.14
			cy	+	+	7.45E+06	7.20E+05	9.66
			de	+	-	4.28E+04	6.16E+03	14.38
			c	+	+	5.93E+05	2.92E+05	49.30
			e	+	+	1.18E+05	6.63E+04	56.02
S4b,S5	80	non-extracted, hydrolysed	ta	+	-	3.03E+04	4.49E+03	14.83
			ka	+	+	2.24E+05	1.24E+05	55.62
			qu	+	+	3.40E+06	1.85E+06	54.31
			is	+	+	3.32E+05	2.31E+05	69.50
			pe	+	-	2.00E+04	4.73E+03	23.67
			cy	+	+	6.13E+06	8.18E+05	13.35
			de	+	-	3.83E+04	2.61E+03	6.80
			c	-	-	n.d.	n.d.	n.d.
			e	-	-	n.d.	n.d.	n.d.

Table S9. Data for Fig. S4,S5

Figure	MeOH [%]	Sample type	Substance	LOD passed	LOQ passed	response	STDEV	RSD [%]
S4b,S5	90	non-extracted, hydrolysed	ta	+	-	2.00E+04	2.92E+03	14.60
			ka	+	+	1.03E+05	4.11E+04	39.89
			qu	+	+	7.97E+05	2.58E+05	32.43
			is	+	+	1.06E+05	5.25E+04	49.59
			pe	+	-	1.52E+04	9.07E+02	5.96
			cy	+	+	4.36E+06	1.95E+05	4.48
			de	+	-	2.65E+04	2.15E+03	8.12
			c	-	-	n.d.	n.d.	n.d.
			e	-	-	n.d.	n.d.	n.d.
S4b,S5	99	non-extracted, hydrolysed	ta	+	-	2.80E+04	9.52E+03	34.03
			ka	+	+	1.11E+05	3.09E+04	27.76
			qu	+	+	8.27E+05	1.42E+05	17.21
			is	+	+	1.21E+05	3.49E+04	28.95
			pe	+	-	1.97E+04	4.82E+03	24.43
			cy	+	+	3.66E+06	1.35E+05	3.69
			de	+	-	2.54E+04	2.69E+03	10.56
			c	+	+	5.08E+04	4.62E+04	90.93
			e	-	-	n.d.	n.d.	n.d.

For experimental procedures see methods, text and Fig. S4 and Fig. S5. The number of replicates is three. Note that for each samples type, only data is shown for substances for which at least on MeOH concentration was above LOQ. “n.d.” = not determined., “ta” = taxifolin, “ka” = kaempferol, “qu” = quercetin, “is” = isorhamnetin, “pe” = pelargonidin, “cy” = cyanidin, “de” = delphinidin, “c”= catechin, “e”=epicatechin, “pB2” = procyanidin B2. For LOD/LOQ see methods. Responses below LOQ were only used for plotting in Fig. S5 but not for drawing the decision.

Table S10. Data for Fig. 2 and Figure S6

Figure	Hydrolysis [min]	Sample type	Substance	LOD passed	LOQ passed	Normalized response	STDEV	RSD [%]	Compared to	*P*-value
S6	0	extracted non-hydrolysed	e	+	+	0.031	0.011	35.08	n.d.	n.d.
S6	0	extracted non-hydrolysed	pB2	+	+	0.046	0.012	26.30	n.d.	n.d.
S6	10	extracted non-hydrolysed	e	+	+	0.024	0.002	8.36	n.d.	n.d.
S6	10	extracted non-hydrolysed	pB2	+	+	0.036	0.009	23.93	n.d.	n.d.
S6	20	extracted non-hydrolysed	e	+	+	0.028	0.016	55.60	n.d.	n.d.
S6	20	extracted non-hydrolysed	pB2	+	+	0.055	0.034	60.92	n.d.	n.d.
S6	30	extracted non-hydrolysed	e	+	-	0.028	0.012	42.51	n.d.	n.d.
S6	30	extracted non-hydrolysed	pB2	+	+	0.070	0.038	54.46	n.d.	n.d.
S6	60	extracted non-hydrolysed	e	+	+	0.029	0.009	31.60	n.d.	n.d.
S6	60	extracted non-hydrolysed	pB2	+	+	0.054	0.037	69.11	n.d.	n.d.
S6	120	extracted non-hydrolysed	e	+	+	0.019	0.005	24.32	n.d.	n.d.
S6	120	extracted non-hydrolysed	pB2	+	+	0.036	0.010	27.94	n.d.	n.d.
2, S6	0	extracted hydrolysed	ta	-	-	n.d.	n.d.	n.d.	n.d.	n.d.
2, S6	0	extracted hydrolysed	ka	-	-	n.d.	n.d.	n.d.	n.d.	n.d.
2, S6	0	extracted hydrolysed	qu	+	+	0.092	0.031	33.85	10/20/30/60/120	<0.01
2, S6	0	extracted hydrolysed	is	-	-	n.d.	n.d.	n.d.	n.d.	n.d.
2, S6	0	extracted hydrolysed	pe	+	-	0.011	0.005	42.68	10/20/30/60/120	<0.05
2, S6	0	extracted hydrolysed	cy	-	-	n.d.	n.d.	n.d.	n.d.	n.d.
2, S6	0	extracted hydrolysed	c	-	-	n.d.	n.d.	n.d.	n.d.	n.d.
2, S6	0	extracted hydrolysed	e	+	+	0.433	0.061	13.99	10/20/30/60/120	<0.01
2, S6	0	extracted hydrolysed	pB	+	-	0.004	0.001	28.66	n.d.	n.d.
2, S6	10	extracted hydrolysed	ta	+	+	0.004	0.001	26.00	60/120*	<0.01
2, S6	10	extracted hydrolysed	ka	+	+	0.676	0.138	20.47	60*	<0.05
2, S6	10	extracted hydrolysed	qu	+	+	3.955	0.828	20.93	0*	<0.001
2, S6	10	extracted hydrolysed	is	+	+	0.548	0.093	16.89	60*	<0.05
2, S6	10	extracted hydrolysed	pe	+	+	0.036	0.003	7.18	0/30/120*	<0.05
2, S6	10	extracted hydrolysed	cy	+	+	0.628	0.073	11.70	30/60/120*	<0.01
2, S6	10	extracted hydrolysed	c	+	+	0.855	0.314	36.69	120*	<0.05
2, S6	10	extracted hydrolysed	e	+	+	0.291	0.066	22.54	0/30/60/120*	<0.05
2, S6	10	extracted hydrolysed	pB	-	-	n.d.	n.d.	n.d.	n.d.	n.d.

Table S10. Data for Fig. 2 and Figure S6

Figure	Hydrolysis [min]	Sample type	Substance	LOD passed	LOQ passed	Normalized response	STDEV	RSD [%]	Compared to	*P*-value
2, S6	20	extracted hydrolysed	ta	+	+	0.005	0.002	52.42	60/120*	<0.01
			ka	+	+	0.741	0.186	25.12	60*	<0.05
			qu	+	+	4.413	1.495	33.88	0*	<0.01
			is	+	+	0.645	0.154	23.87	all	>0.05
			pe	+	-	0.029	0.011	38.65	0*	<0.05
			cy	+	+	0.900	0.389	43.29	60/120*	<0.05
			c	+	+	0.848	0.394	46.44	120*	<0.05
			e	+	+	0.221	0.094	42.64	0/120*	<0.05
			pB	-	-	n.d	n.d	n.d	n.d	n.d
2, S6	30	extracted hydrolysed	ta	+	+	0.007	0.003	42.77	60/120*	<0.05
			ka	+	+	0.721	0.144	19.95	30*	<0.05
			qu	+	+	4.485	0.688	15.34	0*	<0.001
			is	+	+	0.620	0.097	15.62	all	>0.05
			pe	+	-	0.026	0.008	31.14	0/10*	<0.05
			cy	+	+	1.325	0.286	21.56	10*	<0.01
			c	+	+	1.081	0.175	16.19	60/120*	<0.01
			e	+	+	0.187	0.057	30.32	0/10/120*	<0.05
			pB	-	-	n.d	n.d	n.d	n.d.	n.d.
2, S6	60	extracted hydrolysed	ta	+	+	0.014	0.003	23.79	10/20/30*	<0.01
			ka	+	+	1.028	0.221	21.44	10/30*	<0.05
			qu	+	+	5.752	1.615	28.09	0*	<0.001
			is	+	+	0.896	0.259	28.87	10*	<0.05
			pe	+	-	0.030	0.012	39.62	0*	<0.05
			cy	+	+	1.571	0.260	16.53	10/20*	<0.05
			c	+	+	0.637	0.211	33.18	30/120*	<0.05
			e	+	+	0.113	0.044	38.71	0/10/120*	<0.05
			pB	-	-	n.d	n.d	n.d	n.d.	n.d
2, S6	120	extracted hydrolysed	ta	+	+	0.016	0.005	30.17	10/20/30*	<0.05
			ka	+	+	1.603	1.933	120.62	all	>0.05
			qu	+	+	4.260	0.965	22.64	0*	<0.001
			is	+	+	1.021	0.796	77.93	all	>0.05
			pe	+	-	0.022	0.002	10.52	0/10*	<0.01
			cy	+	+	1.649	0.061	3.71	10/20*	<0.05
			c	+	+	0.244	0.066	27.03	10/20/30/60	<0.05
			e	+	+	0.042	0.013	31.94	0/10/20/30/60	<0.05
			pB	-	-	n.d.	n.d.	n.d	n.d.	n.d

Table S10. Data for Fig. 2 and Figure S6

Figure	Hydrolysis [min]	Sample type	Substance	LOD passed	LOQ passed	Normalized response	STDEV	RSD [%]	Compared to	*P*-value
2, S6	0	non-extracted hydrolysed	ta	+	+	0.003	0.002	79.15	0/10/20/30/60/120	<0.01
			ka	-	-	n.d	n.d	n.d	n.d.	n.d
			qu	+	-	0.148	0.055	37.00	10/20/30/120*	<0.05
			is	+	-	0.023	0.007	29.02	10/20/30/60*	<0.01
			pe	-	-	n.d	n.d	n.d	n.d.	n.d.
			cy	+	+	0.085	0.035	40.84	10/20/30/60/120	<0.01
			de	-	-	n.d.	n.d.	n.d	n.d.	n.d.
			c	+	+	0.101	0.033	32.37	10*	<0.01
			e	+	+	1.268	0.525	41.41	30*	<0.05
2, S6	10	non-extracted hydrolysed	ta	+	+	0.262	0.026	10.03	0/20/60/120*	<0.05
			ka	+	-	0.573	0.296	51.63	all	>0.05
			qu	+	+	5.019	2.440	48.60	0*	<0.05
			is	+	+	0.477	0.155	32.43	0/60*	<0.05
			pe	+	+	0.207	0.067	32.34	20*	<0.05
			cy	+	+	36.306	9.108	25.09	0/20/30/120*	<0.05
			de	+	+	0.194	0.028	14.58	20/30/60/120	<0.05
			c	+	+	6.680	2.154	32.24	0/30/60*	<0.01
			e	+	+	1.873	0.498	26.57	20/30*	<0.01
2, S6	20	non-extracted hydrolysed	ta	+	+	0.357	0.067	18.68	0/10*	<0.05
			ka	+	-	0.374	0.076	20.20	60*	<0.05
			qu	+	+	4.197	0.906	21.59	0*	<0.001
			is	+	+	0.505	0.044	8.77	0*	<0.001
			pe	+	+	0.318	0.059	18.40	10/60/120*	<0.05
			cy	+	+	58.212	7.330	12.59	0/10*	<0.01
			de	+	+	0.634	0.191	30.08	10/30/60*	<0.05
			c	+	+	4.009	3.211	80.10	all	>0.05
			e	+	+	0.644	0.561	87.06	10*	<0.01
2, S6	30	non-extracted hydrolysed	ta	+	+	0.423	0.161	38.04	0*	<0.01
			ka	+	-	0.497	0.149	30.00	60*	<0.05
			qu	+	+	5.764	1.933	33.53	0*	<0.01
			is	+	+	0.613	0.141	23.04	0*	<0.001
			pe	+	+	0.249	0.082	32.72	all	>0.05
			cy	+	+	58.624	14.715	25.10	0/10*	<0.05
			de	+	+	0.398	0.090	22.55	10/20*	<0.05
			c	+	+	1.676	1.649	98.37	10*	<0.01
			e	+	+	0.297	0.288	97.05	0/10*	<0.05

Table S10. Data for Fig. 2 and Figure S6

Figure	Hydrolysis [min]	Sample type	Substance	LOD passed	LOQ passed	Normalized response	STDEV	RSD [%]	Compared to	*P*-value
2, S6	60	non-extracted hydrolysed	ta	+	+	0.404	0.089	21.96	0/10*	<0.05
			ka	+	+	0.835	0.241	28.92	20/30*	<0.05
			qu	+	+	10.675	9.176	85.96	all	>0.05
			is	+	+	0.994	0.420	42.24	0/10*	<0.05
			pe	+	+	0.206	0.057	27.84	20*	<0.05
			cy	+	+	60.902	22.446	36.86	0*	<0.01
			de	-	-	0.377	0.118	31.30	10/20*	<0.01
			c	+	-	0.128	0.091	71.34	10*	<0.01
			e	-	-	n.d	n.d	n.d	n.d.	n.d.
2, S6	120	non-extracted hydrolysed	ta	+	+	0.371	0.076	20.45	0/10*	<0.05
			ka	+	+	1.68	2.491	148.10	all	>0.05
			qu	+	+	8.000	2.209	27.61	0/20*	<0.05
			is	+	+	1.196	1.159	96.97	all	>0.05
			pe	+	+	0.236	0.029	12.30	20*	<0.05
			cy	+	+	68.922	7.667	11.12	0/10*	<0.001
			de	-	-	0.439	0.117	26.72	10*	<0.01
			c	-	-	n.d	n.d	n.d	n.d.	n.d.
			e	-	-	n.d	n.d	n.d	n.d.	n.d.

For experimental procedures see methods, text, Fig. 2 and Fig. S6. "n.d." = not determined., "ta" = taxifolin, "ka" = kaempferol, "qu" = quercetin, "is" = isorhamnetin, "pe" = pelargonidin, "cy" = cyaniding, "de" = delphinidin, "c" = catechin, "e" = epicatechin, "pB2" = procyanidin B2. For LOD/LOQ see methods. Responses below LOQ were only used for plotting in Fig. S6 but not for drawing the decision. Note that the hydrolysis time for extracted non-hydrolysed samples only refers to the sample which was subsequently used for the respective extracted hydrolysed and non-extracted hydrolysed sample. These extracted non-hydrolysed samples were not hydrolysed. "*" = for all other seed numbers: $P>0.05$. Please note, this excludes seed numbers for which LOD is not passed. For all, significance was determined using the Welch test.
