# Supplementary material for: A fast and simple LC-MS-based characterization of the flavonoid biosynthesis pathway for few seed(ling)s
Source: BMC Plant Biol. 2016 Sep 1;16(1):190. doi: 10.1186/s12870-016-0880-7 (PMC5007998; doi:10.1186/s12870-016-0880-7)
Supplement: Additional file 5: Figure S7. — Effect of hydrolysis on reference substances. Effect of hydrolysis on analysed flavonoids; and Figure S8. Release of pelargonidin from the internal standard and cyanidin from pelargonidin. (PDF 493 kb) [file 12870_2016_880_MOESM5_ESM.pdf]

# Additional_file_5

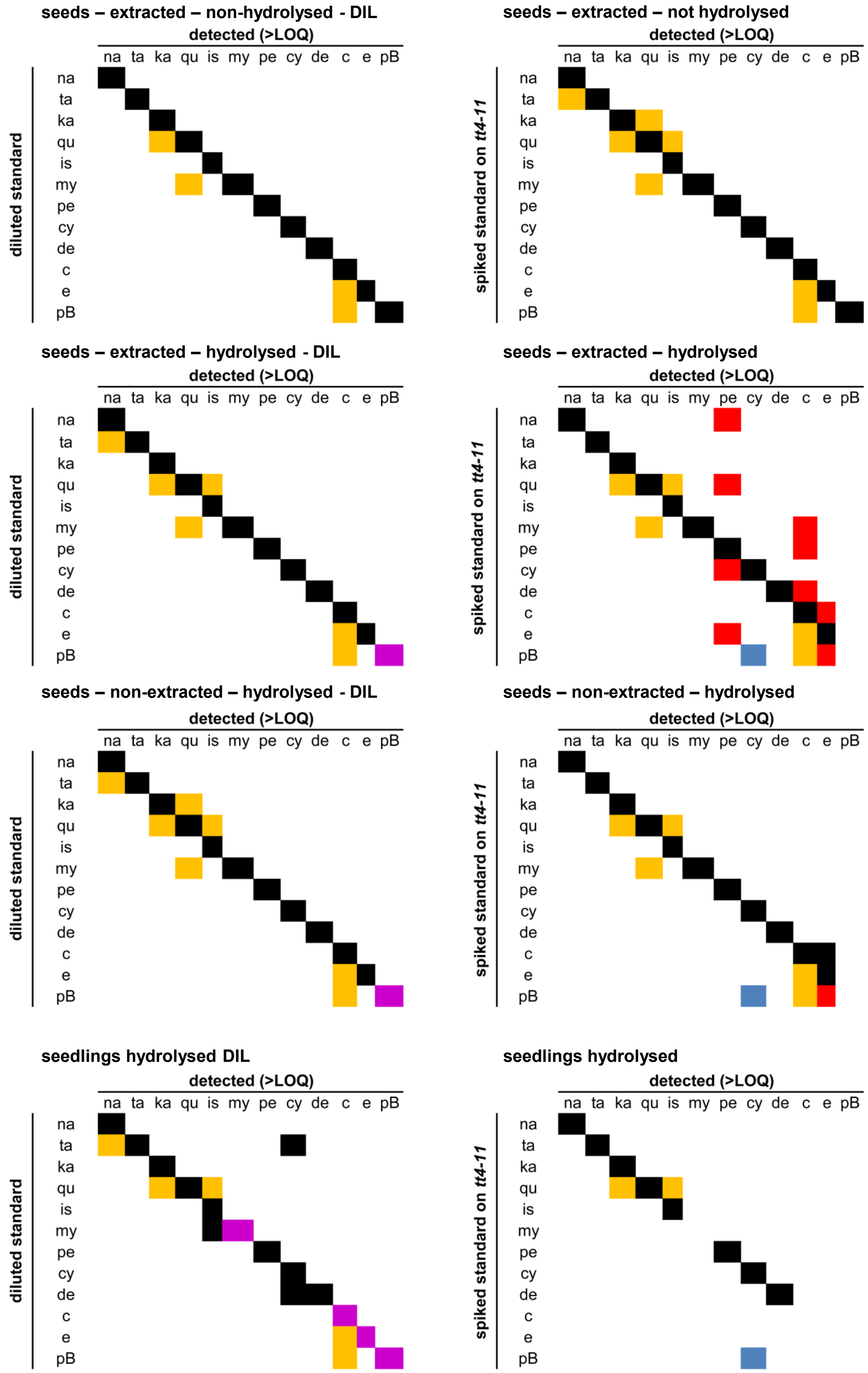

**Figure S7: Effect of hydrolysis on analyzed flavonoids.**
Diagrams show the effect of hydrolysis for each individual analyzed reference substance from our set of flavonoids using the LC-ESI-MS(MRM) setting. Labels on the left indicate the substance tested. A box is colored, when the above mentioned substance was detected in quantifiable amount. Left column of plots: dilution of the tested standard with the same factor introduced through the protocol but without any treatment. Right column of plots: Each standard mixed with the respective internal standard was spiked on 10 tt4-11 seeds. Given is the result after the indicated extraction and treatment procedure. For further details see Additional_file_9.pdf: Methods S2. Each row of two plots represent the different fraction for seed and seedlings. From top to bottom: extracted non-hydrolysed, extracted hydrolysed, non-extracted hydrolysed samples from seeds and extracted hydrolysed samples from seedlings, respectively. **Purple:** these substances were not stable through hydrolyis. **Red:** In case of absence of a substance in the dilution, these substances were detected in quantifiable amounts upon the treatment. Only few substances from our set were converted or released upon hydrolysis from another substance. Note that catechin and epicatechin can epimerize during hydrolysis (see Fig. 3). **Blue:** The published release of cyanidin is supported. **Orange:** The dilution factors for both types of soluble samples are equal. These substances were most likely in the stock solution just below or slightly above the threshold for LOQ when diluted.

na: naringenin, ta: taxifolin, ka: kaempferol, qu: quercetin, is: isorhamnetin, my: myricetin, pe: pelargonidin, cy: cyanidin, de: delphinidin, c: catechin, e: epicatechin, pB: procyanidinB2.

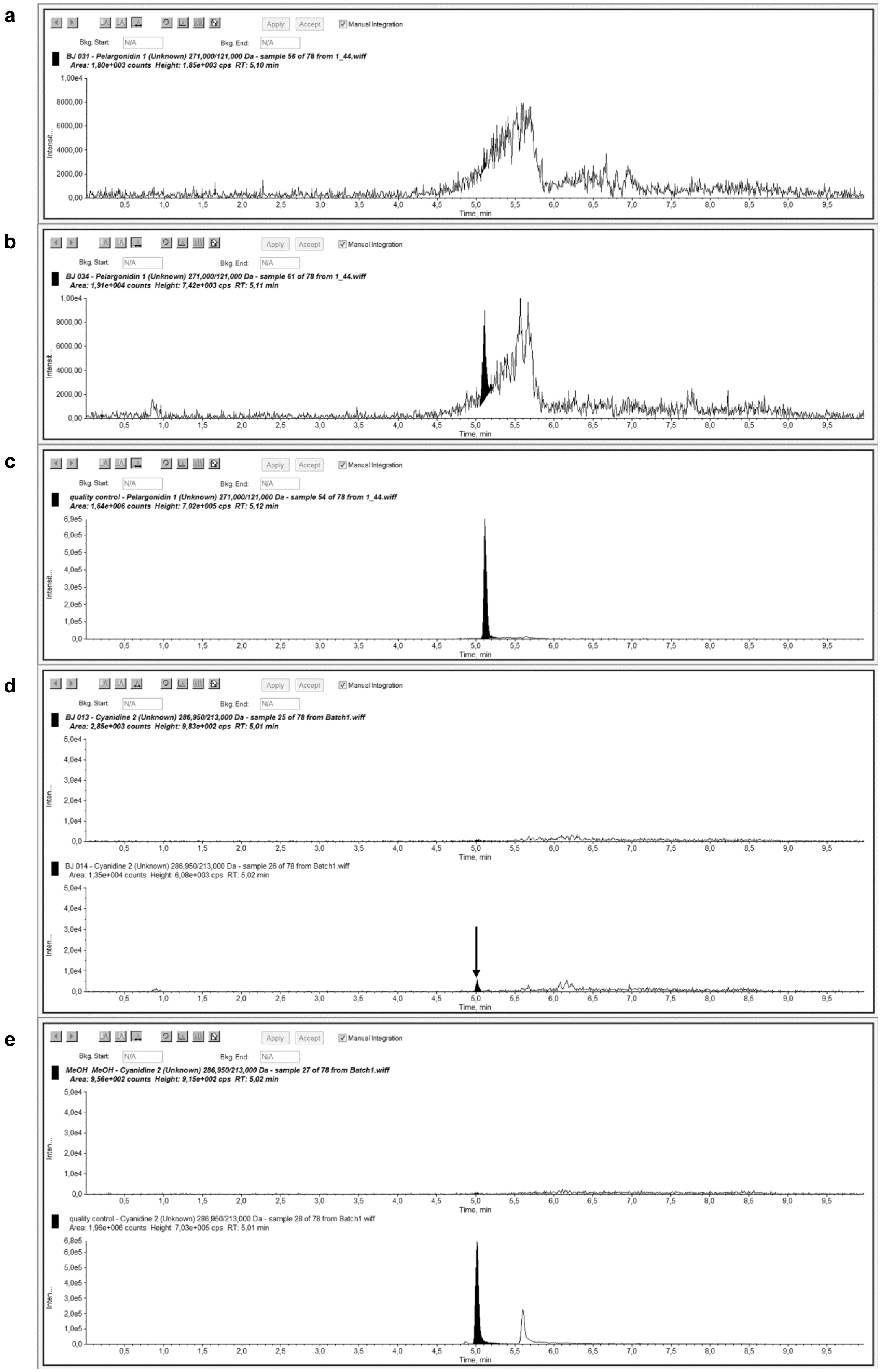
a
BJ 031 - Pelargonidin 1 (Unknown) 271,000/121,000 Da - sample 56 of 78 from 1_44.wiff
Area: 1,80e+003 counts Height: 1,85e+003 cps RT: 5,10 min
Time, min
b
BJ 034 - Pelargonidin 1 (Unknown) 271,000/121,000 Da - sample 61 of 78 from 1_44.wiff
Area: 1,91e+004 counts Height: 7,42e+003 cps RT: 5,11 min
c
quality control - Pelargonidin 1 (Unknown) 271,000/121,000 Da - sample 54 of 78 from 1_44.wiff
Area: 1,64e+006 counts Height: 7,02e+005 cps RT: 5,12 min
d
BJ 013 - Cyanidine 2 (Unknown) 286,950/213,000 Da - sample 25 of 78 from Batch1.wiff
Area: 2,85e+003 counts Height: 9,83e+002 cps RT: 5,01 min
BJ 014 - Cyanidine 2 (Unknown) 286,950/213,000 Da - sample 26 of 78 from Batch1.wiff
Area: 1,35e+004 counts Height: 6,08e+003 cps RT: 5,02 min
e
MeOH MeOH - Cyanidine 2 (Unknown) 286,950/213,000 Da - sample 27 of 78 from Batch1.wiff
Area: 9,56e+002 counts Height: 9,15e+002 cps RT: 5,02 min
quality control - Cyanidine 2 (Unknown) 286,950/213,000 Da - sample 28 of 78 from Batch1.wiff
Area: 1,96e+006 counts Height: 7,03e+005 cps RT: 5,01 min
Apply
Accept
Manual Integration
Bkg. Start
Bkg. End:
N/A
Intensit...
Inten...
Time, min

**Figure S8: Release of pelargonidin from the internal standard and cyanidin from pelargonidin.**

LC-ESI-MS(MRM) derived representative EICs from the spiking experiment documented in Figure S7. **a,b** EICs of perlagonidin from the internal standard before and after hydrolysis respectively. In these samples only the internal standards were spiked. Note the appearance of perlargonidin after hydrolysis. **c** EIC of perlagonidin from the quality control, **d,e** EICs for cyanidin. d: Upper: diluted pelargonidin standard. Lower: spiked and treated pelargonidin standard (protocol for extracted hydrolysed samples). e: Upper: MeOH blank. Lower: cyanidin from the quality control. Note that these samples were analysed following each other.
