# Supplementary material for: A fast and simple LC-MS-based characterization of the flavonoid biosynthesis pathway for few seed(ling)s
Source: BMC Plant Biol. 2016 Sep 1;16(1):190. doi: 10.1186/s12870-016-0880-7 (PMC5007998; doi:10.1186/s12870-016-0880-7)
Supplement: Additional file 6: Figure S9. — Minimizing the number of seeds. Reduction of seed material down to one seed – repetition; and Table S11. Data for Fig. 4a,b. (PDF 356 kb) [file 12870_2016_880_MOESM6_ESM.pdf]

Additional_file_6

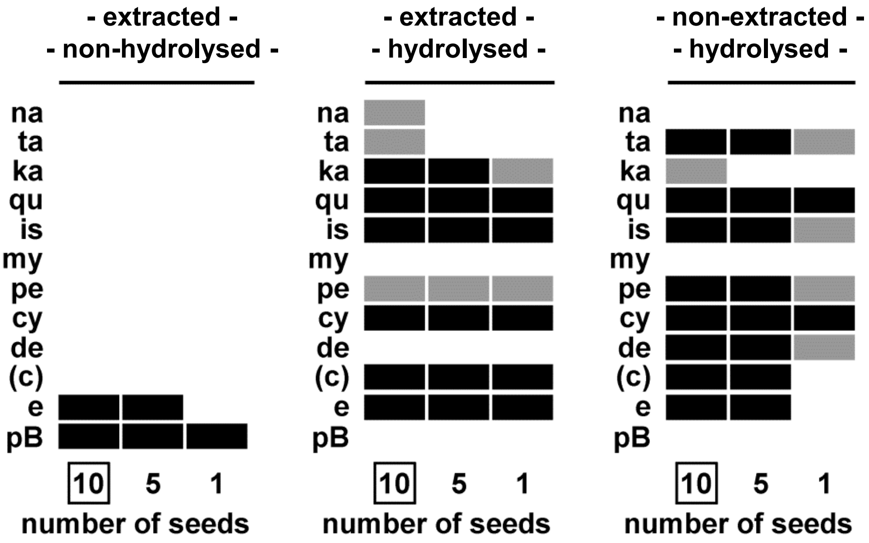

Figure S9: Reduction of seed material down to one seed – repetition. Flavonoids detected in the three seed samples, extracted non-hydrolysed, extracted hydrolysed, and non-extracted hydrolysed. Filled boxes represent that more than half of the replicates passed LOQ (black) or LOD (grey), respectively. Square box represent the seed number chosen.

ta: taxifolin, ka: kaempferol, qu: quercetin, is: isorhamnetin, my: myricetin, pe: pelargonidin, cy: cyanidin, de: delphinidin, c: catechin, e: epicatechin, pB: procyanidinB2.

Table S11. Data for Figure 4a,b

Figure	No. of seeds	Sample type	Substance	LOD passed	LOQ passed	Normalized response	STDEV	RSD [%]	Compared to	*P*-value	Linearity (r^2)	
4a,b	50	extracted non-hydrolysed	ta	+	+	0.017	0.004	22.67	n.d.	n.d.		
			e	+	+	0.077	0.028	35.85	5/10*	<0.01		
			pB	+	+	0.062	0.030	48.57	5/10*	<0.05	substance	r^2
4a,b	25	extracted non-hydrolysed	ta	+	-	n.d.	n.d.	n.d.	n.d.	n.d		
			e	+	+	0.059	0.033	56.97	5*	<0.05		
			pB	+	+	0.059	0.014	24.64	5/10*	<0.01		
4a,b	10	extracted non-hydrolysed	ta	-	-	n.d.	n.d.	n.d.	n.d.	n.d		
			e	+	+	0.020	0.005	25.65	50*	<0.01	e	0.89
			pB	+	+	0.022	0.011	49.74	25/50*	<0.05		
4a,b	5	extracted non-hydrolysed	ta	-	-	n.d.	n.d.	n.d.	n.d.	n.d		
			e	+	+	0.013	0.008	63.55	25/50*	<0.05		
			pB	+	+	0.012	0.011	91.24	25/50*	<0.05		
4a,b	1	extracted non-hydrolysed	ta	-	-	n.d.	n.d.	n.d.			pB	0.69
			e	+	-	n.d.	n.d.	n.d.	n.d.	n.d.		
			pB	+	-	n.d.	n.d.	n.d.				
4a,b	50	extracted hydrolysed	ta	+	+	0.049	0.008	16.73	n.d.	n.d.		
			ka	+	+	4.043	0.342	8.46	1/5/10*	<0.001		
			qu	+	+	20.119	1.316	6.54	1/5/10/25/50	<0.001	sub stance	r^2
			is	+	+	5.348	0.592	11.06	1/5/10/25	<0.01		
			pe	+	+	0.068	0.006	8.45	n.d.	n.d.		
			cy	+	+	1.820	0.373	20.48	1/5/10*	<0.001	ta	n.d.
			c	+	+	2.555	0.458	17.94	1/5/10/25	<0.05		
			e	+	+	0.602	0.093	15.36	1/5/10/25	<0.05	ka	0.87
4a,b	25	extracted hydrolysed	ta	+	-	n.d.	n.d.	n.d.	n.d.	n.d.		
			ka	+	+	3.343	1.115	33.35	1/5/10*	<0.01	qu	0.97
			qu	+	+	13.086	0.950	7.26	1/5/10/25	<0.001		
			is	+	+	3.493	0.797	22.82	1/5/10/50	<0.01	is	0.96
			pe	+	-	n.d.	n.d.	n.d.	n.d.	n.d.		
			cy	+	+	1.372	0.236	17.21	1/5/10*	<0.001	pe	n.d.
			c	+	+	1.771	0.355	20.04	1/5/10/50			
			e	+	+	0.432	0.099	23.01	1/5/10/50	<0.05	cy	0.93
4a,b	10	extracted hydrolysed	ta	+	-	n.d.	n.d.	n.d.	n.d.	n.d.		
			ka	+	+	1.094	0.787	71.96	1/25/50*	<0.05	c	0.96
			qu	+	+	5.197	1.008	19.40	1/5/25/50	<0.01		
			is	+	+	1.115	0.484	43.43	1/5/25/50	<0.05	e	0.94

Table S11. Data for Figure 4a,b

Figure	No. of seeds	Sample type	Substance	LOD passed	LOQ passed	Normalized response	STDEV	RSD [%]	Compared to	*P*-value	Linearity (r²)
4a,b	10	extracted hydrolysed	pe	+	-	n.d	n.d.	n.d.	n.d.	n.d.	see above
			cy	+	+	0.617	0.177	28.66	1/5/25/50	<0.05	
			c	+	+	0.784	0.220	28.06	1/5/25/50	<0.05	
			e	+	+	0.180	0.041	22.57	1/5/25/50	<0.01	
4a,b	5	extracted hydrolysed	ta	-	-	n.d	n.d	n.d	n.d.	n.d.	
			ka	+	+	0.383	0.064	16.78	1/25/50*	<0.01	
			qu	+	+	2.561	0.351	13.70	1/10/25/50	<0.01	
			is	+	+	0.461	0.073	15.74	1/10/25/50	<0.05	
			pe	+	-	n.d	n.d.	n.d.	n.d.	n.d.	
			cy	+	+	0.334	0.142	42.64	10/25/50*	<0.05	
			c	+	+	0.429	0.201	46.80	1/10/25/50	<0.05	
			e	+	+	0.090	0.031	34.88	1/10/25/50	<0.05	
4a,b	1	extracted hydrolysed	ta	-	-	n.d	n.d	n.d	n.d.	n.d.	
			ka	+	+	0.089	0.038	43.26	5/10/25/50	<0.05	
			qu	+	+	0.588	0.262	44.63	5/10/25/50	<0.001	
			is	+	+	0.119	0.037	31.11	5/10/25/50	<0.01	
			pe	+	-	n.d.	n.d.	n.d.	n.d.	n.d.	
			cy	+	+	0.385	0.080	20.80	10/25/50*	<0.05	
			c	+	+	0.157	0.076	48.72	5/10/25/50	<0.05	
			e	+	+	0.037	0.018	50.40	5/10/25/50	<0.05	
4a,b	50	non-extracted hydrolysed	ta	+	+	1.010	0.051	5.06	10/25	<0.001	substance / r²
			ka	+	+	1.826	0.578	31.64	10/25	<0.05	
			qu	+	+	22.180	4.616	20.81	1/5/10/25	<0.01	
			is	+	+	2.906	0.790	27.18	1/5/10/25	<0.01	
			pe	+	+	0.723	0.088	12.18	10/25	<0.001	
			cy	+	+	84.660	3.849	4.55	10/25	<0.001	ta 1.00
			de	+	+	2.560	0.212	8.28	10/25	<0.001	ka 0.98
			c	+	+	29.120	4.705	16.16	5/10/25	<0.001	qu 0.95
			e	+	+	7.512	1.378	18.35	10/25	<0.001	is 0.75
4a,b	25	non-extracted hydrolysed	ta	+	+	0.548	0.046	8.44	10/50	<0.001	pe 0.97
			ka	+	+	0.772	0.339	43.95	10/50	<0.05	cy 1.00
			qu	+	+	7.016	0.842	12.00	1/5/10/50	<0.01	de 0.97
			is	+	+	1.060	0.306	28.82	5/10/50	<0.05	c 1.00
			pe	+	+	0.336	0.039	11.72	10/50	<0.001	e 1.00
			cy	+	+	52.940	4.312	8.15	10/50	<0.001	

Table S11. Data for Figure 4a,b

Figure	No. of seeds	Sample type	Substance	LOD passed	LOQ passed	Normalized response	STDEV	RSD [%]	Compared to	*P*-value	Linearity (r²)
4a,b	25	non-extracted hydrolysed	de	+	+	0.971	0.222	22.82	10/50	<0.01	see above
			c	+	+	11.906	2.932	24.63	5/10/50	<0.001	
			e	+	+	3.086	0.950	30.79	10/50	<0.01	
4a,b	10	non-extracted hydrolysed	ta	+	+	0.255	0.030	11.90	25/50	<0.001	
			ka	+	+	0.347	0.113	32.47	25/50	<0.05	
			qu	+	+	3.656	1.268	34.68	1/5/25/50	<0.05	
			is	+	+	0.524	0.035	6.63	5/25/50*	<0.05	
			pe	+	+	0.194	0.029	15.16	25/50	<0.001	
			cy	+	+	35.760	3.009	8.41	25/50	<0.001	
			de	+	+	0.406	0.035	8.68	25/50	<0.01	
			c	+	+	3.100	2.932	94.60	5/25/50	<0.05	
			e	+	+	0.657	0.950	144.59	25/50	<0.01	
4a,b	5	non-extracted hydrolysed	ta	+	-	n.d	n.d	n.d	n.d	n.d	
			ka	+	-	n.d	n.d	n.d	n.d	n.d	
			qu	+	+	1.212	0.260	21.47	1/10/25/50	<0.05	
			is	+	+	0.323	0.102	31.43	10/25/50*	<0.01	
			pe	+	-	n.d	n.d	n.d	n.d	n.d	
			cy	+	-	n.d	n.d	n.d	n.d	n.d	
			de	+	-	n.d	n.d	n.d	n.d	n.d	
			c	+	+	0.166	0.126	76.31	10/25/50	<0.05	
			e	+	-	n.d	n.d	n.d	n.d	n.d	
4a,b	1	non-extracted hydrolysed	ta	+	-	n.d	n.d	n.d	n.d	n.d	
			ka	+	-	n.d	n.d	n.d	n.d	n.d	
			qu	+	+	0.288	0.179	62.08	5/10/25/50	<0.01	
			is	+	+	1.022	0.840	82.13	50*	<0.01	
			pe	+	-	n.d	n.d	n.d	n.d	n.d	
			cy	+	-	n.d	n.d	n.d	n.d	n.d	
			de	+	-	n.d	n.d	n.d	n.d	n.d	
			c	+	-	n.d	n.d	n.d	n.d	n.d	
			e	+	-	n.d	n.d	n.d	n.d	n.d	

For experimental procedures see methods, text and Fig. 4. Note that peak areas and statistics are only determined when LOQ was passed. Only substances are shown for which at least for one seed number LOQ was passed for a given sample type.

“n.d.” = not determined. “na” = naringenin, “ta” = taxifolin, “ka” = kaempferol, “qu” = quercetin, “is” = isorhamnetin, “my” = myricetin, “pe” = pelargonidin, “cy” = cyaniding, “de” = delphinidin, “c” = catechin, “e” = epicatechin, “pB” = procyanidin B2. For LOD/LOQ see methods. “Normalized response” = normalized to the respective internal standard. “*” = for all other seed numbers: P>0.05. Please note, this excludes seed numbers for which LOQ is not passed. For all, significance was determined using the Welch test.
