# Supplementary material for: A fast and simple LC-MS-based characterization of the flavonoid biosynthesis pathway for few seed(ling)s
Source: BMC Plant Biol. 2016 Sep 1;16(1):190. doi: 10.1186/s12870-016-0880-7 (PMC5007998; doi:10.1186/s12870-016-0880-7)
Supplement: Additional file 7: Figure S10. — Proof of principle: revealing shifts in the pathway. Application (seeds of mutants) – normalized responses; Table S12. Data for Fig. 4c-e.; and Table S13. Data for Fig. 4c,d – significance. (PDF 731 kb) [file 12870_2016_880_MOESM7_ESM.pdf]

Additional_file_7

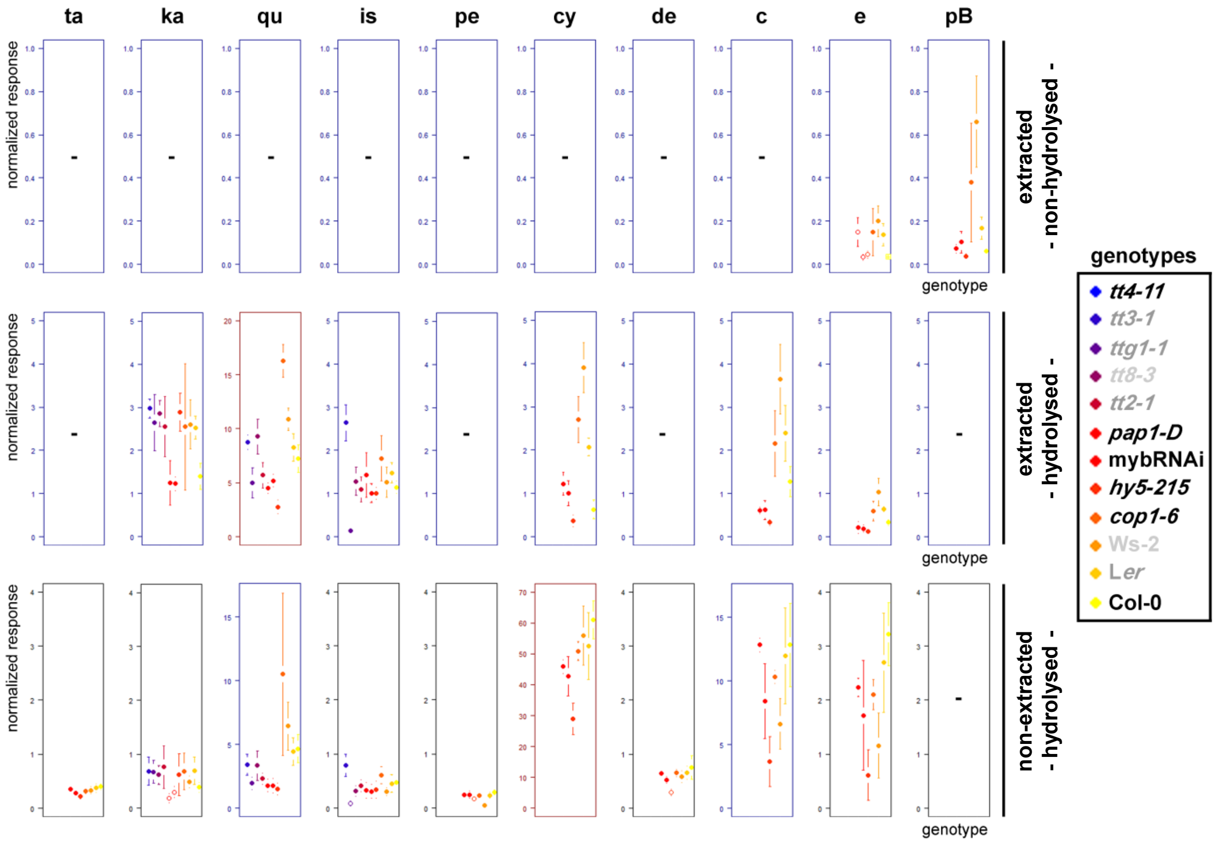

Figure S10: Application (seeds of mutants) – normalized responses. Plots showing the normalized response (normalized with the respective internal standard) of the different flavonoids tested in the different mutants. The three rows represent the 3 fraction extracted non-hydrolysed, extracted hydrolysed, non-extracted hydrolysed from top to bottom respectively. The different color of the outer rim of each plot correspond to the different scales used, the different colors of the dots represent the different mutants tested. -: In none of the lines, a quantifiable amount of this substance was detected in this samples type. Filled dots: LOQ is passed. Empty dots: LOD is passed, LOQ is not passed. Error bars = STDEV.

ta: taxifolin, ka: kaempferol, qu: quercetin, is: isorhamnetin, pe: pelargonidin, cy: cyanidin, de: delphinidin, c: catechin, e: epicatechin, pB: procyanidinB2.

Table S12. Data for Figure 4c-e

						Normalized to internal standard				Normalized to kaempferol			
**Figure**	**Name**	**Sample type**	**Substance**	**LOD passed**	**LOQ passed**	**Normalized response**	**STDEV**	**RSD [%]**	**Fold difference to wt**	**Normalized response**	**STDEV**	**RSD [%]**	**Fold difference to wt**
4c,e	*tt4-11* (Col-0)	extracted non-hydrolysed	e	-	-	n.d.	n.d.	n.d.	0	n.d.	n.d.	n.d.	n.d.
			pB	-	-	n.d.	n.d.	n.d.	0	n.d.	n.d.	n.d.	n.d.
4d	*tt7-1* (L*er*)	extracted non-hydrolysed	e	-	-	n.d.	n.d.	n.d.	0	n.d.	n.d.	n.d.	n.d.
			pB	-	-	n.d.	n.d.	n.d.	0	n.d.	n.d.	n.d.	n.d.
4c,e	*tt3-1* (L*er*)	extracted non-hydrolysed	e	-	-	n.d.	n.d.	n.d.	0	n.d.	n.d.	n.d.	n.d.
			pB	-	-	n.d.	n.d.	n.d.	0	n.d.	n.d.	n.d.	n.d.
4c,e	*ttg1-1* (L*er*)	extracted non-hydrolysed	e	-	-	n.d.	n.d.	n.d.	0	n.d.	n.d.	n.d.	n.d.
			pB	-	-	n.d.	n.d.	n.d.	0	n.d.	n.d.	n.d.	n.d.
4c,e	*tt8-3* (Ws-2)	extracted non-hydrolysed	e	-	-	n.d.	n.d.	n.d.	0	n.d.	n.d.	n.d.	n.d.
			pB	-	-	n.d.	n.d.	n.d.	0	n.d.	n.d.	n.d.	n.d.
4c,e	*ttg2-1* (L*er*)	extracted non-hydrolysed	e	-	-	n.d.	n.d.	n.d.	0	n.d.	n.d.	n.d.	n.d.
			pB	-	-	n.d.	n.d.	n.d.	0	n.d.	n.d.	n.d.	n.d.
4c,e	*pap1-D* (Col-0)	extracted non-hydrolysed	e	+	-	0.150	0.067	44.67	4.2	n.d.	n.d.	n.d.	n.d.
			pB	+	+	0.073	0.020	27.40	1.2	n.d.	n.d.	n.d.	n.d.
4c,e	*myb*RNAi (Col-0)	extracted non-hydrolysed	e	+	-	0.033	0.014	42.42	0.9	n.d.	n.d.	n.d.	n.d.
			pB	+	+	0.104	0.050	48.08	1.7	n.d.	n.d.	n.d.	n.d.
4c,e	*hy5-215* (Col-0)	extracted non-hydrolysed	e	+	-	0.046	0.017	36.96	1.3	n.d.	n.d.	n.d.	n.d.
			pB	+	+	0.039	0.011	28.21	0.6	n.d.	n.d.	n.d.	n.d.

Table S12. Data for Figure 4c-e

						Normalized to internal standard				Normalized to kaempferol			
**Figure**	**Name**	**Sample type**	**Substance**	**LOD passed**	**LOQ passed**	**Normalized response**	**STDEV**	**RSD [%]**	**Fold difference to wt**	**Normalized response**	**STDEV**	**RSD [%]**	**Fold difference to wt**
4c,e	*cop1-6* (Col-0)	extracted non-hydrolysed	e	+	+	0.150	0.109	72.67	4.2	n.d.	n.d.	n.d.	n.d.
			pB	+	+	0.380	0.275	72.37	6.2	n.d.	n.d.	n.d.	n.d.
4c,e	Col-0	extracted non-hydrolysed	e	+	-	0.036	0.012	33.33	1	n.d.	n.d.	n.d.	n.d.
			pB	+	+	0.061	0.010	16.39	1	n.d.	n.d.	n.d.	n.d.
4c-e	L*er*	extracted non-hydrolysed	e	+	+	0.137	0.052	37.96	1	n.d.	n.d.	n.d.	n.d.
			pB	+	+	0.169	0.052	30.77	1	n.d.	n.d.	n.d.	n.d.
4c,e	Ws-2	extracted non-hydrolysed	e	+	+	0.200	0.071	35.50	1	n.d.	n.d.	n.d.	n.d.
			pB	+	+	0.660	0.210	31.82	1	n.d.	n.d.	n.d.	n.d.
4c,e	*tt4-11* (Col-0)	extracted hydrolysed	ka	-	-	n.d.	n.d.	n.d.	0	n.d.	n.d.	n.d.	0
			qu	-	-	n.d.	n.d.	n.d.	0	n.d.	n.d.	n.d.	0
			is	-	-	n.d.	n.d.	n.d.	0	n.d.	n.d.	n.d.	0
			pe	-	-	n.d.	n.d.	n.d.	0	n.d.	n.d.	n.d.	0
			cy	-	-	n.d.	n.d.	n.d.	0	n.d.	n.d.	n.d.	0
			c	-	-	n.d.	n.d.	n.d.	0	n.d.	n.d.	n.d.	0
			e	-	-	n.d.	n.d.	n.d.	0	n.d.	n.d.	n.d.	0
4d	*tt7-1* (L*er*)	extracted hydrolysed	ka	+	+	38.628	15.215	39.39	15.4	1.000	0.000	0.00	1.0
			qu	-	-	n.d.	n.d.	n.d.	0	n.d.	n.d.	n.d.	0
			is	-	-	n.d.	n.d.	n.d.	0	n.d.	n.d.	n.d.	0
			pe	+	+	1.374	0.435	31.65	31.2	0.041	0.022	53.69	2.4
			cy	-	-	n.d.	n.d.	n.d.	0	n.d.	n.d.	n.d.	0
			c	-	-	n.d.	n.d.	n.d.	0	n.d.	n.d.	n.d.	0
			e	-	-	n.d.	n.d.	n.d.	0	n.d.	n.d.	n.d.	0
4c,e	*tt3-1* (L*er*)	extracted hydrolysed	ka	+	+	2.973	0.224	7.53	1.2	1.000	0.000	0.00	1.0
			qu	+	+	8.743	0.651	7.45	1.1	2.953	0.296	10.02	0.9
			is	+	+	2.637	0.421	15.97	1.8	0.884	0.102	11.54	1.5
			pe	-	-	n.d.	n.d.	n.d.	0	n.d.	n.d.	n.d.	n.d.

Table S12. Data for Figure 4c-e

						Normalized to internal standard				Normalized to kaempferol			
**Figure**	**Name**	**Sample type**	**Substance**	**LOD passed**	**LOQ passed**	**Normalized response**	**STDEV**	**RSD [%]**	**Fold difference to wt**	**Normalized response**	**STDEV**	**RSD [%]**	**Fold difference to wt**
4c,e	*tt3-1* (L*er*)	extracted hydrolysed	cy	-	-	n.d.	n.d.	n.d.	0	n.d.	n.d.	n.d.	0
			c	-	-	n.d.	n.d.	n.d.	0	n.d.	n.d.	n.d.	0
			e	-	-	n.d.	n.d.	n.d.	0	n.d.	n.d.	n.d.	0
4c,e	*ttg1-1* (L*er*)	extracted hydrolysed	ka	+	+	2.641	0.651	24.65	1.0	1.000	0.000	0.00	1.0
			qu	+	+	4.992	1.416	28.37	0.6	1.895	0.226	11.93	0.6
			is	+	+	0.148	0.037	25.00	0.1	0.057	0.008	14.04	0.1
			pe	-	-	n.d.	n.d.	n.d.	0	n.d.	n.d.	n.d.	0
			cy	-	-	n.d.	n.d.	n.d.	0	n.d.	n.d.	n.d.	0
			c	-	-	n.d.	n.d.	n.d.	0	n.d.	n.d.	n.d.	0
			e	-	-	n.d.	n.d.	n.d.	0	n.d.	n.d.	n.d.	0
4c,e	*tt8-3* (Ws-2)	extracted hydrolysed	ka	+	+	2.854	0.306	10.72	1.1	1.000	0.000	0.00	1.0
			qu	+	+	9.285	1.612	17.36	0.9	3.270	0.417	12.75	0.8
			is	+	+	1.285	0.325	25.29	1.0	0.450	0.093	20.67	0.9
			pe	+	-	n.d.	n.d.	n.d.	0	n.d.	n.d.	n.d.	0
			cy	-	-	n.d.	n.d.	n.d.	0	n.d.	n.d.	n.d.	0
			c	-	-	n.d.	n.d.	n.d.	0	n.d.	n.d.	n.d.	0
			e	-	-	n.d.	n.d.	n.d.	0	n.d.	n.d.	n.d.	0
4c,e	*ttg2-1* (L*er*)	extracted hydrolysed	ka	+	+	2.554	0.704	27.56	1.0	1.000	0.000	0.00	0.3
			qu	+	+	5.704	1.173	20.56	0.7	2.297	0.542	23.60	0.7
			is	+	+	1.098	0.291	26.50	0.7	0.440	0.099	22.50	0.1
			pe	-	-	n.d.	n.d.	n.d.	0	n.d.	n.d.	n.d.	0
			cy	-	-	n.d.	n.d.	n.d.	0	n.d.	n.d.	n.d.	0
			c	-	-	n.d.	n.d.	n.d.	0	n.d.	n.d.	n.d.	0
			e	-	-	n.d.	n.d.	n.d.	0	n.d.	n.d.	n.d.	0
4c,e	*pap1-D (Col-0)*	extracted hydrolysed	ka	+	+	1.253	0.514	41.02	0.9	1.000	0.000	0.00	1.0
			qu	+	+	4.509	0.492	10.91	0.6	3.953	2.782	70.38	0.7
			is	+	+	1.431	0.516	36.06	1.2	1.177	0.152	12.91	1.4
			pe	+	-	n.d.	n.d.	n.d.	0	n.d.	n.d.	n.d.	0
			cy	+	+	1.221	0.268	21.95	1.9	1.049	0.566	53.96	2.3
			c	+	+	0.609	0.076	12.48	0.5	0.531	0.619	116.57	0.6
			e	+	+	0.214	0.138	64.49	0.6	0.160	0.135	84.38	0.6

Table S12. Data for Figure 4c-e

						Normalized to internal standard				Normalized to kaempferol			
**Figure**	**Name**	**Sample type**	**Substance**	**LOD passed**	**LOQ passed**	**Normalized response**	**STDEV**	**RSD [%]**	**Fold difference to wt**	**Normalized response**	**STDEV**	**RSD [%]**	**Fold difference to wt**
4c,e	*myb*RNAi (Col-0)	extracted hydrolysed	ka	+	+	1.230	0.160	13.01	0.9	1.000	0.000	0.00	1.0
			qu	+	+	5.202	0.552	10.61	0.7	4.303	0.253	5.88	0.8
			is	+	+	1.011	0.220	21.76	0.9	0.829	0.024	2.90	1.0
			pe	+	-	n.d.	n.d.	n.d.	0	n.d.	n.d.	n.d.	0
			cy	+	+	1.004	0.294	29.28	1.6	0.838	0.037	4.42	1.8
			c	+	+	0.619	0.225	36.35	0.5	0.498	0.026	5.22	0.5
			e	+	+	0.191	0.086	45.03	0.6	0.153	0.015	9.80	0.6
4c,e	*hy5-215* (Col-0)	extracted hydrolysed	ka	+	+	2.887	0.434	15.03	2.1	1.000	0.000	0.00	1.0
			qu	+	+	2.751	0.637	23.16	0.4	0.970	0.862	88.87	0.2
			is	+	+	1.012	0.135	13.34	0.9	0.352	0.204	57.95	0.4
			pe	+	-	n.d.	n.d.	n.d.	0	n.d.	n.d.	n.d.	0
			cy	+	+	0.371	0.133	35.85	0.6	0.127	0.316	248.82	0.3
			c	+	+	0.332	0.067	20.18	0.3	0.117	0.135	115.38	0.1
			e	+	+	0.126	0.037	29.37	0.4	0.045	0.056	124.44	0.2
4c,e	*cop1-6* (Col-0)	extracted hydrolysed	ka	+	+	2.549	1.467	57.55	1.8	1.000	0.000	0.00	1.0
			qu	+	+	16.272	1.506	9.26	2.3	7.590	1.125	14.82	1.4
			is	+	+	1.817	0.517	28.45	1.6	0.779	0.294	37.74	0.9
			pe	+	-	n.d.	n.d.	n.d.	0	n.d.	n.d.	n.d.	0
			cy	+	+	2.708	0.534	19.72	4.3	1.270	0.281	22.13	2.8
			c	+	+	2.156	0.755	35.02	1.7	1.023	0.163	15.93	1.1
			e	+	+	0.601	0.226	37.60	1.8	0.267	0.043	16.10	1.1
4c,e	Col-0	extracted hydrolysed	ka	+	+	1.400	0.297	21.21	1	1.000	0	0.00	1
			qu	+	+	7.227	1.264	17.49	1	5.387	1.480	27.47	1
			is	+	+	1.146	0.145	12.65	1	0.839	0.152	18.12	1
			pe	+	-	n.d.	n.d.	n.d.	1	n.d.	n.d.	n.d.	1
			cy	+	+	0.631	0.219	34.71	1	0.461	0.178	38.61	1
			c	+	+	1.283	0.348	27.12	1	0.916	0.130	14.19	1
			e	+	+	0.332	0.062	18.67	1	0.248	0.088	35.48	1
4c-e	L*er*	extracted hydrolysed	ka	+	+	2.529	0.267	10.56	1	1.000	0.000	0.00	1
			qu	+	+	8.269	1.250	15.12	1	3.279	0.416	12.69	1
			is	+	+	1.483	0.239	16.12	1	0.587	0.070	11.93	1
			pe	+	-	0.044	0.011	25.00	1	0.017	0.004	23.53	1
			cy	+	+	2.073	0.205	9.89	1	0.825	0.097	11.76	1

Table S12. Data for Figure 4c-e

Figure	Name	Sample type	Substance	LOD passed	LOQ passed	Normalized to internal standard: Normalized response	STDEV	RSD [%]	Fold difference to wt	Normalized to kaempferol: Normalized response	STDEV	RSD [%]	Fold difference to wt
4c-e	L*er*	extracted hydrolysed	c	+	+	2.398	0.642	26.77	1	0.954	0.246	25.79	1
			e	+	+	0.644	0.061	9.47	1	0.258	0.044	17.05	1
4c,e	Ws-2	extracted hydrolysed	ka	+	+	2.604	0.570	21.89	1	1.000	0.000	0.00	1
			qu	+	+	10.876	1.042	9.58	1	4.262	0.494	11.59	1
			is	+	+	1.268	0.351	27.68	1	0.487	0.080	16.43	1
			pe	+	-	n.d.	n.d.	n.d.	1	n.d.	n.d.	n.d.	1
			cy	+	+	3.903	0.581	14.89	1	1.534	0.307	20.01	1
			c	+	+	3.640	0.803	22.06	1	1.417	0.263	18.56	1
			e	+	+	1.038	0.319	30.73	1	0.397	0.064	16.12	1
4c,e	*tt4-11* (Col-0)	non-extracted hydrolysed	ta	-	-	n.d.	n.d.	n.d.	0	n.d.	n.d.	n.d.	0
			ka	-	-	n.d.	n.d.	n.d.	0	n.d.	n.d.	n.d.	0
			qu	-	-	n.d.	n.d.	n.d.	0	n.d.	n.d.	n.d.	0
			is	-	-	n.d.	n.d.	n.d.	0	n.d.	n.d.	n.d.	0
			pe	-	-	n.d.	n.d.	n.d.	0	n.d.	n.d.	n.d.	0
			cy	-	-	n.d.	n.d.	n.d.	0	n.d.	n.d.	n.d.	0
			de	-	-	n.d.	n.d.	n.d.	0	n.d.	n.d.	n.d.	0
			c	-	-	n.d.	n.d.	n.d.	0	n.d.	n.d.	n.d.	0
			e	-	-	n.d.	n.d.	n.d.	0	n.d.	n.d.	n.d.	0
4d	*tt7-1* (L*er*)	non-extracted hydrolysed	ta	-	-	n.d.	n.d.	n.d.	0	n.d.	n.d.	n.d.	0
			ka	+	+	12.822	3.273	25.53	18.5	1.000	0.000	0.00	1
			qu	-	-	n.d.	n.d.	n.d.	0	n.d.	n.d.	n.d.	0
			is	-	-	n.d.	n.d.	n.d.	0	n.d.	n.d.	n.d.	0
			pe	+	+	27.498	3.122	11.35	115.9	2.268	0.707	31.17	6.2
			cy	+	+*	n.d.	n.d.	n.d.	n.d.	n.d.	n.d.	n.d.	n.d.
			de	-	-	n.d.	n.d.	n.d.	0	n.d.	n.d.	n.d.	0
			c	-	-	n.d.	n.d.	n.d.	0	n.d.	n.d.	n.d.	0
			e	-	-	n.d.	n.d.	n.d.	0	n.d.	n.d.	n.d.	0
4c,e	*tt3-1* (L*er*)	non-extracted hydrolysed	ta	-	-	n.d.	n.d.	n.d.	0	n.d.	n.d.	n.d.	0
			ka	+	+	0.689	0.266	38.61	1.0	1.000	0.000	0.00	1,0
			qu	+	+	3.409	0.813	23.85	0.8	5.164	1.007	19.50	0,8
			is	+	+	0.796	0.207	26.05	1.7	1.206	0.271	22.47	1,8
			pe	-	-	n.d.	n.d.	n.d.	0	n.d.	n.d.	n.d.	0
			cy	-	-	n.d.	n.d.	n.d.	0	n.d.	n.d.	n.d.	0
			de	-	-	n.d.	n.d.	n.d.	0	n.d.	n.d.	n.d.	0

Table S12. Data for Figure 4c-e

						Normalized to internal standard				Normalized to kaempferol			
**Figure**	**Name**	**Sample type**	**Substance**	**LOD passed**	**LOQ passed**	**Normalized response**	**STDEV**	**RSD [%]**	**Fold difference to wt**	**Normalized response**	**STDEV**	**RSD [%]**	**Fold difference to wt**
4c,e	*tt3-1* (L*er*)	non-extracted hydrolysed	c	-	-	n.d.	n.d.	n.d.	0	n.d.	n.d.	n.d.	0
			e	-	-	n.d.	n.d.	n.d.	0	n.d.	n.d.	n.d.	0
4c,e	*ttg1-1* (L*er*)	non-extracted hydrolysed	ka	+	+	0.677	0.213	31.38	1.0	1.000	0.000	0.00	1,0
			qu	+	+	1.966	0.494	25.14	0.4	3.026	0.857	28.32	0,4
			is	+	-	0.084	0.049	58.61	0.2	0.124	0.065	52.70	0,2
			pe	-	-	n.d.	n.d.	n.d.	0	n.d.	n.d.	n.d.	0
			cy	-	-	n.d.	n.d.	n.d.	0	n.d.	n.d.	n.d.	0
			c	-	-	n.d.	n.d.	n.d.	0	n.d.	n.d.	n.d.	0
			e	-	-	n.d.	n.d.	n.d.	0	n.d.	n.d.	n.d.	0
4c,e	*tt8-3* (Ws-2)	non-extracted hydrolysed	ta	-	-	n.d.	n.d.	n.d.	0	n.d.	n.d.	n.d.	0
			ka	+	+	0.617	0.179	28.94	1.3	1.000	0.000	0.00	1,0
			qu	+	+	3.346	1.156	34.54	0.5	3.510	1.255	35.75	0,3
			is	+	+	0.324	0.098	30.28	1.1	0.599	0.130	21.70	0,9
			pe	-	-	n.d.	n.d.	n.d.	0	n.d.	n.d.	n.d.	0
			cy	-	-	n.d.	n.d.	n.d.	0	n.d.	n.d.	n.d.	0
			de	-	-	n.d.	n.d.	n.d.	0	n.d.	n.d.	n.d.	0
			c	-	-	n.d.	n.d.	n.d.	0	n.d.	n.d.	n.d.	0
			e	-	-	n.d.	n.d.	n.d.	0	n.d.	n.d.	n.d.	0
4c,e	*ttg2-1* (L*er*)	non-extracted hydrolysed	ta	-	-	n.d.	n.d.	n.d.	0	n.d.	n.d.	n.d.	0
			ka	+	+	0.763	0.398	52.24	1.1	1.000	0.000	0.00	1,0
			qu	+	+	2.320	0.438	18.89	0.5	5.519	1.321	23.94	0,8
			is	+	+	0.419	0.109	26.05	0.9	0.557	0.256	45.96	0,8
			pe	-	-	n.d.	n.d.	n.d.	0	n.d.	n.d.	n.d.	0
			cy	-	-	n.d.	n.d.	n.d.	0	n.d.	n.d.	n.d.	0
			de	-	-	n.d.	n.d.	n.d.	0	n.d.	n.d.	n.d.	0
			c	-	-	n.d.	n.d.	n.d.	0	n.d.	n.d.	n.d.	0
			e	-	-	n.d.	n.d.	n.d.	0	n.d.	n.d.	n.d.	0
4c,e	*pap1-D* (Col-0)	non-extracted hydrolysed	ta	+	+	0.350	0.029	8.16	0.9	0.559	0.287	51.34	0,5
			ka	+	-	0.185	0.082	44.20	0.5	1.000	0.000	0.00	1,0
			qu	+	+	1.790	0.504	28.13	0.4	14.914	2.743	18.39	1,2
			is	+	+	0.334	0.143	42.92	0.7	0.988	0.233	23.58	0,8
			pe	+	+	0.252	0.021	8.42	0.8	0.409	0.171	41.81	0,5
			cy	+	+	45.852	2.268	4.95	0.8	90.868	44.150	48.59	0,6
			de	+	+	0.651	0.028	4.24	0.9	1.165	0.607	52.10	0,6

Table S12. Data for Figure 4c-e

Figure	Name	Sample type	Substance	LOD passed	LOQ passed	Normalized to internal standard: Normalized response	STDEV	RSD [%]	Fold difference to wt	Normalized to kaempferol: Normalized response	STDEV	RSD [%]	Fold difference to wt
4c,e	*pap1-D* (Col-0)	non-extracted hydrolysed	c	+	+	12.837	0.524	4.08	1.0	18.343	9.105	49.64	0,6
			e	+	+	2.238	0.174	7.76	0.7	3.623	1.411	38.95	0,4
4c,e	*myb*RNAi (Col-0)	non-extracted hydrolysed	ta	+	+	0.285	0.040	14.02	0.7	0.451	0.279	61.86	0,4
			ka	+	-	0.299	0.105	35.24	0.8	1.000	0.000	0.00	1,0
			qu	+	+	1.781	0.557	31.25	0.4	2.893	1.435	49.60	0,2
			is	+	+	0.311	0.124	40.02	0.7	0.595	0.119	20.00	0.5
			pe	+	+	0.250	0.066	26.46	0.8	0.872	0.199	22.83	1.1
			cy	+	+	42.790	6.397	14.95	0.7	59.077	34.604	58.57	0.4
			de	+	+	0.524	0.063	12.07	0.7	1.911	0.635	33.22	1.0
			c	+	+	8.446	2.938	34.79	0.7	7.118	5.453	76.61	0.2
			e	+	+	1.717	1.013	58.99	0.5	1.076	0.849	78.90	0,1
4c,e	*hy5-215* (Col-0)	non-extracted hydrolysed	ta	+	+	0.218	0.040	18.27	0.5	0.451	0.279	61.92	0.4
			ka	+	+	0.624	0.390	62.47	1.6	1.000	0.000	0.00	1.0
			qu	+	+	1.511	0.450	29.80	0.3	2.893	1.435	49.60	0.2
			is	+	+	0.344	0.155	44.91	0.7	0.595	0.119	20.03	0.5
			pe	+	-	0.170	0.038	22.21	0.6	0.360	0.221	61.34	0.4
			cy	+	+	28.990	5.106	17.61	0.5	59.077	34.604	58.57	0.4
			de	+	-	0.293	0.064	21.79	0.4	0.626	0.433	69.21	0.3
			c	+	+	3.700	1.958	52.91	0.3	7.118	5.453	76.62	0.2
			e	+	+	0.617	0.472	76.58	0.2	1.076	0.849	78.83	0.1
4c,e	*cop1-6* (Col-0)	non-extracted hydrolysed	ta	+	+	0.317	0.042	13.25	0.8	0.559	0.287	51.40	0.5
			ka	+	+	0.682	0.337	49.40	1.8	1.000	0.000	0.00	1.0
			qu	+	+	10.529	6.369	60.49	2.3	14.914	2.743	18.39	1.2
			is	+	+	0.613	0.159	25.89	1.3	0.988	0.233	23.62	0.8
			pe	+	+	0.235	0.030	12.55	0.8	0.409	0.171	41.88	0.5
			cy	+	+	50.911	3.040	5.97	0.8	90.868	44.150	48.59	0.6
			de	+	+	0.657	0.066	10.05	0.9	1.165	0.607	52.12	0.6
			c	+	+	10.311	0.510	4.94	0.8	18.343	9.105	49.64	0.5
			e	+	+	2.102	0.282	13.42	0.7	3.623	1.411	38.95	0.4
4c,e	Col-0	non-extracted hydrolysed	ta	+	+	0.401	0.053	13.23	1	1.074	0.254	23.65	1
			ka	+	+	0.390	0.091	23.41	1	1.000	0.000	0.00	1
			qu	+	+	4.675	1.125	24.07	1	12.177	2.027	16.65	1
			is	+	+	0.472	0.027	5.74	1	1.274	0.333	26.14	1
			pe	+	+	0.297	0.048	16.33	1	0.805	0.269	33.42	1

Table S12. Data for Figure 4c-e

						Normalized to internal standard				Normalized to kaempferol			
**Figure**	**Name**	**Sample type**	**Substance**	**LOD passed**	**LOQ passed**	**Normalized response**	**STDEV**	**RSD [%]**	**Fold difference to wt**	**Normalized response**	**STDEV**	**RSD [%]**	**Fold difference to wt**
4c,e	Col-0	non-extracted hydrolysed	cy	+	+	61.057	6.141	10.06	1	163.953	39.548	24.12	1
			de	+	+	0.757	0.220	29.12	1	1.987	0.496	24.96	1
			c	+	+	12.838	3.281	25.55	1	33.486	6.427	19.19	1
			e	+	+	3.218	0.578	17.97	1	8.593	2.192	25.51	1
4c-e	L*er*	non-extracted hydrolysed	ta	+	+	0.375	0.077	20.58	1	0.581	0.156	26.85	1
			ka	+	+	0.695	0.255	36.64	1	1.000	0.000	0.00	1
			qu	+	+	4.460	1.084	24.30	1	6.916	2.283	33.01	1
			is	+	+	0.456	0.166	36.36	1	0.661	0.133	20.12	1
			pe	+	+	0.237	0.054	22.87	1	0.364	0.089	24.45	1
			cy	+	+	52.486	10.856	20.68	1	79.717	16.006	20.08	1
			de	+	+	0.659	0.125	18.95	1	1.014	0.246	24.26	1
			c	+	+	11.984	3.766	31.42	1	17.607	2.634	14.96	1
			e	+	+	2.694	0.914	33.92	1	3.946	0.655	16.60	1
4c,e	Ws-2	non-extracted hydrolysed	ta	+	+	0.333	0.068	20.28	1	0.716	0.231	32.26	1
			ka	+	+	0.489	0.107	21.97	1	1.000	0.000	0.00	1
			qu	+	+	6.439	1.879	29.18	1	13.397	3.317	24.76	1
			is	+	+	0.307	0.072	23.53	1	0.647	0.153	23.65	1
			pe	+	+	0.051	0.012	24.04	1	0.107	0.024	22.43	1
			cy	+	+	55.966	9.586	17.13	1	119.926	36.067	30.07	1
			de	+	+	0.583	0.110	18.82	1	1.230	0.298	24.23	1
			c	+	+	6.630	1.992	30.04	1	14.407	5.967	41.42	1
			e	+	+	1.162	0.604	52.01	1	2.548	1.439	56.48	1

For experimental procedures see methods. text and Fig. 4. Note that peak areas and statistics are only provided, when at least one mutant/wildtype passed LOQ for the respective substance in the respective sample type and in case of kaempferol ratios. when kaempferol was detectable. For mutants, values are only determined when the respective wildtype is above LOQ (with one exception. see below). If these criteria are not given, the values are not determined (n.d.). For pelargonidin in extracted hydrolysed samples, only values for *tt7-1* and L*er* are given as detection is due to release from the internal standard and the high levels of this substance were show in Figure 4d. *Values for cyanidin in non-extracted hydrolysed samples for *tt7-1* are not determined as this was shown to be due to conversion from pelargonidin.

"Normalized response" = normalized to the respective internal standard or to kaempferol as indicated above the column. Fold differences are calculated relative to the respective wildtypes. For kaempferol ratios for each samples and substance the ratios were calculated. Means, STDEVs and RSDs for these ratios are given here.

"n.d." = not determined. "Name": The name of the wildtype or mutant is give. In brackets: the background of the respective mutant is given. "ta" = taxifolin. "ka" = kaempferol. "qu" = quercetin. "is" = isorhamnetin. "pe" = pelargonidin. "cy" = cyaniding. "de" = delphinidin. "c" = catechin. "e" = epicatechin. "pB" = procyanidin B2. For LOD/LOQ see methods.

			compared to geno2 (*P*-value)										
			tt3-1	***ttg1-1***	***tt8-3***	***tt2-1***	***pap1-D***	***myb* RNAi**	***hy5-215***	***cop1-6***	**Col-0**	**L*er***	**Ws-2**
**sample type**	**substance**	**geno1**											
extracted non-hydrolysed	e	***pap1-D***						6.31E-01	2.90E-01	8.06E-02	9.34E-01		
		***myb* RNAi**							2.31E-01	7.43E-02	7.36E-01		
		hy5-215								9.92E-02	3.23E-01		
		cop1-6									7.95E-02		
		**Col-0**										1.04E-02	5.97E-03
		**L*er***											1.54E-01
	pB	***pap1-D***						2.52E-01	1.54E-02	6.66E-02	2.87E-01		
		***myb* RNAi**							4.16E-02	8.71E-02	1.28E-01		
		hy5-215								4.99E-02	8.57E-03		
		cop1-6									6.03E-02		
		**Col-0**										8.54E-03	3.08E-03
		**L*er***											5.25E-03
extracted hydrolysed	ka	***tt7-1***	6.34E-03	6.08E-03	6.26E-03	6.03E-03	5.32E-03	5.33E-03	6.27E-03	5.86E-03	5.42E-03	6.05E-03	6.08E-03
		tt3-1		2.69E-01	4.66E-01	2.48E-01	8.34E-04	4.58E-07	7.01E-01	5.38E-01		1.26E-02	
		ttg1-1			4.70E-01	8.86E-01	5.89E-03	6.36E-03	4.33E-01	9.20E-01		7.49E-01	
		tt8-3				4.13E-01	7.37E-04	4.90E-05	8.66E-01	6.59E-01			4.45E-01
		tt2-1					1.13E-02	1.18E-02	3.81E-01	9.87E-01		9.10E-01	
		pap1-D						9.20E-01	6.57E-04	1.22E-01	5.69E-01		
		***myb* RNAi**							4.28E-04	1.14E-01	2.60E-01		
		hy5-215								6.24E-01	3.41E-04		
		cop1-6									1.60E-01		
		**Col-0**										2.53E-04	6.51E-03
		**L*er***											7.37E-01
	qu	***tt3-1***		1.50E-03	5.35E-01	1.94E-03	3.56E-06	1.16E-05	4.10E-07	6.75E-05		4.13E-01	
		ttg1-1			1.94E-03	3.72E-01	4.83E-01	7.82E-01	1.90E-02	1.33E-06		4.29E-03	
		tt8-3				4.81E-03	1.79E-03	3.31E-03	3.33E-04	1.10E-04			1.11E-01
		tt2-1					7.56E-02	3.59E-01	2.44E-03	1.85E-06		1.16E-02	
		pap1-D						5.93E-02	1.88E-03	1.32E-05	5.70E-03		
		***myb* RNAi**							2.43E-04	1.61E-05	1.74E-02		
		hy5-215								2.59E-06	4.47E-04		
		cop1-6									6.38E-06		
		**Col-0**										2.51E-01	1.32E-03
		**L*er***											7.65E-03
	is	***tt3-1***		1.52E-04	4.71E-04	1.97E-04	3.43E-03	1.75E-04	4.30E-04	2.35E-02		1.50E-03	
		ttg1-1			1.33E-03	1.72E-03	5.25E-03	9.67E-04	6.43E-05	1.98E-03		1.88E-04	
		tt8-3				3.60E-01	6.55E-01	1.49E-01	1.42E-01	1.00E-01			9.53E-01
		tt2-1					2.74E-01	5.72E-01	5.91E-01	3.54E-02		5.61E-02	
		pap1-D						1.56E-01	1.61E-01	2.63E-01	3.17E-01		
		***myb* RNAi**							8.96E-01	2.14E-02	2.66E-01		
		hy5-215								2.48E-02	1.76E-01		
		cop1-6									4.41E-02		
		**Col-0**										3.40E-02	4.97E-01
		**L*er***											3.37E-01
	pe	***tt7-1***										2.38E-03	
	cy	***pap1-D***						2.44E-01	8.07E-04	1.31E-03	6.21E-03		
		***myb* RNAi**							4.78E-03	6.04E-04	5.65E-02		
		hy5-215								3.43E-04	6.19E-02		
		cop1-6									3.13E-04		
		**Col-0**										5.88E-06	7.89E-05
		**L*er***											1.28E-03
	c	***pap1-D***						9.38E-01	4.22E-04	9.96E-03	1.07E-02		
		***myb* RNAi**							4.17E-02	8.57E-03	8.68E-03		
		hy5-215								5.66E-03	3.13E-03		
		cop1-6									6.25E-02		
		**Col-0**										1.31E-02	1.41E-03
		**L*er***											2.60E-02
	e	***pap1-D***						7.55E-01	2.52E-01	1.50E-02	1.31E-01		
		***myb* RNAi**							1.90E-01	1.23E-02	1.57E-02		
		hy5-215								8.87E-03	4.49E-04		
		cop1-6									5.64E-02		
		**Col-0**										5.51E-05	7.26E-03
		**L*er***											4.95E-02
	ta	***pap1-D***						2.54E-02	6.95E-04	2.13E-01	1.11E-01		
		***myb* RNAi**							2.90E-02	2.47E-01	5.18E-03		
		hy5-215								4.99E-03	3.63E-04		
		cop1-6									2.57E-02		
		**Col-0**										5.59E-01	1.18E-01
		**L*er***											3.87E-01
	ka	***tt7-1***	1.11E-03	1.12E-03	1.11E-03	1.07E-03	9.84E-04	1.02E-03	1.03E-03	1.07E-03	1.05E-03	1.11E-03	1.08E-03
		tt3-1		9.40E-01	6.32E-01	7.42E-01	1.06E-02	2.69E-02	7.68E-01	9.71E-01		9.72E-01	
		ttg1-1			6.42E-01	6.87E-01	4.20E-03	1.24E-02	7.98E-01	9.80E-01		9.08E-01	
		tt8-3				4.87E-01	3.18E-03	1.24E-02	9.72E-01	7.17E-01			2.13E-01
		tt2-1					2.98E-02	5.82E-02	5.94E-01	7.39E-01		7.59E-01	
		pap1-D						1.10E-01	6.41E-02	2.76E-02	9.70E-03		
		***myb* RNAi**							1.37E-01	6.20E-02	1.85E-01		
		hy5-215								8.09E-01	2.54E-01		

		cop1-6								1.25E-01		
		**Col-0**									5.28E-02	1.56E-01
		**L*er***										1.52E-01
	qu	***tt3-1***	1.26E-02	9.24E-01	3.79E-02	8.71E-03	7.57E-03	3.46E-03	6.63E-02		1.24E-01	
		ttg1-1		5.37E-02	2.65E-01	6.18E-01	5.95E-01	1.67E-01	3.94E-02		4.06E-03	
		tt8-3			1.21E-01	3.72E-02	3.57E-02	2.02E-02	6.43E-02			1.76E-02
		tt2-1				1.47E-01	1.29E-01	2.06E-02	4.47E-02		8.42E-03	
		pap1-D					9.80E-01	4.19E-01	3.70E-02	2.43E-03		
		***myb* RNAi**						4.25E-01	3.69E-02	2.28E-03		
		hy5-215							3.38E-02	1.77E-03		
		cop1-6								1.09E-01		
		**Col-0**									7.67E-01	1.18E-01
		**L*er***										1.09E-01
non-extracted hydrolysed	is	***tt3-1***	1.12E-03	4.20E-03	1.12E-02	5.77E-03	3.36E-03	5.29E-03	1.59E-01		2.20E-02	
		ttg1-1		2.89E-03	1.03E-03	3.45E-02	1.18E-02	1.69E-02	1.04E-03		5.70E-03	
		tt8-3			1.87E-01	9.06E-01	8.57E-01	8.09E-01	1.13E-02			7.71E-01
		tt2-1				3.71E-01	1.83E-01	4.09E-01	5.84E-02		6.90E-01	
		pap1-D					8.04E-01	9.21E-01	2.87E-02	1.49E-01		
		***myb* RNAi**						7.14E-01	1.09E-02	4.21E-02		
		hy5-215							2.68E-02	1.38E-01		
		cop1-6								1.19E-01		
		**Col-0**									8.33E-01	4.71E-03
		**L*er***										1.21E-01
	pe	***tt7-1***				4.06E-05	4.04E-05	4.01E-05	4.05E-05	4.08E-05	4.04E-05	3.95E-05
		pap1-D					9.68E-01	5.36E-03	3.64E-01	1.15E-01		
		***myb* RNAi**						5.36E-02	6.59E-01	2.47E-01		
		hy5-215							1.67E-02	2.00E-03		
		cop1-6								4.85E-02		
		**Col-0**									1.06E-01	2.00E-04
		**Ler**										1.15E-03
	cy	***pap1-D***					3.64E-01	6.84E-04	2.44E-02	3.18E-03		
		***myb* RNAi**						5.96E-03	4.45E-02	1.75E-03		
		hy5-215							1.11E-04	2.32E-05		
		cop1-6								1.68E-02		
		**Col-0**									1.73E-01	3.51E-01
		**L*er***										6.06E-01
	de	***pap1-D***					1.24E-04	4.17E-05	8.64E-01	3.44E-01		
		***myb* RNAi**						4.25E-04	1.17E-02	7.61E-02		
		hy5-215							2.07E-05	7.39E-03		
		cop1-6								3.77E-01		
		**Col-0**									4.17E-01	1.67E-01
		**L*er***										3.41E-01
	c	***pap1-D***					2.74E-02	2.42E-04	2.42E-04	1.00E+00		
		***myb* RNAi**						1.99E-02	2.31E-01	5.67E-02		
		hy5-215							1.13E-03	1.34E-03		
		cop1-6								1.61E-01		
		**Col-0**									7.12E-01	9.46E-03
		**L*er***										3.03E-02
	e	***pap1-D***					3.18E-01	6.91E-04	4.07E-01	1.64E-02		
		***myb* RNAi**						7.26E-02	4.53E-01	2.64E-02		
		hy5-215							6.78E-04	6.53E-05		
		cop1-6								8.74E-03		
		**Col-0**									3.15E-01	5.78E-04
		**L*er***										1.69E-02

geno1, geno2: genotype 1 and genotype 2 used for Welch-test.

P-values from Welch-test for normalized responses (with the respective internal standard) used in Figure 4c,d and Table S12.

“ta” = taxifolin, “ka” = kaempferol, “qu” = quercetin, “is” = isorhamnetin, “pe” = pelargonidin, “cy” = cyaniding, “de” = delphinidin, “c” = catechin, “e” = epicatechin, “pB” = procyanidin B2.
