# Supplementary material for: A fast and simple LC-MS-based characterization of the flavonoid biosynthesis pathway for few seed(ling)s
Source: BMC Plant Biol. 2016 Sep 1;16(1):190. doi: 10.1186/s12870-016-0880-7 (PMC5007998; doi:10.1186/s12870-016-0880-7)
Supplement: Additional file 8: Figure S11. — Adaptation of the method to seedlings. Hydrolysis of main glycosylated flavonoids from seedling extracts; and Table S14. Data for Fig. 6. (PDF 445 kb) [file 12870_2016_880_MOESM8_ESM.pdf]

Additional_file_8

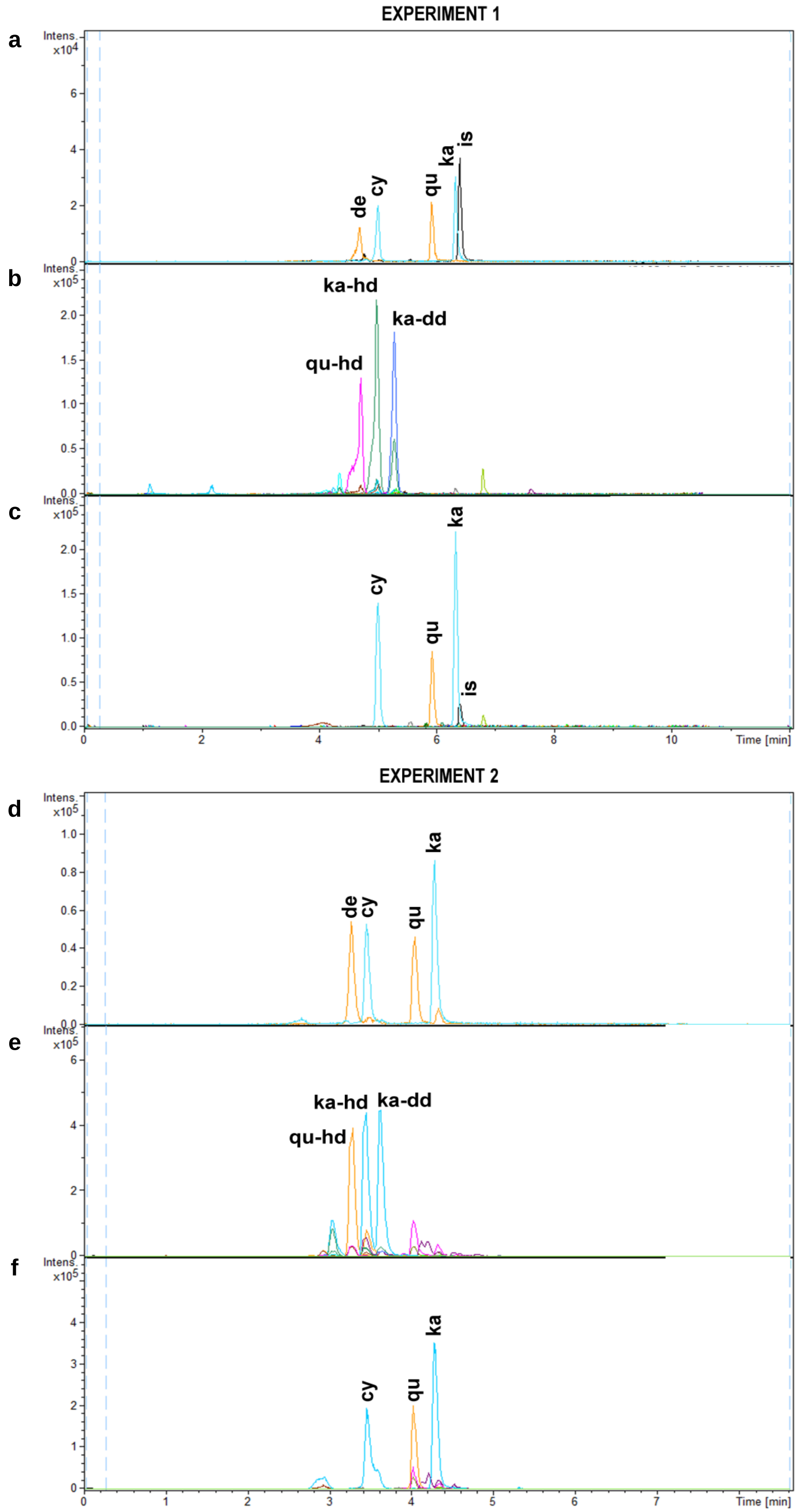

Figure S11: Hydrolysis of main glycosylated flavonoids from seedling extracts. In two experiments (**a-c** and **d-f**), extracted non-hydrolysed samples from ten (b) and 15 (e) *A. thaliana* Col-0 seedlings with three replicates were subjected to hydrolytic conditions and analysed with the LC-ESI-MS-QTRAP(MRM) (a-c) and LC-ESI-MS-QTOF(pseudoMS^3) setup (d-f). Note that we reduced the gradient in EXPERIMENT 2 even further. Shown are EICs for all *m/z* values documented in Table S3,6 covering all glycosylated flavonoids analyzed in Fig. 1c and all substances shown in Fig. 1b which theoretically can be present in plants. Reference EICs for the relevant flavonoids are given for each experiment (a,d).
EXPERIMENT 1: MeOH:HCl conc. (95:5) was replaced with MeOH:FA (99:1). Samples were either diluted without additional acid and treatment (b) or hydrolysed for 60 min at a final concentration of 5% HCl conc. (c). Samples shown in b and c originate from the same seedlings.
EXPERIMENT 2: MeOH:HClconc. (95:5) was replaced with MeOH:FA (99:1). Heat was either applied (60min, sample f) or not applied (e).

Note that the kaempferol-based glycosylated flavonoid elutes close to cyanidin. Therefore care needs to be taken when analysing soluble samples with MRMs due to possible in-source fragmentation of glycosylated flavonoids. In this study only hydrolysed samples are analysed when using MRMs.

de: delphinidin, cy: cyanidin, qu: quercetin, ka: kaempferol, is: isorhamnetin, h: modified with an hexose, d: modified with a deoxy-hexose.

Table S14. Data for Figure 5

Figure	Name	Hydrolysis [min]	No. of seedlings	Dilution	Substance	LOD passed	LOQ passed	Normalized response	STDEV	RSD [%]	N	Compared to	*P*-value	Linearity (r^2)
5a	Col-0	30	10	-	na	n.d.	+	0.019	0.001	6.4	5	60 min hydrolysis	0.147	n.d.
					ta		+	0.012	0.002	20.0			0.034	
					ka		+	4.723	0.236	5.0			0.002	
					qu		+	1.445	0.167	11.6			0.177	
					is		+	0.622	0.082	13.2			0.151	
					my		-	n.d.	n.d.	n.d.			n.d.	
					pe		-	n.d.	n.d.	n.d.			n.d.	
					cy		+	0.813	0.169	20.8			0.002	
					de		-	n.d.	n.d.	n.d.			n.d.	
					c		-	n.d.	n.d.	n.d.			n.d.	
					e		-	n.d.	n.d.	n.d.			n.d.	
					pB		-	n.d.	n.d.	n.d.			n.d.	
5a	Col-0	60	10	-	na	n.d.	+	0.021	0.002	10.6	5	90 min hydrolysis	0.159	n.d.
					ta		+	0.015	0.001	8.1			0.083	
					ka		+	5.366	0.198	3.7			0.070	
					qu		+	1.660	0.271	16.3			0.043	
					is		+	0.701	0.075	10.7			0.020	
					my		-	n.d.	n.d.	n.d.			n.d.	
					pe		-	n.d.	n.d.	n.d.			n.d.	
					cy		+	1.373	0.211	15.4			0.743	
					de		-	n.d.	n.d.	n.d.			n.d.	
					c		-	n.d.	n.d.	n.d.			n.d.	
					e		-	n.d.	n.d.	n.d.			n.d.	
					pB		-	n.d.	n.d.	n.d.			n.d.	
5a	Col-0	90	10	-	na	n.d.	+	0.025	0.005	18.1	5	30 min hydrolysis	0,049	n.d.
					ta		+	0.018	0.003	15.9			0,006	
					ka		+	5.594	0.135	2.4			0,0003	
					qu		+	1.021	0.494	48.4			0.130	
					is		+	0.497	0.129	25.9			0.110	
					my		-	n.d.	n.d.	n.d.			n.d.	
					pe		-	n.d.	n.d.	n.d.			n.d.	
					cy		+	1.409	0.109	7.7			0.0003	
					de		-	n.d.	n.d.	n.d.			n.d.	
					c		-	n.d.	n.d.	n.d.			n.d.	
					e		-	n.d.	n.d.	n.d.			n.d.	

Table S14. Data for Figure 5

Figure	Name	Hydrolysis [min]	No. of seedlings	Dilution	Substance	LOD passed	LOQ passed	Normalized response	STDEV	RSD [%]	N	Compared to	*P*-value	Linearity (r²)
5a	Col-0	90	10	-	pB	n.d.	-	n.d.	n.d.	n.d.	5	see above	n.d.	n.d.
					na		+	2.23	0.37	16.7		1/5/10*	<0.01	
					ta		+	1.42	0.22	15.5		5/10/15*	<0.01	
					ka		+	268.18	23.25	8.7		1/5/10/15	<0.01	
					qu		+	96.51	28.39	29.4		1/5*	<0.01	
					is		+	40.73	6.15	15.1		1/5/10*	<0.01	
5b-d	Col-0	60	20	-	my	n.d.	-	n.d.	n.d.	n.d.	6	n.d.	n.d.	substance r²
					pe		-	n.d.	n.d.	n.d.		n.d.	n.d.	
					cy		+	99.90	16.01	16.0		1/5/10/15	<0.05	
					de		-	n.d.	n.d.	n.d.		n.d.	n.d.	
					c		-	n.d.	n.d.	n.d.		n.d.	n.d.	
					e		-	n.d.	n.d.	n.d.		n.d.	n.d.	
					pB		-	n.d.	n.d.	n.d.		n.d.	n.d.	
					na		+	1.52	0.67	44.1		1*	<0.05	na 0.90
					ta		+	0.99	0.07	6.7		5/10*	<0.01	
					ka		+	213.06	12.08	5.7		1/5/10/20	<0.01	
					qu		+	86.02	24.54	28.5		1/5*	<0.01	
					is		+	33.33	5.44	16.3		1/5/10*	<0.05	ta 0.99
5b-d	Col-0	60	15	-	my	n.d.	-	n.d.	n.d.	n.d.	5	n.d.	n.d.	
					pe		-	n.d.	n.d.	n.d.		n.d.	n.d.	
					cy		+	75.58	3.97	5.2		1/5/10/20	<0.05	
					de		-	n.d.	n.d.	n.d.		n.d.	n.d.	ka 0.96
					c		-	n.d.	n.d.	n.d.		n.d.	n.d.	
					e		-	n.d.	n.d.	n.d.		n.d.	n.d.	
					pB		-	n.d.	n.d.	n.d.		n.d.	n.d.	
					na		+	1.52	0.22	14.2		1/5/20*	<0.05	qu 0.90
					ta		+	0.67	0.06	8.5		1/5/15/20	<0.001	
					ka		+	159.33	12.46	7.8		1/5/15/20	<0.001	
					qu		+	73.00	5.70	7.8		1/5*	<0.001	
5b-d	Col-0	60	10	-	is	n.d.	+	25.45	2.20	8.7	5	1/5/15/20	<0.05	is 0.97
					my		-	n.d.	n.d.	n.d.		n.d.	n.d.	
					pe		-	n.d.	n.d.	n.d.		n.d.	n.d.	
					cy		+	57.60	3.79	6.6		1/5/15/20	<0.001	
					de		-	n.d.	n.d.	n.d.		n.d.	n.d.	cy 0.99
					c		-	n.d.	n.d.	n.d.		n.d.	n.d.	

Table S14. Data for Figure 5

Figure	Name	Hydrolysis [min]	No. of seedlings	Dilution	Substance	LOD passed	LOQ passed	Normalized response	STDEV	RSD [%]	N	Compared to	*P*-value	Linearity (r^2)
5b-d	Col-0	60	10	-	e	n.d.	-	n.d.	n.d.	n.d.	5	n.d.	n.d.	see above
					pB		-	n.d.	n.d.	n.d.		n.d.	n.d.	
5b-d	Col-0	60	5	-	na	n.d.	+	0.91	0.37	41.0	5	1/10/20*	<0.05	
					ta		+	0.31	0.06	19.1		10/15/20*	<0.001	
					ka		+	119.42	7.22	6.0		1/10/15/20	<0.001	
					qu		+	28.31	5.39	19.0		1/10/15/20	<0.01	
					is		+	13.25	1.63	12.3		1/10/15/20	<0.001	
					my		-	n.d.	n.d.	n.d.		n.d.	n.d.	
					pe		-	n.d.	n.d.	n.d.		n.d.	n.d.	
					cy		+	28.47	4.61	16.2		1/10/15/20	<0.001	
					de		-	n.d.	n.d.	n.d.		n.d.	n.d.	
					c		-	n.d.	n.d.	n.d.		n.d.	n.d.	
					e		-	n.d.	n.d.	n.d.		n.d.	n.d.	
					pB		-	n.d.	n.d.	n.d.		n.d.	n.d.	
5b-d	Col-0	60	1	-	na	n.d.	+	0.16	0.07	46.2	5	5/10/15/20	<0.05	
					ta		-	n.d.	n.d.	n.d.		n.d.	n.d.	
					ka		+	31.18	4.45	14.3		5/10/15/20	<0.001	
					qu		+	4.27	0.87	20.2		5/10/15/20	<0.01	
					is		+	1.81	0.29	15.8		5/10/15/20	<0.001	
					my		-	n.d.	n.d.	n.d.		n.d.	n.d.	
					pe		-	n.d.	n.d.	n.d.		n.d.	n.d.	
					cy		+	7.26	1.37	18.9		5/10/15/20	<0.001	
					de		-	n.d.	n.d.	n.d.		n.d.	n.d.	
					c		-	n.d.	n.d.	n.d.		n.d.	n.d.	
					e		-	n.d.	n.d.	n.d.		n.d.	n.d.	
					pB		-	n.d.	n.d.	n.d.		n.d.	n.d.	
5e	Col-0	60	10	-	na	+	+	1.75	0.49	28.31	10	*hy5-215*	0.144	n.d.
					ta	+	+	0.62	0.08	12.88			0.014	
					ka	+	+	167.02	12.11	7.25			$5.6x10^{-11}$	
					qu	+	+	72.05	7.15	9.92			$1.9x10^{-06}$	
					is	+	+	25.03	2.04	8.16			0.002	
					my	-	-	n.d.	n.d.	n.d.			n.d.	
					pe	+	-	n.d.	n.d.	n.d.			n.d.	
					cy	+	+	57.19	6.57	11.48			$2.3x10^{-10}$	
					de	-	-	n.d.	n.d.	n.d.			n.d.	

Table S14. Data for Figure 5

Figure	Name	Hydrolysis [min]	No. of seedlings	Dilution	Substance	LOD passed	LOQ passed	Normalized response	STDEV	RSD [%]	N	Compared to	*P*-value	Linearity (r²)
				-	c	-	-	n.d.	n.d.	n.d.	10	*hy5-215*	n.d.	n.d.
					e	-	-	n.d.	n.d.	n.d.			n.d.	
					pB	-	-	n.d.	n.d.	n.d.			n.d.	
5e	*hy5-215* (Col-0)	60	10	-	na	+	+	4.33	1.93	44.65	3	*myb*RNAi	0.0777	n.d.
					ta	+	+	0.31	0.09	30.12			0.5347	
					ka	+	+	58.29	3.53	6.05			0.0015	
					qu	+	+	6.20	4.35	70.13			0.0005	
					is	+	+	6.58	2.53	38.53			0.0026	
					my	-	-	n.d.	n.d.	n.d.			n.d.	
					pe	+	-	n.d.	n.d.	n.d.			n.d.	
					cy	+	+	5.69	1.10	19.34			0.0437	
					de	-	-	n.d.	n.d.	n.d.			n.d.	
					c	-	-	n.d.	n.d.	n.d.			n.d.	
					e	-	-	n.d.	n.d.	n.d.			n.d.	
					pB	-	-	n.d.	n.d.	n.d.			n.d.	
5e	*myb*RNAi (Col-0)	60	10	-	na	+	+	0.57	0.07	12.10	3	Col-0	2.6×10^{-05}	n.d.
					ta	+	+	0.27	0.03	9.70			1.9×10^{-07}	
					ka	+	+	170.11	10.95	6.44			0.70	
					qu	+	+	62.40	6.55	10.50			0.10	
					is	+	+	24.61	3.44	14.00			0.86	
					my	-	-	n.d.	n.d.	n.d.			n.d.	
					pe	+	-	n.d.	n.d.	n.d.			n.d.	
					cy	+	+	13.60	3.35	24.63			9.7×10^{-07}	
					de	-	-	n.d.	n.d.	n.d.			n.d.	
					c	-	-	n.d.	n.d.	n.d.			n.d.	
					e	-	-	n.d.	n.d.	n.d.			n.d.	
					pB	-	-	n.d.	n.d.	n.d.			n.d.	
5e	*ttg1-1* (L*er*)	60	10	-	na	+	+	0.64	0.35	55.35	3	*tt3-1*	0.470	n.d.
					ta	+	+	0.56	0.07	12.04			0.046	
					ka	+	+	224.53	21.50	9.58			0.561	
					qu	+	+	104.95	11.95	11.39			0.058	
					is	+	+	2.16	0.09	4.38			0.016	
					my	-	-	n.d.	n.d.	n.d.			n.d.	
					pe	+	-	n.d.	n.d.	n.d.			n.d.	
					cy	-	-	n.d.	n.d.	n.d.			n.d.	

Table S14. Data for Figure 5

Figure	Name	Hydrolysis [min]	No. of seedlings	Dilution	Substance	LOD passed	LOQ passed	Normalized response	STDEV	RSD [%]	N	Compared to	*P*-value	Linearity (r^2)
5e	*ttg1-1* (L*er*)	60	10	-	de	-	-	n.d.	n.d.	n.d.	3	*tt3-1*	n.d.	n.d.
					c	-	-	n.d.	n.d.	n.d.			n.d.	
					e	-	-	n.d.	n.d.	n.d.			n.d.	
					pB	-	-	n.d.	n.d.	n.d.			n.d.	
5e	*tt3-1* (L*er*)	60	10	-	na	+	+	0.83	0.16	19.27	3	see above		n.d.
					ta	+	+	25.66	9.65	37.59				
					ka	+	+	243.89	46.33	19.00				
					qu	+	+	177.16	35.10	19.82				
					is	+	+	101.43	22.09	21.78				
					my	-	-	n.d.	n.d.	n.d.				
					pe	+	-	n.d.	n.d.	n.d.				
					cy	-	-	n.d.	n.d.	n.d.				
					de	-	-	n.d.	n.d.	n.d.				
					c	-	-	n.d.	n.d.	n.d.				
					e	-	-	n.d.	n.d.	n.d.				
					pB	-	-	n.d.	n.d.	n.d.				
5e	*cop1-6* (Col-0)	60	10	1:20	na	+	+	2.64	0.19	7.3	3	*pap1-D*	0.0005	n.d.
					ta	+	+	1.98	0.06	3.2			0.0030	
					ka	+	+	793.97	44.22	5.6			0.0001	
					qu	+	+	368.11	94.61	25.7			0.0176	
					is	+	+	133.23	10.40	7.8			0.0005	
					my	-	-	n.d.	n.d.	n.d.			n.d.	
					pe	+	-	n.d.	n.d.	n.d.			n.d.	
					cy	+	+	341.67	60.61	17.7			0.0820	
					de	+	-	n.d.	n.d.	n.d.			n.d.	
					c	-	-	n.d.	n.d.	n.d.			n.d.	
					e	-	-	n.d.	n.d.	n.d.			n.d.	
					pB	-	-	n.d.	n.d.	n.d.			n.d.	
5e	*pap1-D* (Col-0)	60	10	1:20	na	+	-	1.06	0.19	18.0	3	see above		n.d.
					ta	+	-	0.76	0.17	22.7				
					ka	+	+	264.99	34.95	13.2				
					qu	+	+	41.10	34.01	82.7				
					is	+	+	16.36	14.06	85.9				
					my	-	-	n.d.	n.d.	n.d.				
					pe	+	-	n.d.	n.d.	n.d.				

Table S14. Data for Figure 5

<table>
<tr><th>Figure</th><th>Name</th><th>Hydrolysis [min]</th><th>No. of seedlings</th><th>dilution</th><th>Substance</th><th>LOD passed</th><th>LOQ passed</th><th>Normalized response</th><th>STDEV</th><th>RSD [%]</th><th>N</th><th>Compared to</th><th>P-value</th><th>Linearity (r²)</th></tr>
<tr><td rowspan="5">5e</td><td rowspan="5">pap1-D (Col-0)</td><td rowspan="5">60</td><td rowspan="5">10</td><td rowspan="5">1:20</td><td>cy</td><td>+</td><td>+</td><td>232.86</td><td>15.09</td><td>6.5</td><td rowspan="5">3</td><td rowspan="5" colspan="2">see above</td><td rowspan="5">n.d.</td></tr>
<tr><td>de</td><td>-</td><td>-</td><td>n.d.</td><td>n.d.</td><td>n.d.</td></tr>
<tr><td>c</td><td>-</td><td>-</td><td>n.d.</td><td>n.d.</td><td>n.d.</td></tr>
<tr><td>e</td><td>-</td><td>-</td><td>n.d.</td><td>n.d.</td><td>n.d.</td></tr>
<tr><td>pB</td><td>-</td><td>-</td><td>n.d.</td><td>n.d.</td><td>n.d.</td></tr>
<tr><td rowspan="4">5b-d</td><td rowspan="4">Col-0</td><td rowspan="4">60</td><td>20</td><td>1:13.5</td><td rowspan="4">ka</td><td>+</td><td>+</td><td>408.46</td><td>36.59</td><td>n.d.</td><td>6</td><td>5/10/15[#]</td><td><0.05</td><td rowspan="3">substance | r²</td></tr>
<tr><td>15</td><td>1:9</td><td>+</td><td>+</td><td>349.94</td><td>21.82</td><td>n.d.</td><td>5</td><td>5/10/20[#]</td><td><0.05</td></tr>
<tr><td>10</td><td>1:6</td><td>+</td><td>+</td><td>257.81</td><td>16.32</td><td>6.3</td><td>5</td><td>5/15/20[#]</td><td><0.001</td></tr>
<tr><td>5</td><td>1:3</td><td>+</td><td>+</td><td>145.44</td><td>11.55</td><td>7.9</td><td>5</td><td>10/15/20[#]</td><td><0.001</td><td>ka | 0.97</td></tr>
<tr><td rowspan="4">5e</td><td>Col-0</td><td rowspan="4">60</td><td rowspan="4">10</td><td rowspan="4">1:6</td><td rowspan="4">ka</td><td>+</td><td>+</td><td>320.39</td><td>28.32</td><td>8.8</td><td>10</td><td rowspan="2">mybRNAi vs Col-0</td><td rowspan="2">0.859</td><td rowspan="4">n.d.</td></tr>
<tr><td>mybRNAi (Col-0)</td><td>+</td><td>+</td><td>324.09</td><td>29.47</td><td>9.1</td><td>3</td></tr>
<tr><td>ttg1-1 (Ler)</td><td>+</td><td>+</td><td>301.56</td><td>61.50</td><td>20.4</td><td>3</td><td rowspan="2">ttg1-1 vs tt3-1</td><td rowspan="2">0.418</td></tr>
<tr><td>tt3-1 (Ler)</td><td>+</td><td>+</td><td>339.96</td><td>37.37</td><td>11.0</td><td>3</td></tr>
</table>

For experimental procedures see methods, text and Fig. 5. The data for each subfigure and the dilution originate from independent runs. Greyshade is used to separate runs and subfigures. For *myb*RNAi relative to Col-0 and *ttg1-1* relative to *tt3-1* the diluted sample was used for kaempferol in Figure 5e. Dilutions were done with MeOH. Note that peak areas and statistics are only determined when LOQ was passed.

“n.d.” = not determined. “Name”: The name of the wildtype or mutant is give. In brackets: the background of the respective mutant is given. “na” = naringenin, “ta” = taxifolin, “ka” = kaempferol, “qu” = quercetin, “is” = isorhamnetin, “my” = myricetin, “pe” = pelargonidin, “cy” = cyaniding, “de” = delphinidin, “c” = catechin, “e” = epicatechin, “pB” = procyanidin B2. For LOD/LOQ see methods. “Normalized response” = normalized to the internal standard D_3-sakuranetin. “N”: number of replicates. “*” = for all other seedling numbers: P>0.05. Please note, this excludes seedling numbers for which LOQ is not passed. “[#]”: the 1 seedling sample was not diluted. For all, significance was determined using the Welch test.
