# Supplementary material for: A fast and simple LC-MS-based characterization of the flavonoid biosynthesis pathway for few seed(ling)s
Source: BMC Plant Biol. 2016 Sep 1;16(1):190. doi: 10.1186/s12870-016-0880-7 (PMC5007998; doi:10.1186/s12870-016-0880-7)
Supplement: Additional file 9: Table S15. — Mutants and primers. Mutants and insertion lines used in this study; Table S16. Primer; and Methods S2. (PDF 431 kb) [file 12870_2016_880_MOESM9_ESM.pdf]

Table S15. Mutants and insertion lines used in this study.

related gene	mutants/genotypes	NASC ID	mutation/insertion	background	species	citations
CHS	*tt4-11*	N2105573	T-DNA (SALK)	Col-0	*A. thaliana*	Alonso et al., 2003; Appelhagen et al., 2014; Buer et al., 2007
TT7/F3'H	*tt7-1*	N88	EMS	L*er*	*A. thaliana*	Koornneef et al., 1982; Schoenbohm et al., 2000
TT3/DFR	*tt3-1*	N84	x-rays	L*er*	*A. thaliana*	Koornneef, 1981; Shirley et al., 1992
TTG1	*ttg1-1*	-	EMS	L*er*	*A. thaliana*	Koornneeff et al., 1982
TT8	*tt8-3*	N891	T-DNA (Versailles)	Ws-2	*A. thaliana*	Nesi et al., 2000
TT2	*tt2-1*	N83	x-rays	L*er*	*A. thaliana*	Koornneef, 1981; Nesi et al., 2001
HY5	*hy5-215*	-	EMS	Col-0	*A. thaliana*	Oyama et al., 1997
PAP1,PAP2, PAP3,PAP4	*myb*RNAi	-	RNAi line	Col-0	*A. thaliana*	Gonzalez et al., 2008
PAP1	*pap1-D*	N3884	T-DNA (activation tagging)	Col-0	*A. thaliana*	Borevitz et al., 2000
COP1	*cop1-6*	N69041	EMS	Col-0	*A. thaliana*	McNellis et al., 1994

Seeds were kindly propagated and provided by several persons or ordered from ABRC (see acknowledgement) Identifiers - when available - from the Nottingham Arabidopsis Stock Centre (NASC) are given as “NASC ID”.

Table S16. Primer

mutants/genotypes	primer name	enzyme for dCAPS primer	primer sequence	citation for primer
tt4-11	ANS571	-	GATCACTCATGTCGTCTTCTG	Bowerman et al., 2012, plus SALK T-DNA primer
	ANS572	-	TTAGAGAGGAACGCTGTGCAAGACG	Bowerman et al., 2012, plus SALK T-DNA primer
tt7-1	ANS701	EcoRV	CACCAAACTCAGGAGCCAAACACATGGCATATAGATAT	this study
	ANS702	EcoRV	CGGTCTAGTCTTGAAATAGTGAGAG	this study
tt3-1	ANS588	-	ATGGTTAGTCAGAAAGAGACC	this study
	ANS589	-	CTAGGCACACATCTGTTGTGC	this study
ttg1-1	ANS584	PvuII	CTGATACGCCTTTGTTAAGAC	this study
	ANS585	PvuII	CAATACCAATCCAATCAGGCTGCGAAGAAGACCACTGCAGCT	this study
tt8-3	BJ05	-	CTTGAGTTTTGGTCATGC	this study, plus Versailles T-DNA-primer
	BJ06	-	GGACAACGAACAACATCG	this study, plus Versailles T-DNA-primer
tt2-1	ANS586	-	ATGGGAAAGAGAGCAACTACTAGTGTG	Nesi et al., 2001
	ANS587	-	TCAACAAGTGAAGTCTCGGAGC	Nesi et al., 2001
cop1-6	ANS516	KpnI, XhoI	GACAGATATTCTGTAAAGTTGCGGATG	this study
	ANS513	KpnI, XhoI	CTTTTGGAGGAGGTAACATTCTTGTAAATCATGGTAC	this study
hy5-215	ANS546	PsiI	GTTTGAAACTTTTCGTCTAAAGTCTCTTTTATGTTTTATA	this study
	ANS522	PsiI	GTAAAAATGTAAATTCAATGAATAG	this study

For designing dCAPS primers dCAPSfinder 2.0 was used (Neff et al., 2002)

Methods S2

Experiments with external standards

Precision and linearity were analysed using ten replicates of the standard mix (500 nM). The stability in dependence of FA and time was tested in triplicates with 500 nM per substance. In all cases D3-quercetin and D3-sakuranetin at final concentrations of 400 and 5 nM, respectively, were included. For spiking experiments, 10 µl of the individual external standards were added at the following concentrations: 75 µM (extracted samples), 12 µM (non-extracted samples) for seeds and 20 µM for seedlings. External standards were combined with 100 µM D3-quercetin (extracted seed samples), 200 nM D3-sakuranetin (non-extracted seed and all seedling samples).

LC-MS (detailed)

A KINETEX 2.6 µm C18 100 Å (4.6 mm x 50 mm) C18 column (Phenomenex, http://www.phenomenex.com) equipped with supplier's appropriate pre column was used for all samples. For all experiments, a QC was injected at least all 10 samples flanked by MeOH samples.

LC-ESI-MS-QTRAP(MRM): 10 µl of flavonoid standard or sample were separated by HPLC (1260 HPLC, Agilent, http://www.agilent.com/home) system applying a gradient (A: 0.1 % FA in water; B: 0.1 % FA in MeOH; flow rate 500 µl min^{-1}) with 5 % B for 0.5 min, a 2.5 min gradient to 100 % B, maintained for 3 min and returned to 5 % B for re-equilibration resulting in a total runtime of 10 min. Eluted substances were detected in positive ion mode with multiple reaction monitoring (MRM) using a QTRAP 5500 mass spectrometer equipped with a TurboV Source (ABSCIEX, http://sciex.com). The capillary voltage was set to 5.5 kV. The collision energy was optimized (30-61 eV) for each transition. For each flavonoid species, two MRM transitions were recorded (Table S2).

LC-ESI-MS-QTOF(AutoMSMS): 10 µl of sample (if not stated otherwise) was injected into a DIONEX 3000 RSL UPLC (Thermo Fisher, http://www.dionex.com) system and separated with the following gradient (A: 0.1 % FA in water; B: 0.1% FA in MeOH; flow rate 300 µl*min^{-1}) starting with 5 % B for 0.1 min followed by a 4 min gradient to

100 % B, maintained for 2 min and returned to 5 % B for re-equilibration. Mass-spectra were recorded on a maXis 4G (Bruker Daltonic, www.bruker.com) equipped with an ESI source. Samples were measured in positive ion mode in the range m/z 50-1000 at 3 spectra per second, capillary voltage was set to 4.5 kV, drying gas was set to 220°C at 12 L/min and rolling collisions energy was used. MS/MS data was recorded for selected flavonoids with a signal intensity above 20,000 counts.

LC-ESI-MS-QTOF (pseudoMS^3):

For pseudoMS^3 determinations the same LC conditions were used as for the AutoMSMS conditions. Mass-spectra were recorded on a maXis 4G (Bruker Daltonic, www.bruker.com) equipped with an ESI source. Samples were measured in positive ion mode in the range m/z 50-1000 at 3 spectra per second, capillary voltage was set to 4.5 kV and drying gas was set to 220°C at 12 L/min. In difference to the AutoMSMS runs, in-source collision induced dissociation (ISCID) was used. ISCID energy was set to 60V - 80V. MS/MS data was recorded for selected flavonoids with a signal intensity above 20,000 counts
